# Interaction variability shapes succession of synthetic microbial ecosystems

**DOI:** 10.1038/s41467-019-13986-6

**Published:** 2020-01-16

**Authors:** Feng Liu, Junwen Mao, Wentao Kong, Qiang Hua, Youjun Feng, Rashid Bashir, Ting Lu

**Affiliations:** 1 Department of Bioengineering, University of Illinois at Urbana-Champaign, Urbana, IL USA; 2 Carl R. Woese Institute for Genomic Biology, University of Illinois at Urbana-Champaign, Urbana, IL USA; 3 State Key Laboratory of Bioreactor Engineering, East China University of Science and Technology, Shanghai, China; 4 School of Bioengineering, East China University of Science and Technology, Shanghai, China; 5 Department of Physics, Huzhou University, Huzhou, China; 6 School of Medicine, Zhejiang University, Hangzhou, China; 7 Micro and Nanotechnology Laboratory, University of Illinois at Urbana-Champaign, Urbana, IL USA; 8 Carle Illinois College of Medicine, Urbana, IL USA; 9 Department of Physics, University of Illinois at Urbana-Champaign, Urbana, IL USA; 10 Center for Biophysics and Quantitative Biology, University of Illinois at Urbana-Champaign, Urbana, IL USA; 11 National Center for Supercomputing Applications, University of Illinois at Urbana-Champaign, Urbana, IL USA

**Keywords:** Microbial ecology, Synthetic biology

## Abstract

Cellular interactions are a major driver for the assembly and functioning of microbial communities. Their strengths are shown to be highly variable in nature; however, it is unclear how such variations regulate community behaviors. Here we construct synthetic *Lactococcus lactis* consortia and mathematical models to elucidate the role of interaction variability in ecosystem succession and to further determine if casting variability into modeling empowers bottom-up predictions. For a consortium of bacteriocin-mediated cooperation and competition, we find increasing the variations of cooperation, from either altered labor partition or random sampling, drives the community into distinct structures. When the cooperation and competition are additionally modulated by pH, ecosystem succession becomes jointly controlled by the variations of both interactions and yields more diversified dynamics. Mathematical models incorporating variability successfully capture all of these experimental observations. Our study demonstrates interaction variability as a key regulator of community dynamics, providing insights into bottom-up predictions of microbial ecosystems.

## Abstract (web summary)

Cellular interactions are a major driver of microbial communities and shown highly variable in strength. Here the authors construct synthetic consortia and mathematical models to elucidate the role of interaction variability in driving ecosystem succession.

## Introduction

Microbial communities are assemblages of multi-species microorganisms that live and interact with each other. They regulate the biogeochemical cycling of the planet^1^, fertilization of crops^2^, and metabolism of our human body^3^, thereby affecting profoundly the environment, agriculture and human health. As the dynamics of a community often underlies its emergent properties, being able to predict ecosystem succession is central to the elucidation of community organization and function^4^, rational design of artificial ecosystems^5–7^ and introduction of intervention^8^. To that end, rapidly developed is bottom-up analysis^9–12^, which aims to determine community behaviors from the characterization of microbial interactions—such as competition and cooperation—that are ubiquitous among microbes^13^. This approach is conceptually compelling, because it offers a systematic solution to capture emergent ecosystem properties and is potentially generalizable for both native and synthetic communities. In practice, although there are exciting successes in selected cases such as the utilization of the generalized Lotka-Volterra model for specific microbiomes^14–16^, predicting community dynamics from the bottom up remains as a grand challenge in general^4^.

One possible major cause of this challenge is pointed to the discrepancy between current modeling scheme and experimental observations. To date, bottom-up models commonly consider microbial interactions invariant and presume that determining interaction strength autonomously specifies community behaviors. Associated with these model developments are experimental efforts, which largely focus on the identification and measurement of interaction strength^4,13^. However, increasingly overwhelming evidences show that microbial interactions are highly variable, rather than static, in nature. Indeed, microbial interactions often change with environmental cues such as pH, nutrient and stress^17–27^. For example, the mutualism between *Escherichia coli* and *Rhodopseudomonas palustris* is moderated by the toxicity of organic acids in the culture^21^; the antagonism from *Pseudomonas aeruginosa* to *Staphylococcus aureus* increases with ion depletion^24^. Microbial interactions are also subjective to the population of microorganisms generating the interactions as well as the presence of other species^20,28–32^. For instance, *Lactococcus lactis* produces nisin to suppress pathogens such as *Staphylococcus aureus* through quorum sensing of its own population^31^ while *Enterococcus faecalis* secrets cytolysin when sensing the presence of target cells^32^. Additionally, as cellular interactions are typically fulfilled through the production of metabolites and proteins—biochemical processes that are fundamentally stochastic^33–35^, there are intrinsic fluctuations for all microbial interactions including those ‘constant’. Recognizing this characteristic of microbial interactions, a handful of mathematical frameworks have been proposed to consider interaction variations^36–40^. In contrast, there is a lack of systematic experimental investigations that quantify the degree of variability for given microbial interactions. It also remains unclear to what extent such variations drive ecosystem succession and alter community structures and characteristics. Accordingly, it is unknown how the incorporation of variability into modeling shapes the predictive power of bottom-up mathematical modeling.

Here we hypothesize that variations of microbial interactions are a key modulator of community behaviors and characterizing and incorporating the variability empowers predictive understanding of ecosystem succession from the bottom up. To test the hypotheses, we design and build a set of synthetic three-strain microbial consortia, which involve both cooperation and competition, and use them as our experimental model systems. Compared to native ecologies, such synthetic communities possess a significantly reduced degree of complexity while offering the feasibility for mechanistic dissection and quantitative measurement^7,41–49^. In parallel, we develop mathematical models with an explicit incorporation of interaction variability to analyze ecosystem succession. For the consortium containing a variable cooperation, we quantify the variability of cooperation, elucidate the alteration of ecosystem dynamics arising from the variations, and demonstrate the power of variability-incorporated modeling in capturing community development. For the ecosystem whose cooperation and competition both fluctuate, more complex ecosystem dynamics arises but characterizing the variabilities again lead to successful succession predictions. Together, our results elucidate the role of interaction variability in regulating community dynamics, providing fundamental insights into bottom-up understanding of microbial ecosystem succession.

## Results

### Creation of a cooperation between synthetic populations

**Fig. 1:**
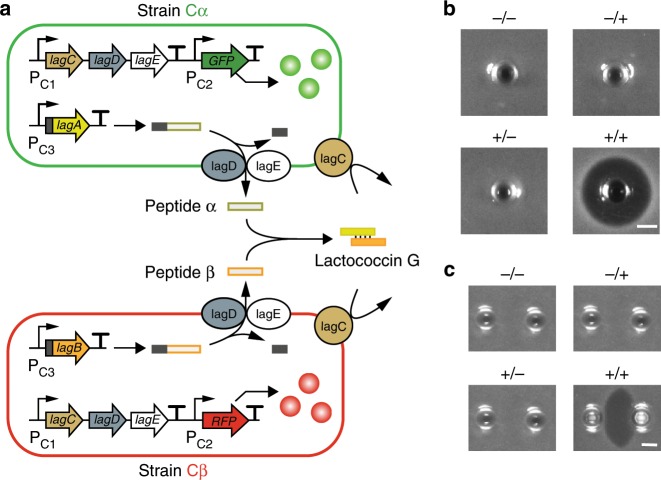
An engineered cooperation between two bacterial strains. **a** Circuit design. The cooperative consortium consists of two *L. lactis* strains, Cα and Cβ. Cα carries three constitutively expressed genes *lagA*, *lagD* and *lagE*, allowing to synthesize and secret the α subunit of the bacteriocin lactococcin G (lcnG). Cβ harbors three constitutively expressed genes *lagB*, *lagD* and *lagE*, which enables the synthesis and secretion of the β subunit of lcnG. The two subunits, α and β, autonomously assemble into lcnG that inhibits *L. lactis* strains. Meanwhile, Cα and Cβ both carry *lagC*, the lcnG immunity gene, and respectively have the fluorescence reporter genes *yemGFP* and *mCherry*. **b** Single-well inhibition zone assays. The four wells were loaded with blank culture (GM17 media supplemented with chloramphenicol) (−/−), blank culture and Cβ supernatant (−/ + ), Cα supernatant and blank culture ( + /−), and Cα and Cβ supernatants ( + / + ) accordingly. *L. lactis* NZ9000 loaded with a chloramphenicol resistant gene was used as an indicator strain. An inhibition zone was observed around the ( + / + ) well containing the both supernatants. **c** Double-well inhibition zone assays. The left and right wells were loaded with blank culture and blank culture (−/−), blank culture and Cβ supernatant (−/ + ), Cα supernatant and blank culture ( + /−), and Cα and Cβ supernatants ( + / + ) accordingly. *L. lactis* NZ9000 loaded with a chloramphenicol resistant gene was used as an indicator strain. An inhibition area was observed between the wells loaded with the Cα and Cβ supernatants respectively. In panels **b** and **c**, scale bars, 3 mm.

We started by engineering a cooperation in synthetic populations because it is ubiquitous among microorganisms and critical to their organization^50–52^. Specifically, the interaction involves two engineered *Lactococcus lactis* strains, Cα and Cβ, both of which harness the biosynthetic pathway of lcnG, a Class II two-subunit lactococcus bacteriocin (Fig. 1a and Methods)^53,54^. Here, Cα constitutively expresses *lagA*, *lagD*, and *lagE* which encode the peptide α precursor, ABC transporter and accessory protein of the pathway respectively, allowing the strain to synthesize and secret the α subunit of lcnG. Similarly, Cβ constitutively expresses the pathway’s β precursor gene *lagB*, transporter gene *lagD* and accessory protein gene *lagE*, enabling the synthesis and secretion of the β peptide, the other subunit of lcnG. In the extracellular milieu, the two subunits α and β self-assemble into a bioactive antimicrobial which inhibits the growth of *L. lactis* strains. Through this fashion of division of labor, Cα and Cβ achieve a cooperation for successful lcnG production. As lcnG inhibits all *L. lactis* strains, the immunity gene *lagC* was introduced into both Cα and Cβ to confer them an immunity. Additionally, two reporter genes, *yemGFP* and *mCherry*, were loaded into Cα and Cβ respectively to enable the quantification of ecosystem dynamics.

To validate the cooperation, we conducted inhibition zone assays using the supernatants of Cα and Cβ monocultures (Methods). Four supernatant combinations, including blank culture (GM17 media supplemented with chloramphenicol) (−/−), supernatant of Cα monoculture ( + /−), supernatant of Cβ monoculture (−/ + ), and mix of the two supernatants ( + / + ), were loaded into single wells in the solid agar plated with a lawn of lcnG-sensitive cells (*L. lactis* NZ9000 loaded with a chloramphenicol resistant gene)^55^. Upon 8 hours (h) of incubation at 30 °C (Methods), only the well loaded with the supernatant mix ( + / + ) produced an inhibition zone (Fig. 1b). In addition, the four supernatant combinations were loaded into separate wells in agar plates covered with lcnG sensitive cells. We found that a clear inhibition zone formed between adjacent wells only when they were loaded with the Cα and Cβ supernatants respectively (Fig. 1c). Together, the results confirmed two pieces of information. First, combination of Cα and Cβ produced the active lcnG but individual strains alone did not. Second, peptides α and β were able to autonomously assemble into an active bacteriocin without the need for any assistance.

### Characterizing the variations of cooperation

**Fig. 2:**
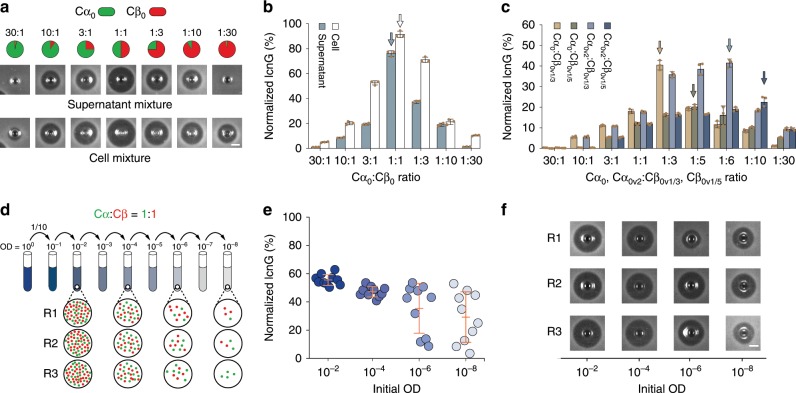
Characterization of cooperation variations. **a** Representative inhibition zones formed by different ratios of supernatant mixes from the Cα_0_ and Cβ_0_ monocultures (top row) and the supernatants of the Cα_0_-Cβ_0_ co-cultures growing from different initial ratios (bottom row). **b** Relative lcnG levels in the monoculture supernatant mixes (white bars) and the co-culture supernatants (blue bars) (*n* = 3). Here, the lcnG concentration is normalized by the lcnG productivity of the single lcnG-producing strain Cαβ. **c** Normalized lcnG levels in the supernatant mixes of monoculture Cα_0_ and Cβ_0_ variants (*n* = 3). Cα_0v2_ is a Cα variant that has a doubled peptide α productivity compared to Cα_0_. Cβ_0v1/3_ and Cβ_0v1/5_ are two Cβ_0_ variants, whose peptide β productivities are 1/3 and 1/5 of that of Cβ_0_. **d** Schematic illustration of population fluctuations during serial dilution. Variations of the cooperator ratio increase with reducing initial cell density, resulting in different abundances across replicates (R1, R2, and R3). **e** Normalized lcnG concentration of the Cα-Cβ co-culture from different initial ODs but a fixed 1:1 ratio. Each circle represents the lcnG level from a single experiment. For each OD, there are a total of 10 replicates (*n* = 10). **f** Representative inhibition zones formed by the consortium growing from different initial ODs. In panel **b**, **c**, and **e**, bars and error bars correspond to means and s.d. In panels **a** and **f**, scale bars, 3 mm. Source Data available in the source data file.

Driven by diverse biotic and abiotic factors, microbial interactions are highly variable. Here we aimed to experimentally determine the variability of the cooperation originating from its division of labor nature. Specifically, to quantitate how labor partition, reflected by the cooperator ratio, affects the strength of cooperation, we grew the monocultures of Cα_0_ and Cβ_0_ (the reporter-free version of Cα and Cβ), mixed their supernatants with varied ratios while fixing the total volume (30 μL), and further used the mixes to perform inhibition zone experiments (Methods). Our results (Fig. 2a, top row) showed that, across the ratios from 30:1 to 1:30, the size of the inhibition zones varies from small to large and back to small with the largest occurring at the 1:1 ratio. As zone size correlates positively to a mix’s lcnG level and hence the strength of cooperation, the results suggested that initial labor partition can induce significant variations of the cooperation. Using the supernatants of Cα_0_-Cβ_0_ co-cultures with varied initial relative population abundance, we also observed the same dependence of the lcnG level on the initial population partition (Fig. 2a, bottom row). To quantitatively determine the variations, we further measured the relative lcnG level of each sample by normalizing its inhibition zones with that of Cαβ, a lcnG-producing strain (*L. lactis* MG1363 loaded with the complete lcnG pathway) (Fig. 2b, Methods, Supplementary Fig. 1).

In the above experiments, a low-high-low pattern of the cooperation strength (i.e., lcnG productivity) was observed with the maximum at the 1:1 labor partition. From a molecular perspective, we reasoned that the bell-shape variation of the cooperation is rooted in the 1:1 stoichiometric ratio of the α and β subunits. We further speculated that the 1:1 initial ratio happened to be optimal because Cα_0_ and Cβ_0_ have a comparable growth rate and a comparable subunit productivity and, thus, the final ratio of the two peptides in a co-culture is solely determined by their initial densities. Supporting the speculation, the inhibition zone assays and lcnG quantifications for the Cα-Cβ co-culture (Supplementary Fig. 2b, c) showed that the maximum was shifted towards a higher initial Cβ abundance due to a slower growth rate of Cβ than Cα (Supplementary Fig. 3a). Such a growth reduction is a common consequence of heterologous protein production in microorganisms that is well documented in literature^56,57^. Meanwhile, the maximum for the monoculture mixes remained at 1:1 ratio (Supplementary Fig. 2a, c) because, in monocultures, Cα and Cβ had the same amount of nutrient and, thereby, produced a comparable level of subunits. To further confirm that the maximal cooperation is characterized by the stoichiometric ratio of the lcnG subunits, we derived new strains with altered peptide productivities, including Cα_0v2_ with a doubled α productivity compared to Cα, and Cβ_0v1/3_ and Cβ_0v1/5_ whose β productivities are reduced to 1/3 and 1/5 of that of Cβ_0_ (Supplementary Fig. 4). In theory, the combinations of Cα_0_ and Cβ_0v1/3_, Cα_0_ and Cβ_0v1/5_, Cα_0v2_ and Cβ_0v1/3_, and Cα_0v2_ and Cβ_0v1/5_ would shift the optimal ratios from 1:1 to 1:3, 1:5, 1:6, and 1:10 respectively, which were subsequently confirmed by the experiments (Fig. 2c).

For any microbial ecosystems, there are intrinsic random fluctuations of cellular populations arising from various stochastic processes^33–35,58,59^, which motivated us to quantify the variability of cooperation from intrinsic stochasticity by specifically examining the effects of sampling of initial populations (i.e., genetic drift)^60,61^. We first inoculated the consortium from an initial culture (1:1 Cα-to-Cβ ratio, 1.0 optical density at 600 nm (OD)) into fresh media through serial 1:10 dilution to generate samples with different initial ODs (10^−2^, 10^−4^, 10^−6^ and 10^−8^) (Fig. 2d and Methods). Here, 10^−8^ was selected as the minimal initial OD since it is the minimal density for both Cα and Cβ to stably grow in monocultures (Supplementary Fig. 5). Next, we grew the cultures for the defined incubation time, collected their supernatants, and measured the lcnG levels (Methods). Figure 2e shows the lcnG levels of the co-cultures normalized by the productivity of Cαβ, a single-strain lcnG producer we created. We found that, with the reduction of initial OD, the mean lcnG productivity of the consortium decreased monotonically but, in the meanwhile, the sample-to-sample variation increased. Such a trend was also clearly observed in the inhibition zones formed by the co-cultures (Fig. 2f and Supplementary Fig. 6). By conducting the same experiments for the reporter-free version of the consortium, the Cα_0_-Cβ_0_ ecosystem (Supplementary Fig. 7), we confirmed that random sampling continued to serve as a key inducing factor of the variation.

Notably, partition alteration and random sampling are two independent sources of cooperation variations; however, they are intrinsically connected. The both alter cooperation strength by varying the division of labor among the cooperators, but the former is a controlled, deterministic alteration of the partition while the latter is unintended, stochastic alteration.

### Ecosystem successions driven by cooperation variations

**Fig. 3:**
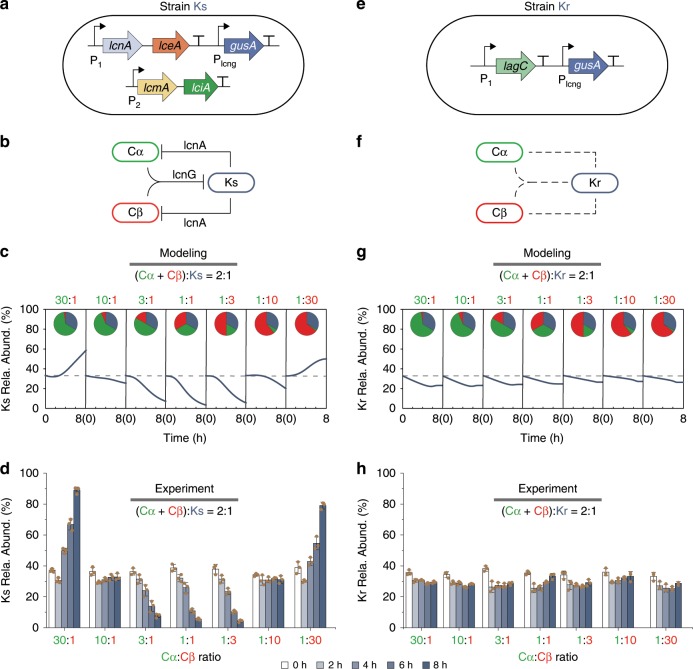
Succession of a three-strain ecosystem driven by cooperation variations from labor partition. **a** Design of a competitive third strain Ks. The lcnA biosynthesis pathway, including the genes *lcnA, lceA*, *lciA,* and *lcmA*, is placed under constitutive promoters for constant lcnA secretion. The gene *gusA3* is also introduced for colorimetric quantification. **b** A three-strain consortium composed of Cα, Cβ, and Ks. Cα and Cβ cooperate to produce lcnG that inhibits Ks; in turn, Ks secrets lcnA to oppose Cα and Cβ. **c**, **d** Model-predicted (**c**) and experimentally measured (**d**) temporal dynamics of the Ks abundance in the Cα-Cβ-Ks ecosystem. The initial Cα:Cβ partition was varied across 30:1 to 1:30, but the total (Cα + Cβ): Ks ratio was fixed as 2:1. **e** Design of a control strain Kr. *L. lactis* MG1363 is loaded with the constitutively expressed *lagC*, the lcnG immunity gene, to confer resistance to lcnG and *gusA3* for colorimetric quantification. **f** A three-strain ecosystem composed of Cα, Cβ and Kr. As Kr is deficient in lcnA production but resistant to lcnG, the consortium does not have active bacteriocin-mediated interactions. **g**, **h** Model predictions (**g**) and experimental measures (**h**) of temporal dynamics of the Kr strain in the Cα-Cβ-Kr ecosystem. The initial Cα:Cβ partition was varied across 30:1 to 1:30, but the total (Cα + Cβ): Kr ratio was fixed as 2:1. Source Data available in the source data file.

To elucidate the consequences of interaction variations on community behaviors, we designed a three-strain consortium composed of the two cooperators (Cα and Cβ) and a competitive third strain (Ks). Here, Ks is an engineered strain capable of constitutively secreting lactococcin A (lcnA)^62^, a bacteriocin that effectively kills all *L. lactis* strains including Cα and Cβ unless immunized. Experimentally, Ks was built by introducing the lcnA pathway into the *L. lactis* MG1363 (Fig. 3a). To efficiently count Ks in the three-strain ecosystem, it was inserted with constitutively expressed *gusA3*, a beta-glucuronidase gene that enables colorimetric quantification upon the supplementation of X-Gluc^63^. As Ks does not contain the lcnG immunity gene *lagC*, it is sensitive to lcnG cooperatively produced by Cα and Cβ. Therefore, the community involves a cooperation between Cα and Cβ and a competition of Ks with Cα and Cβ (Fig. 3b).

Meanwhile, we hypothesized that predicting ecosystem behaviors requires mathematical models that take in account the variability of interaction. To test the hypothesis, we devised a dynamic ecosystem model using a systematic, bottom-up fashion (Methods, Supplementary Information 1). Briefly, we first constructed models of Cα and Cβ monocultures (Supplementary Equations 1-2, Supplementary Fig. 3b), and used them as modules to derive a model of Cα-Cβ co-culture incorporating variability (Supplementary Equation 4) with their parameters specified with experiments (Supplementary Fig. 8). We then constructed a model of lcnA-producing Ks monoculture (Supplementary Equation 5) and determined its parameters experimentally (Supplementary Fig. 9a, b). Finally, by merging the models of the Cα-Cβ co-culture model with the Ks monoculture and characterizing the inhibitions between the modules (Supplementary Equations 6-7, Supplementary Fig. 9c-f), we obtained a model for the Cα-Cβ-Ks consortium (Supplementary Equation 9). For batch fermentations starting from a fixed (Cα + Cβ):Ks ratio (2:1), the model predicted that the ecosystem evolves temporally into Ks dominance at imbalanced cooperator partitions and Ks subordinate at close partitions (Fig. 3c), suggesting that the variations of cooperation can modulate the succession of the consortium dramatically.

To test the predictions, we experimentally assembled the consortium by mixing Cα, Cβ and Ks with altered initial ratios but a fixed total OD (10^−2^) as our model construction (Methods). Consistent with the predictions, our fermentations showed that the Ks percentage indeed increased over time and eventually became dominant when the Cα:Cβ ratio was extremely imbalanced (30:1 and 1:30); in contrast, when their abundances were close (3:1, 1:1, and 1:3), the Ks percentage declined monotonically and diminished eventually (Fig. 3d). Opposite with the Ks percentage, the relative abundances of Cα and Cβ both decreased at imbalanced Cα:Cβ ratios (30:1 and 1:30) but increased when the ratio is close (3:1, 1:1, and 1:3) (Supplementary Fig. 10a, b). Meanwhile, although the total OD of the ecosystem remained largely consistent regardless of the Cα:Cβ ratios (Supplementary Fig. 8, circles), the lcnG level changed significantly (Supplementary Fig. 8, bars): it remained limited throughout the fermentations in imbalanced scenarios but accumulated rapidly at the balanced cases, consistent with our previous characterization (Fig. 2a–c). The correspondence between higher lcnG production (Supplementary Fig. 8) and lower Ks abundance (Fig. 3c, d) suggested a strong correlation between cooperation variations and diversified ecosystem succession.

To further confirm that it is a causal relationship between interaction variations and diversified succession, we designed a Ks variant, named Kr, which is resistant to lcnG co-produced by Cα and Cβ and deficient in producing lcnA. Experimentally, Kr was established by removing the lcnA biosynthetic pathway from Ks while introducing the lcnG immunity gene *lagC* (Fig. 3e, Supplementary Fig. 11). The mixture of Cα, Cβ and Kr formed a control consortium where the cooperation and competition are both abolished (Fig. 3f). Our mathematical model (Methods, Supplementary Equation 11) predicted that, upon the same alterations of the initial Cα:Cβ ratio, the structure of the consortium remains largely invariant (Fig. 3g), which was subsequently verified by co-culture experiments (Fig. 3h, Supplementary Fig. 10c, d). In this case, varying initial cooperator partition continued to generate the variation of lcnG production as in the Cα-Cβ-Ks consortium (Fig. 2a–c); however, due to Kr’s resistance to lcnG, the variation was ‘insulated’ and not propagated to ecosystem succession, leading to the invariant community structure. These results confirmed that it is indeed the variations of interaction that cause the diversification of ecosystem development.

To examine if the modulation of ecosystem succession by interaction variability is specific to the origin of variation, we investigated the dynamics of the Cα-Cβ-Ks consortium upon fluctuations in sampling, another source of variation we characterized. Mathematically, we used a bottom-up strategy to create a corresponding dynamic model by introducing sampling-induced variations into the previous Cα-Cβ co-culture module (Supplementary Equation 4) and Ks monoculture module (Supplementary Equation 5) with experimentally derived parameters (Supplementary Tables 7 and 8) and combining the resulting modules into a single model (Methods). Using the model that encapsulates variations from sampling, we conducted multiple repeats of computational batch fermentations for the consortium starting with the 1:1:1 ratio but different initial ODs. In parallel, we experimentally mixed the strains Cα, Cβ and Ks in 1:1:1 ratio to form the consortium, inoculated them into ten replicates at specific initial total ODs (10^−2^, 10^−4^, 10^−6^, and 10^−8^) and measured their population dynamics over time (Methods). Notably, due to the difference in initial conditions, the culturing time and sampling time were altered accordingly to enable a consistent and proper comparison (Supplementary Fig. 12).

**Fig. 4:**
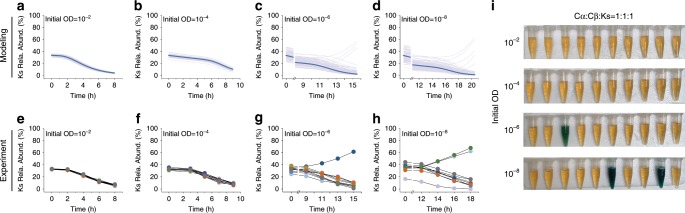
Divergent ecosystem dynamics directed by cooperation variations from sampling. **a–d** Model-predicted time courses of Ks abundance in the Cα-Cβ-Ks ecosystem starting with a total initial OD of 10^-2^ (**a**), 10^−4^ (**b**), 10^−6^ (**c**), and 10^−8^ (**d**). For each initial OD, a total of 100 simulation replicates were shown. For comparison, the deterministic dynamics of Ks without sampling-induced variability were displayed with bold lines. **e**–**h** Experimentally measured population dynamics of Ks in the Cα-Cβ-Ks ecosystem for a total initial OD of 10^−2^ (**e**), 10^−4^ (**f**), 10^−6^ (**g**), and 10^−8^ (**h**). The co-cultures were inoculated at 1:1:1 ratio but different total ODs; for each initial OD, 10 experimental replicates were performed. **i** Colorimetric assays of the Cα-Cβ-Ks co-cultures from the experiment in **e**–**h**. Cultures containing strain Ks turned into blue green due to the cleavage of X-Gluc by beta-glucuronidase from Ks; in contrast, those lacking Ks remains the original culture color (yellow). Each test tube contains only the supernatants of the co-culture after X-Gluc treatment. Source Data available in the source data file.

Our model predictions and subsequent experiments showed that the Ks fraction consistently declined over time at high initial ODs (e.g., 10^−2^ and 10^−4^) across all replicates (Fig. 4a–f), suggesting that Cα and Cβ robustly outperformed Ks. Conversely, when the initial OD was low (10^−6^ and 10^−8^), the consortium exhibited two divergent modes of succession (Fig. 4c–d, g–h): the Ks fraction declined monotonically as in the high initial OD cases; alternatively, it increased over time and dominated the population. Furthermore, comparison of all four cases suggested that the chance of Ks dominance increased with reducing initial OD. To directly visualize such succession outcomes, we further collected the co-cultures at the end of fermentations and performed colorimetric assays by adding X-Gluc to the co-cultures (Methods). Because Ks encodes beta-glucuronidase which can produce a clear blue green color, the colors of the treated supernatants (Fig. 4i) reflected the Ks dominance in individual experiments. Linking to the characterization of sampling-induced variations (Fig. 2e, Supplementary Tables 7, 8), these results confirmed that increasing sampling-induced variations equally drives the consortium into divergent outcomes. Supporting the statement, we computationally turned off the sampling-induced variations in our mathematical model and found that, without the variations, Ks persistently declined regardless of initial ODs (Fig. 4a–d, bold lines). The theory-experiment consistency suggested that incorporating variability into ecosystem modeling provides a predictive capacity over community behaviors.

### Construction of pH-dependent competition and cooperation

**Fig. 5:**
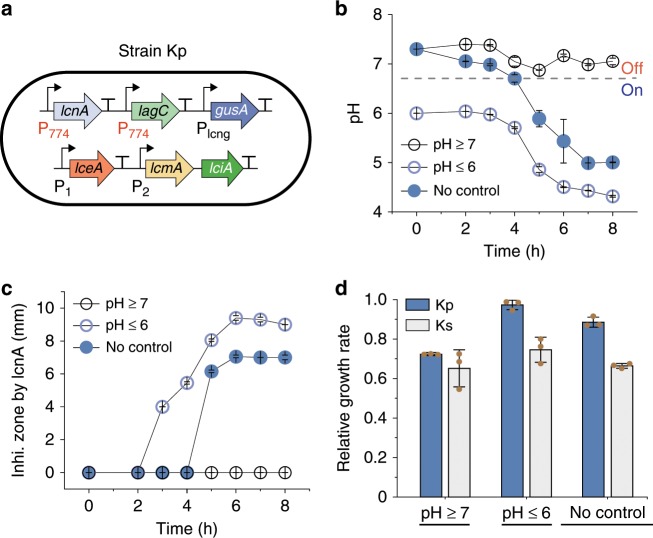
A synthetic strain whose interactions vary with environmental pH. **a** Design of the strain Kp. The lcnA precursor gene, *lcnA*, is driven by the pH-dependent promoter P_774_ while other lcnA biosynthesis genes *lceA*, *lciA,* and *lcmA* are under the control of native constitutive promoters P_1_ and P_2_, leading to a pH-modulated production of lcnA. Meanwhile, *lagC*, the lcnG immunity gene, is regulated by the promoter P_774_, resulting in pH-dependent resistance to lcnG. Additionally, the reporter gene *gusA3* was introduced to Kp to enable quantification. **b**, **c** Temporal profiles of extracellular pH (**b**) and the sizes of lcnA inhibition zone (**c**) of Kp in three pH-defined conditions: pH ≥ 7, pH ≤ 6 and no pH control. **d** Relative growth rates of Kp and Ks in GM17 media mixed with the supernatant of Cα-Cβ co-culture normalized by those growing in the media mixed with the Cα-Cβ’ supernatant in three pH-defined conditions. Source Data available in the source data file.

To examine if variability-modulated ecosystem succession is general to different cellular interactions, we designed a new cooperation-competition consortium composed of the cooperators—Cα and Cβ—and Kp, a strain that opposes Cα and Cβ and resists their killing in a pH-dependent manner. Using *L. lactis* MG1363 as the host, the pH-dependent Kp-to-Cα/Cβ inhibition was created by applying a pH-inducible promoter P_774_^64^ to control *lcnA*, the precursor gene of the lcnA pathway and the constitutive promoters P_1_ and P_2_ to drive the rest genes (*lceA*, *lcmA*, and *lciA*) in the pathway (Fig. 5a). Similarly, the pH-dependent resistance to Cα/Cβ-to-Kp inhibition was enabled by using the promoter P_774_ to drive *lagC*, the immunity gene of lcnG co-produced by Cα and Cβ. Additionally, *gusA3*^63^ was constitutively expressed to enable colorimetric quantification of the strain.

Previous studies showed that the promoter P_774_ is active when the environmental pH is below 6.5 but switched to be inactive when above 7^64^. Thus, Kp’s lcnA production (i.e., inhibition over Cα and Cβ) and LagC production (i.e., resistance to killing by Cα and Cβ) are no longer constant but, instead, vary with the environment. Importantly, these strains are all derived from *L. lactis* which naturally produces a large amount of lactic acid and, thus, can lower the pH of culture in fermentation, which suggests that both the Kp-to-Cα/Cβ and Cα/Cβ-to-Kp inhibitions can be highly dynamic even in simple batch fermentation.

To validate the interactions, we grew Kp monoculture under three settings: pH ≥ 7, pH ≤ 6, and no pH control (Methods) (Fig. 5b and Supplementary Fig. 13). Our results (Fig. 5c and Supplementary Fig. 14) showed that the size of the lcnA inhibition zones (Methods) remains undetectable during the pH ≥ 7 fermentation, suggesting no lcnA production. By contrast, in the pH ≤ 6 fermentation, lcnA was detected as early as 3 h after fermentation and the culture yielded the highest lcnA level. For the case of no pH control, lcnA was detected after 4 h of fermentation and eventually accumulated to a medium level. These results confirmed that lcnA production (Kp-to Cα/Cβ inhibition) is highly correlated with the environmental pH. To confirm the pH-dependence of the Cα/Cβ-to-Kp inhibition, we cultured Kp and Ks in pH-defined media mixed with the supernatant of the corresponding Cα-Cβ or Cα-Cβ’ co-cultures (Cβ’ is a Cβ variant deficient in β subunit production), and compared their relative growth rates (Methods, Supplementary Fig. 9e, f and 15). The results (Fig. 5d) showed that Kp grew better than Ks in all three conditions despite a higher load due to lagC production, demonstrating that Kp gained a resistance to lcnG. Additionally, the results (Fig. 5d, blue bars) showed that Kp grew best at pH ≤ 6 and worst at pH ≥ 7, confirming pH modulates Kp’s resistance to lcnG. Equivalently, the results suggested that both the growth of Cα and Cβ monoculture (Supplementary Fig. 16) and the strength of Cα-Cβ cooperation are indeed modulated by pH (Supplementary Fig. 17).

**Fig. 6:**
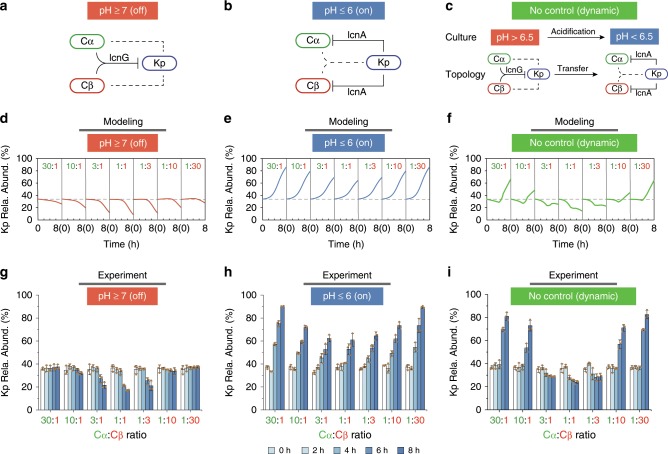
Ecosystem dynamics jointly regulated by cooperation variations and competition variations. **a**–**c** Interaction topology of the Cα-Cβ-Kp ecosystem in different pH settings. At pH ≥ 7 (**a**), the promoter P_774_ remains inactive (i.e., ‘Off’ state), which abolishes Kp-to-Cα/Cβ inhibition while allowing Cα and Cβ to suppress Kp. At pH ≤ 6 (**b**), the promoter P_774_ is active (i.e., ‘On’ state), leading to effective Kp-to-Cα/Cβ inhibition but diminished Cα/Cβ-to-Kp suppression. Where there is no pH control (**c**), the promoter dynamically transits from inactive to active (i.e., ‘Dynamic’ state) with pH reduction upon fermentation. As a result, the interaction network switches from Cα/Cβ killing Kp to Kp killing Cα/Cβ. **d**-**i** Model-predicted (**d**–**f**) and experimentally measured (**g**–**i**) temporal evolution of Kp abundance in three pH-defined settings and under varied initial Cα:Cβ partitions. Although Cα:Cβ ratio was varied from 30:1 to 1:30, the total Cα and Cβ concentration was kept at 2:1 ratio with the Kp concentration. Source Data available in the source data file.

These results further implied that, subject to environmental pH, the Cα-Cβ-Kp ecosystem can exhibit multiple modes of interaction. When pH ≥ 7, Cα and Cβ cooperatively inhibit Kp (Fig. 6a); when pH ≤ 6, Kp inhibits Cα and Cβ (Fig. 6b); by contrast, when there is no pH control, Cα and Cβ inhibit Kp initially but, later, are suppressed by Kp (Fig. 6c).

### Dynamics jointly regulated by multiple interaction variations

To illustrate how the Cα-Cβ-Kp consortium evolves upon both cooperation and competition variations, we assembled a dynamic community model (Supplementary Equation 17) from the bottom up (Methods). Then we used the model to explore the succession of the consortium when its interaction strengths vary due to simultaneous pH and cooperator partition alterations. In parallel, we performed Cα-Cβ-Kp co-culture experiments under the conditions identical to the computational test (Methods).

For the consortium starting from a fixed initial (Cα + Cβ):Kp ratio (2:1), the model predicted and subsequent experiments confirmed that, when pH was controlled above 7, Kp abundance declined gradually over fermentation for different initial Cα:Cβ ratios but reached to the lowest at 1:1 (Fig. 6d, g). In contrast, when pH was below 6, Kp became increasingly dominant over time for all Cα:Cβ ratios but augmented the most at unbalanced cases (30:1 and 1:30) (Fig. 6e, h). When there was no pH control, the consortium succession displayed two distinct patterns: At unbalanced ratios, Kp evolved to be dominant as the case of pH ≤ 6 but, at close ratios, Kp abundance declined over time as the pH ≥ 7 case (Fig. 6f, i).

Although seemingly diversified, these successions can be elucidated by considering the multiple interaction variations caused by pH modulation and labor partition. First, the systematic shift of the Kp abundance from consistent decrease (Fig. 6d, g) to consistent increase (Fig. 6e, h) and divergent development (Fig. 6f, i) originated from the pH-induced variation of the interactions: At pH ≥ 7, Kp-to-Cα/Cβ inhibition was abolished but Cα/Cβ-to-Kp inhibition remained potent; at pH ≤ 6, Kp-to-Cα/Cβ inhibition became effective (Supplementary Fig. 18) but Cα/Cβ-to-Kp inhibition was significantly reduced; when there was no pH control, Cα and Cβ inhibited Kp at beginning but were later suppressed by Kp (Fig. 6a–c). Second, within a single pH setting, the final Kp abundance was lower at close Cα:Cβ partitions (e.g. 1:1) than at imbalanced (e.g., 30:1 and 1:30) because Cα and Cβ had a stronger lcnG productivity when their partitions are close (Supplementary Fig. 17). These results showed that for ecosystems containing multiple variable interactions, at least for those we tested, their succession is determined jointly by all of the variations but not by any one of them. Of note, the divergent dynamics in the absence of pH control (Fig. 6f, i) exemplified the superposition of pH and labor partition effects: At imbalanced Cα:Cβ partitions, Cα and Cβ had the potential to kill Kp at beginning but their lcnG yield was too low; later, Kp gained the lcnG resistance and further secreted lcnA to kill Cα and Cβ, leading to the monotonic increase of Kp abundance. In contrast, at the close partitions, Cα and Cβ produced significant lcnG to efficiently inhibit Kp during the initial fermentation and the lcnG remained in the culture continued to suppress Kp even though the interaction topology was later altered.

**Fig. 7:**
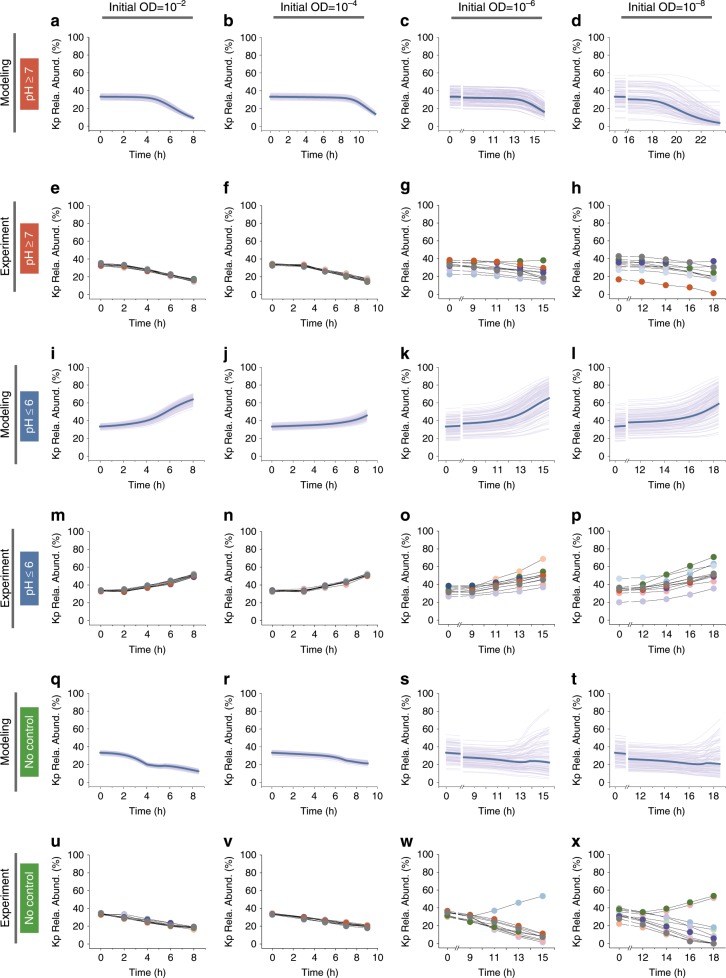
Model-predicted and experimentally measured succession of the Cα-Cβ-Kp ecosystem for varied pH conditions and initial densities. **a**–**h** Predicted (**a**–**d**) and experimentally measured (**e**–**h**) time courses of Kp abundance in the Cα-Cβ-Kp consortium when pH ≥ 7. **i**–**p** Predicted (**i**–**l**) and measured (**m**–**p**) time courses of Kp abundance in the Cα-Cβ-Kp consortium when pH ≤ 6. **q**–**x** Predicted (**q**–**t**) and measured (**u**–**x**) time courses of Kp abundance in the Cα-Cβ-Kp consortium when there is no pH control. For both model predictions and experimental measurements, the co-culture was inoculated at 1:1:1 ratio but the initial OD was varied from 10^−2^ (first column) to 10^−4^ (second column), 10^−6^ (third column) and 10^−8^ (fourth column). For each condition, a total of 100 simulation replicates and 10 experimental replicates were performed. For comparison, the deterministic dynamics of Kp without sampling-induced variability were displayed with bold lines in **a**–**d**, **i**–**l**, and **q**–**t**. Source Data available in the source data file.

To further demonstrate this finding, we conducted additional assays for the consortium by varying pH and initial total OD. Here, the model and the experimental setups were the same as previous except for the initial conditions (Methods). Accordingly, the sources of variation became pH modulation and random sampling. Our results showed that, at pH ≥ 7, the Kp abundance consistently reduced at high initial ODs (Fig. 7a–f), owing to the cooperative inhibition of Cα and Cβ to Kp; However, at low initial ODs, it could also remain largely invariant in some replicates (Fig. 7c–d, g–h) since increasing variations at random sampling abolished the cooperation (Fig. 2e–f). By contrast, in the absence of sampling-induced variations, the Kp abundance always declined regardless of initial ODs (Fig. 7a–d, bold lines). At pH ≤ 6, Kp became increasingly dominant regardless of initial ODs (Fig. 7i–l, m–p), because Kp constitutively suppressed Cα and Cβ and such a suppression was not affected by the fluctuations of Cα:Cβ ratios. These results are consistent with the ecosystem succession when sampling-induced variations were eliminated (Fig. 7i–l, bold lines). When there was no pH control, Kp declined minorly to a plateau at high initial ODs (Fig. 7q–r, u–v), attributed to the factors that Cα and Cβ collaborated to suppress Kp initially but were later suppressed by Kp due to pH reduction. At low initial ODs, it declined as the high initial OD case or diverged to be dominant (Fig. 7s–t, w–x) because increasing randomness diminished the Cα/Cβ-to-Kp inhibition at the beginning but did not affect Kp-to-Cα/Cβ suppression later. For comparison, in the absence of variation, there was no Kp dominance under all initial conditions (Fig. 7q–t, bold lines). These results demonstrated again that it is the joint regulation from multiple interaction variations that determines the dynamics of the ecosystems.

To quantitatively evaluate the capacity of the variability-incorporated modeling scheme, we calculated relative errors, defined as the differences between simulation and experimental results divided by experimental measures, for all of the simulations and experimental data above. The results (Supplementary Fig. 19) showed that, for most of the comparisons, the simulations agree quantitatively with experimental measures with the mean absolute relative errors (MARE) falling within the range of (0, 0.2) (panels a–d, g). For a subset of the cases, the models have larger relative errors but yet qualitatively agree well with the experimental findings (panels e, f, h). The encouraging agreements between the simulations and experiments demonstrated that incorporating interaction variability into ecosystem modeling is a promising strategy for quantitative and predictive understanding of complex community behaviors. Meanwhile, the discrepancies in certain cases suggest that the current models may need to consider additional processes involved in the experimental ecosystems, such as the nonlinearity observed during parameter fitting in Supplementary Fig. 8, in order to achieve a better modeling-experiment agreement.

## Discussion

Microbial interactions are often modeled invariant^20^; however, in nature, they constantly fluctuate over time and such fluctuations in strength are shown to be profound to ecosystem behaviors^17–32^. Using synthetic microbial consortia as simple and reliable platforms, we showed that increasing variations of interaction diversifies an ecosystem’s succession into distinct outcomes. We also showed that, when there are multiple variable interactions^18,19,25^, these variations collectively, but not a single one, regulate the behaviors of a community. Together, our results established interaction variability as a critical modulator of ecosystem behaviors.

Our synthetic ecosystems are relatively simple as they contain only a competition and a cooperation. Yet, their dynamics can be dramatically modulated by the variations of interaction. In native ecologies, there are significantly more cellular interactions and more microbial and environmental factors that modulate cellular interactions. We thus speculate that interaction variability is not specific to our synthetic systems and potentially a universal determinant for microbial ecosystem succession.

Searching for assembly rules has been invaluable to our understanding of community organization^65–67^. In our study, we observed the diversification of ecosystem succession with increasing variations, which illustrates the intrinsic complexity and context-dependence nature of community behaviors. This finding implies that, in certain scenarios, qualitative rules may not be sufficient to specify the assembly of a community; instead, quantitative and systematic characterizations are needed. The result might also partially explain the current difficulty in predicting microbial ecosystem behaviors, an existing key challenge in microbial ecology^4^.

Lastly, our study shows encouraging consistence between experiment and mathematical modeling, which illustrates the promise of variability-cast ecosystem modeling for bottom-up predictions of the structure and dynamics of microbial ecosystems. Together, this work provides fundamental insights into the organization of microbial communities and also the de novo engineering of microbial consortia for various biotechnological applications.

## Methods

### Strain and growth conditions

All strains are derived from *L. lactis* MG1363 and grown at 30 °C in M17 broth supplemented with 0.5% (w/v) glucose and 5 μg mL^−1^ of chloramphenicol (GM17/Cm). Tween 80 was added at a final concentration of 0.1% (v/v) when necessary. Cell cultures are adjusted with 2 M NaOH solution every hour to maintain pH above 7. To achieve pH ≤ 6, cell cultures are adjusted by 1 M HCl every hour. Strains used in this study are described in Supplementary Table 1.

### Plasmid construction

All plasmids used in this study were developed from a *L. lactis*-*E. coli* shuttle vector, pleiss-Nuc^68^, and described in Supplementary Table 1. Oligos for plasmid construction are listed in Supplementary Table 2. To generate the plasmid pleiss-lcnG for lactococcin G production, a 5-kb fragment of lcnG gene cluster including *lagA*, *lagB*, *lagC*, *lagD*, and *lagE* was amplified from the genome of *L. lactis* LMG 2081 using primers of lcnG-F and lcnG-R^53^, and subsequently assembled with a fragment of pleiss-Nuc amplified with primers P_g_-F and P_g_-R using Gibson assembly. The plasmid pleiss-lcnG was then transformed into *L. lactis* MG1363 to obtain the lcnG producing strain Cαβ. To construct lcnG subunit expression plasmids pleiss-Cα_0_ and pleiss-Cβ_0_, the α or β coding gene was deleted from the plasmid pleiss-lcnG by reverse PCR and Gibson assembly using two pairs of primers: Cα_0_-F/Cα_0_-R and Cβ_0_-F/Cβ_0_-R. The resulting plasmids were transformed into *L. lactis* MG1363 to generate strains Cα_0_ and Cβ_0_. To enable screening and counting of cells with different subunits, a gfp or rfp reporter gene, *yemGFP* or *mCherry* was introduced using primers P_cα_-F/P_cα_-R and Cα-F/Cα-R, generating the plasmids pleiss-Cα and pleiss-Cβ. These plasmids were subsequently transformed into *L. lactis* MG1363 to construct the reporter version of α and β peptide producer Cα and Cβ. As a control, plasmid pleiss-Cβ’ was generated by deleting the β precursor gene *lagB* from the plasmid pleiss-Cβ with primers P_Cβ’_-F, P_Cβ’_-F, Cβ’-F and Cβ’-R. The resulting plasmid was then transformed into *L. lactis* MG1363 to get a β-free variant Cβ’. To increase the productivity of α peptide in Cα_0_, an additional copy of expression cassette of α under the control of P_4_ promoter was inserted to pleiss-Cα_0_ using primers P_cα0-P4α_-F/ P_cα0-P4α_-R and Cα_0_-P_4a_-F/ Cα_0_-P_4a_-R^69^, generating the variant Cα_0v2_. Cβ_0v1/3_ and Cβ_0v1/5_, with approximately 1/3 and 1/5 of the productivity of Cβ_0_ respectively, were created through inserting simple short repeat sequence (AT)_n_ into the spacer region of ribosome binding site of β peptide to weaken the translational initiation rate with primers P_Cβ0v1/3_-F/ P_Cβ0v1/3_-R, Cβ_0v1/3_-F/Cβ_0v1/3_-R, P_Cβ0v1/5_-F/ P_Cβ0v1/5_-R, Cβ_0v1/5_-F/Cβ_0v1/5_-R^70^. The lactococcin A producing plasmid pleiss-lcnA was constructed by assembling the lcnA gene cluster from the plasmids pFI2396 and pFI2148 with pleiss-Nuc vector^62^. To simplify the detection of the lcnA-producing strain, a reporter gene *gusA3* was amplified from the plasmid pTRK892^63^ and then inserted into pleiss-lcnA using primers P_lcnA-gusA3_-F/P_lcnA-gusA3_-R and lcnA-gusA3-F/lcnA-gusA3-R. The resulting plasmid pleiss-lcnA-gusA3 was transformed into *L. lactis* MG1363 to generate the lcnA-producing strain Ks. Kr, as a control strain for Ks with immunity to lcnG and a gusA3 reporter, was created by assembling the immunity gene *lagC* of lcnG and *GusA3* using the primers of P_IG-GusA3_-F/P_IG-GusA3_-R and GusA3-F/GusA3-R. To create the strain with pH-dependent lcnA production and lcnG resistance, plasmid pleiss-P_774_-lcnA-gusA3 was firstly created by replacing the *lcnA*’s native promoter P_lcna_ of the plasmid pleiss-lcnA-gusA3 with a pH-inducible promoter P_774_^64^. Subsequently, pleiss-P_774_-lcnA-gusA3 was assembled with the fragment of P_774_-lagC using two pairs of primers: P_p774-lcnA_-F and P_p774-lcnA_-R, P_774_-lcnA-F and P_774_-lcnA-R; P_p774-lagC_-F and P_p774-lagC_-R, P_774_-lagC-F and P_774_-lagC-R, resulting in the final plasmid pleiss-P_774_-lcnA-P_774_-lagC-gusA3. The final plasmid was then transformed into *L. lactis* MG1363 to generate the strain Kp.

### Measurement of lcnG productivity

The agar diffusion assay was performed using a protocol adapted from a previous study^55^. Specifically, cultures of lcnG producing cells were grown in GM17/Cm/Tween broth at 30 °C overnight under corresponding culture conditions. The overnight cultures were inoculated in fresh media at 1:50 dilution and grown to the early stationary phase. Supernatants were obtained by centrifuging at 10,000× *g* for 10 min. Then, 30 μL samples were added into the wells in a double-layer agar (15 mL of GM17/Cm/Tween with 0.75% agar for each layer) in which the bottom layer was seeded with 50 μL of overnight culture of inducer strain *L. lactis* NZ9000/pleiss-Nuc. After incubation at 30 °C for 8 h, the inhibition zones were characterized by the blank circles around wells. To establish a standard curve of relative lcnG concentration for quantitatively evaluating samples, the concentration of lcnG in the supernatant of Cαβ culture at the early stationary phase is defined as 100%. And then the cell-free supernatant of Cαβ culture was diluted with fresh GM17/Cm/Tween media to the relative lcnG concentrations of 90%, 80%, 70%, 60%, 50%, 40%, 30%, 20%, 10%, 5%, 2%, and 1%. Next, 30 μL of samples were added into the wells in the top layer agar. After incubation at 30 °C for 8 h, the inhibition zones emerged and a standard curve was drawn by measuring the diameters of inhibition zones produced by different relative concentrations of lcnG. Using this curve, the relative concentrations of lcnG from tested samples were estimated.

### Characterization of variations from labor partition

Monocultures of Cα and Cβ (Cα_0_ and Cβ_0_) were inoculated in GM17/Cm/Tween liquid medium at 30 °C overnight. Then the optical density at 600 nm (OD) of monocultures were measured and the co-cultures of Cα and Cβ (Cα_0_ and Cβ_0_) were mixed at a variety of ratios (30:1, 10:1, 3:1, 1:1, 1:3, 1:10, 1:30) with a start total OD_600_ of 10^−2^. After 8 h, the co-culture cells were centrifuged at 10,000× *g* for 10 min to obtain the co-culture supernatants. To test the cooperative strength in supernatant mixtures, overnight monocultures were firstly transformed to fresh GM17/Cm/Tween broth at the initial OD_600_ of 10^−2^ individually. Then supernatants were extracted after growth for 8 h by centrifuging and mixed at different ratios as mentioned above. Finally, 30 μL of samples were used to determine the activity of lcnG by agar diffusion assays to examine the variability of cooperation in co-culture supernatants and monoculture supernatant mixtures. Additionally, to evaluate the role of subunit stoichiometric ratio in cooperation variation, monoculture supernatants were also prepared from Cα_0_, Cα_0v2_, Cβ_0v1/3_, and Cβ_0v1/5_ as described above and mixed at ratios from 30:1, 10:1, 3:1, 1:1, 1:3, 1:5, 1:6, 1:10 to 1:30. Among them, ratios of 1:5 and 1:6 were used for determining the optimal cooperativity between Cα_0_ and Cβ_0v1/5_ (Cα_0v2_ and Cβ_0v1/3_). Then, activities of lcnG in these combinations were determined by agar diffusion assay. Similarly, to measure the cooperative inhibition from Cα and Cβ to Ks (Kp), three steps were involved. First, starting media were prepared by mixing 2-fold concentrated GM17/Cm/Tween medium with equal volume of supernatants from Cα-Cβ co-culture at the early stationary phase that were filtered with sterile 0.22 μm filter and adjusted to necessary pH. Second, overnight Ks (Kp) monoculture was inoculated with an initial OD of 10^−2^ to the starting media. Third, the relative abundances of Ks were measured with fluorescence microscope from the starting to 8 h of incubation, during which pH was controlled properly if needed. For comparison, starting media were also prepared by mixing 2-fold concentrated GM17/Cm/Tween medium with equal volume of supernatants of Cα-Cβ’ co-culture for culturing and measuring Ks (Kp). Each experimental condition has three replicates (*n* = 3).

### Determination of the maximum dilution rate

To determine the minimum OD_600_ that is required for cell growth in a fresh medium, monocultures of Cα_0_, Cβ_0_, Cα, Cβ and Ks were serially diluted to an OD_600_ of 10^−2^ to 10^−13^ at 1:10 dilution and the survival rate of each OD_600_ was calculated by counting the growing cultures in twenty replicates of each OD_600_. After incubation at 30 °C for 24 h, the tubes with cell growth were counted to calculate the survival rate of different initial OD_600_. Our experiment (Supplementary Fig. 5) showed that 10^−8^ is the minimum OD_600_ with a 100% survival rate for almost all strains. An inoculation with an initial OD_600_ lower than 10^−8^ could result in failure in growth and would disturb the studies of small number fluctuations in the community. Therefore, the initial OD_600_ after dilution in the serial dilution experiments should be higher than 10^−8^.

### Characterization of cooperation variations from sampling

To examine the effect of sampling on the variability of Cα-Cβ cooperation, overnight cultures of Cα and Cβ were washed twice with sterile PBS buffer (pH = 7) and re-suspended in PBS buffer. Then, the Cα and Cβ suspensions were adjusted to an OD_600_ of 1.0 with PBS buffer, and mixed together at 1:1 ratio. The resulting suspension was used as a start culture and diluted to the OD_600_ of 10^−2^, 10^−4^, 10^−6^ and 10^−8^ through serial 1:10 dilutions with PBS buffer. The total volume of a start culture was set at 5 mL. After being prepared, all samples were centrifuged at 10,000× *g* for 15 min to remove supernatants, and 5 mL of fresh GM17/Cm media were subsequently added for cell growth. At the end of incubation, the supernatants were obtained. Subsequently, inhibition zone assays were conducted to determine the strength of cooperation. As cultures with different initial ODs require different incubation times, for each initial condition we chose sampling time based on the corresponding growth profile so that the ODs at each time point are comparable across the samples (Supplementary Fig. 12). Such samplings enable a consistent and proper comparison. Ten trials were performed for each initial condition.

### Three-strain cooperator-varying experiments

The initial total OD_600_ of three strains Cα, Cβ and Ks (Kr or Kp) was set at 10^−2^. The start abundance of Ks (Kr or Kp) was fixed at 33.3% in the population but the ratios of Cα and Cβ were set at 30:1, 10:1, 3:1, 1:1, 1:3, 1:10, and 1:30. During incubation, samples were taken every two hours for measuring their ODs and the relative numbers of green (Cα), red (Cβ), and non-fluorescent cells were counted under an AMG EVOS FL fluorescence microscope using green, red and bright field channels. Notably, for the pH-controlled three-strain experiments, the overnight culture of Kp was washed twice with sterile PBS buffer (pH = 7) and then inoculated with an OD of 10^−2^ into fresh medium with proper different pH controls (pH ≥ 7, pH ≤ 6 and no pH control). During the course of fermentation, culture samples were collected every hour to determine environmental pH using pH meter and Kp’s lcnA productivity using the inhibition zone assay. Each culture condition has three replicates (*n* = 3). The pH and inhibition zones were calculated as mean ± s.d.

### Three-strain sampling experiments

For the three-strain ecosystem of Cα, Cβ, and Ks (Kp), Cα and Cβ PBS suspensions were individually adjusted to OD_600_ of 1 and then mixed at 1:1 ratio; Ks (Kp) PBS suspension was also adjusted to OD_600_ of 1. Subsequently, both the Cα-Cβ mixture and Ks (Kp) were diluted to the OD_600_ of 10^−2^, 10^−4^, 10^−6^, and 10^−8^ with PBS buffer in 1:10 dilution fold. At each concentration, the Cα-Cβ mixture and Ks (Kp) monoculture suspensions were mixed at a 2:1 ratio to reach a final ratio of 1:1:1 for Cα, Cβ and Ks (Kp). The total volumes of both monoculture and co-culture suspensions were set at 5 mL. The prepared samples were centrifuged at 10,000× *g* for 15 min to remove supernatants, and 5 mL of fresh GM17/Cm media were subsequently added for cell growth. The co-cultures were then incubated at 30 °C and propagated for 8 (10^−2^), 9 (10^−4^), 14 (10^−6^) and 20 (10^−8^) hours which was needed for entering the early stationary phase. To consistently determine cell numbers in different initial ODs (e.g., 10^−2^, 10^−4^, 10^−6^, 10^−8^), the initial relative abundances of Cα, Cβ and Ks (Kp) were measured by colony forming unit (CFU) counting instead of flow cytometry, because flow cytometry typically requires a minimum cell number (10^4^ cells per mL) in samples. For the samples with an initial OD_600_ of 10^−2^ or 10^−4^, the mixed suspensions were diluted to around OD_600_ of 5×10^−6^ with sterile PBS buffer and 1 mL of the diluted suspension was plated on the GM17/Cm plate supplemented with X-Gluc for counting Ks (Kp). For the samples with an initial OD_600_ of 10^−6^, 1 mL of the mixed suspensions were directly plated without dilution on the GM17/Cm plate supplemented with X-Gluc. For the samples with an initial OD_600_ of 10^−8^, 5 mL of the mixed suspensions were plated without dilution on the GM17/Cm plate supplemented with X-Gluc. During the period of incubation, the samples were taken to measure OD_600_ and the fraction of Ks (Kp) strain was calculated by counting the cells under microscope. For the ecosystem of Cα, Cβ and Kp, the above procedure remained the same except that the diluted co-culture samples were inoculated into pH-defined media whose pH was adjusted every hour. Ten trials were performed for each initial condition.

### Quantification of lcnA inhibition

To measure the inhibition from Ks to Cα or Cβ, overnight Ks and Cα or Cβ monoculture were inoculated into fresh GM17/Cm/Tween media at 1:1 ratio based on OD_600_. Then, the relative abundances of Ks were measured from the starting to 8 h of incubation with fluorescence microscope. The similar procedure was also used to determine the inhibition from Kp to Cα and Cβ in pH-defined media (pH ≥ 7, pH ≤ 6 and no pH control). Each culture condition has three replicates (*n* = 3).

### GusA3 enzyme assay

To directly visualize the Ks in three-strain system, GusA3 protein was used as a reporter to produce a blue green color. At the end of growth in three-strain consortium, a final concentration of 2 mM of 5-bromo-4-chloro-3-indolyl- β-D-glucuronide (X-Gluc) was added into the cultures and the blue green color formation was monitored. After incubation at 37 °C for 1 h, the supernatants were obtained by centrifuging the cultures at 10,000× *g* for 10 min and images were taken.

### Mathematical modeling

In concert with the experimental ecosystem assembly, a bottom-up strategy was utilized to construct the dynamic models of the synthetic ecosystems. Briefly, we first created and characterized growth models of monocultures (Cα, Cβ, Ks, Kr, Kp), then models for the cooperative Cα-Cβ species as well as their interactions, and finally assembled individual modules into integrated models that represent complete ecosystems (e.g., Cα-Cβ-Ks, Cα-Cβ-Kr and Cα-Cβ-Kp consortia). During model construction, ordinary differential equations were used to quantitatively describe the kinetics of three major classes of variables: nutrient availability, cell populations and bacteriocins mediating cellular interactions. Parameters were determined using data in the literature or by fitting the models to our experiments. MATLAB software was used to simulate the models, produce plots, and fit data for the models. A detailed description of the models is available in Supplementary Information.

### Statistical analysis

All of the experiments were performed for multiple times. Replicate numbers of the experiments (*n*) are indicated in the figure legends. Sample sizes were chosen based on standard experimental requirement in molecular biology. Data are presented as mean ± s.d.

### Reporting summary

Further information on research design is available in the Nature Research Reporting Summary linked to this article.

## Supplementary information

Supplementary Information

Reporting Summary

## Data Availability

Strains and plasmids constructed in this study are available from the corresponding author upon request. Data supporting the results in this paper are available within the paper and its supplementary information files. The source data of Figs. 2b,c,e, 3c,d,g,h, 4a-h, 5b-d, 6d-6i, 7 and Supplementary Figs. 1a, 2c, 3, 5, 7b and 8-19 are provided as a Source Data file. All other relevant data are available from the author upon reasonable request.

## Source data

Source Data File

## Code availability

Custom MATLAB codes developed in this study are available from the corresponding author upon request.

## References

[1] Falkowski PG, Fenchel T, Delong EF (2008). The microbial engines that drive Earth’s biogeochemical cycles. Science.

[2] Berendsen RL, Pieterse CM, Bakker PA (2012). The rhizosphere microbiome and plant health. Trends Plant Sci..

[3] Cho I, Blaser MJ (2012). The human microbiome: at the interface of health and disease. Nat. Rev. Genet..

[4] Widder S (2016). Challenges in microbial ecology: building predictive understanding of community function and dynamics. ISME J..

[5] Brenner K, You L, Arnold FH (2008). Engineering microbial consortia: a new frontier in synthetic biology. Trends Biotechnol..

[6] Johns NI, Blazejewski T, Gomes AL, Wang HH (2016). Principles for designing synthetic microbial communities. Curr. Opin. Microbiol..

[7] Großkopf T, Soyer OS (2014). Synthetic microbial communities. Curr. Opin. Microbiol..

[8] Mimee M, Citorik RJ, Lu TK (2016). Microbiome therapeutics - advances and challenges. Adv. Drug Deliv. Rev..

[9] Song HS, Cannon WR, Beliaev AS, Konopka A (2014). Mathematical modeling of microbial community dynamics: a methodological review. Processes.

[10] Cordero OX, Datta MS (2016). Microbial interactions and community assembly at microscales. Curr. Opin. Microbiol..

[11] Gonze D, Coyte KZ, Lahti L, Faust K (2018). Microbial communities as dynamical systems. Curr. Opin. Microbiol..

[12] Zomorrodi AR, Segrè D (2016). Synthetic ecology of microbes: mathematical models and applications. J. Mol. Biol..

[13] Faust K, Raes J (2012). Microbial interactions: from networks to models. Nat. Rev. Microbiol..

[14] Mounier J (2008). Microbial interactions within a cheese microbial community. Appl. Environ. Microbiol..

[15] Marino S, Baxter NT, Huffnagle GB, Petrosino JF, Schloss PD (2014). Mathematical modeling of primary succession of murine intestinal microbiota. Proc. Natl. Acad. Sci. USA.

[16] Stein RR (2013). Ecological modeling from time-series inference: insight into dynamics and stability of intestinal microbiota. PLoS Comput. Biol..

[17] Garcia FC, Bestion E, Warfield R, Yvon-Durocher G (2018). Changes in temperature alter the relationship between biodiversity and ecosystem functioning. Proc. Natl Acad. Sci. USA.

[18] Ratzke C, Gore J (2018). Modifying and reacting to the environmental pH can drive bacterial interactions. PLoS Biol..

[19] Rivett DW (2016). Resource-dependent attenuation of species interactions during bacterial succession. ISME J..

[20] Hart SFM (2019). Uncovering and resolving challenges of quantitative modeling in a simplified community of interacting cells. PLoS Biol..

[21] LaSarre B, McCully AL, Lennon JT, McKinlay JB (2017). Microbial mutualism dynamics governed by dose-dependent toxicity of cross-fed nutrients. ISME J..

[22] Bachmann H, Molenaar D, Kleerebezem M, van Hylckama Vlieg JE (2011). High local substrate availability stabilizes a cooperative trait. ISME J..

[23] Liu J, Wu C, Huang IH, Merritt J, Qi F (2011). Differential response of *Streptococcus mutans* towards friend and foe in mixed-species cultures. Microbiol-SGM.

[24] Nguyen AT, Jones JW, Ruge MA, Kane MA, Oglesby-Sherrouse AG (2015). Iron depletion enhances production of antimicrobials by *Pseudomonas aeruginosa*. J. Bacteriol..

[25] Kreth J, Merritt J, Shi W, Qi F (2005). Competition and coexistence between *Streptococcus mutans* and *Streptococcus sanguinis* in the dental biofilm. J. Bacteriol..

[26] Liu F, Mao J, Lu T, Hua Q (2019). A synthetic, context-dependent microbial consortium of predator and prey. ACS Synth. Biol..

[27] Andrade-Domínguez A, Salazar E, Vargas-Lagunas M, Kolter R, Encarnación S (2014). Eco-evolutionary feedbacks drive species interactions. ISME J..

[28] Buffie CG (2014). Precision microbiome reconstitution restores bile acid mediated resistance to *Clostridium difficile*. Nature.

[29] Palmer AC, Angelino E, Kishony R (2010). Chemical decay of an antibiotic inverts selection for resistance. Nat. Chem. Biol..

[30] van der Putten WH, Klironomos JN, Wardle DA (2007). Microbial ecology of biological invasions. ISME J..

[31] Kuipers OP, Beerthuyzen MM, de Ruyter PG, Luesink EJ, de Vos WM (1995). Autoregulation of nisin biosynthesis in *Lactococcus lactis* by signal transduction. J. Biol. Chem..

[32] Coburn PS, Pillar CM, Jett BD, Haas W, Gilmore MS (2004). *Enterococcus faecalis* senses target cells and in response expresses cytolysin. Science.

[33] Kaern M, Elston TC, Blake WJ, Collins JJ (2005). Stochasticity in gene expression: from theories to phenotypes. Nat. Rev. Genet..

[34] Elowitz MB, Levine AJ, Siggia ED, Swain PS (2002). Stochastic gene expression in a single cell. Science.

[35] Kiviet DJ (2014). Stochasticity of metabolism and growth at the single-cell level. Nature.

[36] Ramkrishna D, Mahoney AW (2002). Population balance modeling. Promise for the future. Chem. Eng. Sci..

[37] Shu CC, Chatterjee A, Dunny G, Hu WS, Ramkrishna D (2011). Bistability versus bimodal distributions in gene regulatory processes from population balance. PLoS Comput. Biol..

[38] Hellweger FL, Bucci V (2009). A bunch of tiny individuals—Individual-based modeling for microbes. Ecol. Model.

[39] Klitgord N, Segre D (2010). Environments that induce synthetic microbial ecosystems. PLoS Comput. Biol..

[40] Resat H, Bailey V, McCue LA, Konopka A (2012). Modeling microbial dynamics in heterogeneous environments: Growth on soil carbon sources. Microb. Ecol..

[41] Shou W, Ram S, Vilar JM (2007). Synthetic cooperation in engineered yeast populations. Proc. Natl. Acad. Sci. USA.

[42] Gore J, Youk H, van Oudenaarden A (2009). Snowdrift game dynamics and facultative cheating in yeast. Nature.

[43] Balagaddé FK (2008). A synthetic *Escherichia coli* predator-prey ecosystem. Mol. Syst. Biol..

[44] Ozgen VC, Kong W, Blanchard AE, Liu F, Lu T (2018). Spatial interference scale as a determinant of microbial range expansion. Sci. Adv..

[45] Mee MT, Collins JJ, Church GM, Wang HH (2014). Syntrophic exchange in synthetic microbial communities. Proc. Natl. Acad. Sci. USA.

[46] Kerr B, Riley MA, Feldman MW, Bohannan BJ (2002). Local dispersal promotes biodiversity in a real-life game of rock-paper-scissors. Nature.

[47] Chuang JS, Rivoire O, Leibler S (2009). Simpson’s paradox in a synthetic microbial system. Science.

[48] Wintermute EH, Silver PA (2010). Emergent cooperation in microbial metabolism. Mol. Syst. Biol..

[49] Youk H, Lim WA (2014). Secreting and sensing the same molecule allows cells to achieve versatile social behaviors. Science.

[50] West SA, Griffin AS, Gardner A, Diggle SP (2006). Social evolution theory for microorganisms. Nat. Rev. Microbiol..

[51] Wingreen NS, Levin SA (2006). Cooperation among microorganisms. PLoS Biol..

[52] Celiker H, Gore J (2013). Cellular cooperation: insights from microbes. Trends Cell Biol..

[53] Mirkovic N (2016). *Lactococcus lactis* LMG2081 produces two bacteriocins, a non lantibiotic and a novel lantibiotic. Appl. Environ. Micobiol.

[54] Moll G (1996). Lactococcin G is a potassium ion-conducting, two-component bacteriocin. J. Bacteriol..

[55] Kong W, Kapuganti VS, Lu T (2016). A gene network engineering platform for lactic acid bacteria. Nucleic Acids Res..

[56] Bentley WE, Mirjalili N, Andersen DC, Davis RH, Kompala DS (1990). Plasmid-encoded protein: The principal factor in the “metabolic burden” associated with recombinant bacteria. Biotechnol. Bioeng..

[57] Glick BR (1995). Metabolic load and heterologous gene expression. Biotechnol. Adv..

[58] Lidstrom ME, Konopka MC (2010). The role of physiological heterogeneity in microbial population behavior. Nat. Chem. Biol..

[59] Stegen JC, Lin XJ, Konopka AE, Fredrickson JK (2012). Stochastic and deterministic assembly processes in subsurface microbial communities. ISME J..

[60] Masel J (2011). Genetic drift. Curr. Biol..

[61] Hallatschek O, Hersen P, Ramanathan S, Nelson DR (2007). Genetic drift at expanding frontiers promotes gene segregation. Proc. Natl Acad. Sci. USA.

[62] Fernández A, Horn N, Gasson MJ, Dodd HM, Rodríguez JM (2004). High-level coproduction of the bacteriocins nisin A and lactococcin A by *Lactococcus lactis*. J. Dairy Res..

[63] Callanan MJ, Russell WM, Klaenhammer TR (2007). Modification of Lactobacillus β-glucuronidase activity by random mutagenesis. Gene.

[64] Madsen SM, Arnau J, Vrang A, Givskov M, Israelsen H (1999). Molecular characterization of the pH–inducible and growth phase–dependent promoter P170 of *Lactococcus lactis*. Mol. Microbiol..

[65] Cody ML, Diamond JM (1976). Ecology and evolution of communities. Nature.

[66] Gotelli NJ (1999). How do communities come together?. Science.

[67] Weiher, E. & Keddy, P. Ecological assembly rules: perspectives, advances, retreats. Cambridge University Press (2001).

[68] Le Loir Y, Gruss A, Ehrlich SD, Langella P (1998). A nine-residue synthetic propeptide enhances secretion efficiency of heterologous proteins in *Lactococcus lactis*. J. Bacteriol..

[69] Zhu D (2015). Isolation of strong constitutive promoters from *Lactococcus lactis* subsp. lactis N8. FEMS Microbiol. Lett..

[70] Egbert RG, Klavins E (2012). Fine-tuning gene networks using simple sequence repeats. Proc. Natl. Acad. Sci. USA.

