# Supplementary material for: Interaction variability shapes succession of synthetic microbial ecosystems
Source: Nat Commun. 2020 Jan 16;11:309. doi: 10.1038/s41467-019-13986-6 (PMC6965111; doi:10.1038/s41467-019-13986-6)
Supplement: Supplementary file 1 — Supplementary Information [file 41467_2019_13986_MOESM1_ESM.pdf]

Supplementary Information

Interaction Variability Shapes Succession of Synthetic Microbial Ecosystems

Feng Liu, Junwen Mao, Wentao Kong, Qiang Hua,
Youjun Feng, Rashid Bashir and Ting Lu

Correspondence and requests for materials should be addressed to T.L. (luting@illinois.edu).

Contents

1 Supplementary Notes **3**
1.1 Modeling the Cα-Cβ-Ks ecosystem 3
1.1.1 Cα, Cβ and Ks monoculture growth 3
1.1.2 Growth and lcnG production of the Cα-Cβ co-culture 3
1.1.3 Interactions between Ks and the Cα-Cβ pair 6
1.1.4 Predictions for the Cα-Cβ-Ks ecosystem 7
1.2 Modeling the Cα-Cβ-Kr ecosystem 9
1.2.1 Monoculture Kr growth 9
1.2.2 Modeling and predictions of the Cα-Cβ-Kr ecosystem 9
1.3 Modeling the Cα-Cβ-Kp ecosystem 10
1.3.1 Kp monoculture growth 10
1.3.2 Growth and lcnG production of the Cα-Cβ co-culture in pH-controlled settings 10
1.3.3 Interactions between Kp and the Cα-Cβ pair 11
1.3.4 Predictions for the Cα-Cβ-Kp ecosystem 13

2 Supplementary Figures **15**

3 Supplementary Tables **34**

4 Supplementary References **59**

1 Supplementary Notes

In concert with our assembly of experimental ecosystems, we developed dynamic community models in a bottom-up fashion to capture and predict the behaviors of synthetic consortia. Briefly, we created a set of ordinary differential equations (ODE) models to describe the kinetics of the three classes of variables: nutrient availability, cell populations, and bacteriocins mediating cellular interactions. A detailed description of the models is provided below.

1.1 Modeling the Cα-Cβ-Ks ecosystem

1.1.1 Cα, Cβ and Ks monoculture growth

We used Monod equations[1] to describe the growth of single *Lactococcus lactis* strains (Cα, Cβ and Ks) in batch culture. The equations are as follows:

$$\begin{aligned} \frac{dN}{dt} &= -\frac{1}{\gamma_{C_\alpha}} \frac{g_{C_\alpha} N}{N_{C_\alpha} + N} C_\alpha \\ \frac{dC_\alpha}{dt} &= \frac{g_{C_\alpha} N}{N_{C_\alpha} + N} C_\alpha, \end{aligned} \tag{1}$$

$$\begin{aligned} \frac{dN}{dt} &= -\frac{1}{\gamma_{C_\beta}} \frac{g_{C_\beta} N}{N_{C_\beta} + N} C_\beta \\ \frac{dC_\beta}{dt} &= \frac{g_{C_\beta} N}{N_{C_\beta} + N} C_\beta, \end{aligned} \tag{2}$$

$$\begin{aligned} \frac{dN}{dt} &= -\frac{1}{\gamma_{Ks}} \frac{g_{Ks} N}{N_{Ks} + N} K_s \\ \frac{dK_s}{dt} &= \frac{g_{Ks} N}{N_{Ks} + N} K_s, \end{aligned} \tag{3}$$

where N is the nutrient level, C_α, C_β and K_s are the population densities of the strain Cα, Cβ and Ks respectively, g_{C_α}, g_{C_β} and g_{Ks} are the corresponding maximum growth rates, and N_{C_α}, N_{C_β} and N_{Ks} are the corresponding half-saturation constants. The growth yield coefficients γ_{C_α}, γ_{C_β} and γ_{Ks} are the ratios of biomass of each strain to the mass of required nutrient. By fitting Supplementary Equations 1-3 to the experimental data in Supplementary Figures 3a and 9a, we obtained the growth parameters for the three strains (Supplementary Table 5).

1.1.2 Growth and lcnG production of the Cα-Cβ co-culture

To capture the Cα-Cβ co-culture, a model needs to involve the growth of individual strains, nutrient availability in the co-culture as well as their production of lcnG subunits and active lcnG. The specific equations of the system can be written as

$$\begin{aligned}
\frac{dN}{dt} &= -\frac{1}{\gamma_{C_\alpha}}\frac{g_{C_\alpha}N}{N_{C_\alpha}+N}C_\alpha - \frac{1}{\gamma_{C_\beta}}\frac{g_{C_\alpha}N}{N_{C_\beta}+N}C_\beta \\
\frac{dC_\alpha}{dt} &= g_{C_\alpha}\frac{N}{N_{C_\alpha}+N}C_\alpha \\
\frac{dC_\beta}{dt} &= g_{C_\beta}\frac{N}{N_{C_\beta}+N}C_\beta \\
\frac{d\alpha}{dt} &= p_\alpha C_\alpha - k_{\alpha\beta}\alpha\beta \\
\frac{d\beta}{dt} &= p_\beta C_\beta - k_{\alpha\beta}\alpha\beta \\
\frac{dG}{dt} &= k_{\alpha\beta}\alpha\beta - d_G G
\end{aligned} \tag{4}$$

where N is the common nutrient of the medium, C_α and C_β are the population densities of the strain Cα and Cβ, α and β are the concentrations of lcnG's two subunits, peptide α and β, released from Cα and Cβ respectively, G is the concentration of mature active lcnG. Similar to the monoculture model, g_{C_α} and g_{C_β} are the maximum growth rates, and N_{C_α} and N_{C_β} are the corresponding half-saturation constants, the growth yield coefficients γ_{C_α} and γ_{C_β} are the ratios of biomass of each strain to the mass of required nutrient. In addition, parameters are introduced to describe lcnG production: (1) p_α and p_β are the production rate of peptide α and β, (2) $k_{\alpha\beta}$ is the dimerization rate of the peptides α and β, and (3) d_G is the degradation rate of lcnG. For simplicity, we assume $p_\alpha = p_\beta$, i.e., strains Cα and Cβ have a comparable rate of peptide production.

LcnG productivity under varied labor partitions. In our study, we quantified lcnG productivity by normalizing the diameters of the inhibition zones of the sample to those by the supernatant of C$\alpha\beta$ monoculture. Owing to the quantitative relationship between normalized lcnG productivity and the diameter of inhibition zones (Supplementary Figure 1), the lcnG production can be experimentally determined by measuring the size of inhibition zones formed by samples. To calculate the variation of lcnG productivity caused by labor partition, the model Equation 4 was used to conduct simulations under different initial conditions, namely, a total OD of 0.01 but various ratios of Cα:Cβ (i.e. 30:1, 10:1, 3:1, 1:1 , 1:3, 1:10 and 1:30). The dynamics of normalized lcnG productivity are shown in Supplementary Figure 8 (red solid lines) with corresponding parameters listed in Supplementary Table 6.

For Supplementary Figure 8, we noticed that, at imbalanced cases (panel **a**, **b**, and **g**), experimental results (blue bars) are consistently smaller than simulations (red lines). By contrast, in the ideally balanced case (panel **e**. Of note, 1:3 is the optimal initial Cα:Cβ ratio due to different metabolic loads), experimental results are larger than simulations. This observation implies that there is a possible nonlinear dependence on the initial ratio in the experiment, which might arise from one or both of the following reasons: (i) The formation of effective lcnG might be synergistic, although we assumed in our model that the rate of lcnG formation is proportional to the concentrations of α and β subunits. (ii) In our experiment, the normalized lcnG level was determined by quantifying the diameters

of inhibition zones formed by the lcnG-sensitive indicator cells. However, the sensitivity of the indicator might be nonlinearly proportional to the lcnG concentration, because even sensitive cells are resistant to minimal levels of lcnG, lcnG may degrade over the time course of inhibition measurement, and the size of inhibition zone is nonlinearly proportional to the lcnG level.

LcnG productivity in serial dilution. To experimentally investigate the cooperative variability arising from random sampling, equal amounts of overnight Cα and Cβ monocultures were mixed at 1:1 ratio and adjusted to a start OD_{600} of 1.0. Then the sample was diluted gradually to an OD_{600} of 10^{-2}, 10^{-4}, 10^{-6} and 10^{-8} to generate a set of starting cultures (Figure 2e-f in the main text). Subsequently, co-culture growth and lcnG productivity were measured. To mathematically capture the variation, we computationally conducted serial dilution to mimic the experimental procedures. (1) After the i-th ($i = 1, 2, 3, 4, 5, 6, 7, 8$) dilution, draw the total cell number n_i from the distribution with the mean of $\mu_i = n_{i-1}/10$ and the variance of σ_i^2 (Supplementary Table 7). (2) Determine the random fraction of Cα, f_i (Supplementary Table 8), from a Gaussian random number with the mean μ and the variance σ_i^2. (3) Specify the populations of Cα ($= n_i f_i$) and Cβ ($= n_i(1 - f_i)$, accordingly) in the sample. (4) Convert the populations into OD to obtain the concentration of Cα and Cβ after the i-th dilution. (5) Repeat Steps 1-4 to obtain 100 replicates for each of the $OD_{600} = 10^{-2}, 10^{-4}, 10^{-6}$, and 10^{-8}.

Notably, in this procedure, a few assumptions were made: (1) the initial Cα:Cβ ratio is exactly 1:1 ratio for total initial OD of 1.0; (2) a 1.0 OD_{600} of *L. lactis* corresponds to 2×10^8 cells mL^{-1} for both mono- and co-culture (Of note, for the same OD, *L. lactis* population is different from *E. coli* population); (3) The total cell number of Cα-Cβ co-culture in each dilution is drawn from a Gaussian distribution. The variance ${\sigma_i}^2$ is chosen by using the coefficient of variation ($CV_i = \sigma_i/\mu_i$) from experimental measures (Supplementary Table 7); (4) As *L. lactis* usually grow into short chains, sampling at the extremely low concentration may significantly deviate from the initial 1:1 and produce a nonlinear reduction of Cα-Cβ diversity[2,3] in a single experiment. Nevertheless, the average ratio of Cα:Cβ is kept at 1:1 as observed in our repeated experiments. (5) For simplicity, identical parameter sets are used for consecutive two dilutions. For instance, the parameters for OD$= 10^{-1}$ and OD$= 10^{-2}$ are the same, and those for OD$= 10^{-3}$ and OD$= 10^{-4}$ are also the same. With the resulting diluted initial cell densities, cell growth and lcnG production were simulated using Supplementary Equation 4 with the corresponding statistical results derived through data fitting (Supplementary Tables 7 and 8).

1.1.3 Interactions between Ks and the Cα-Cβ pair

LcnA production by Ks. We constructed a model of Ks monoculture that constitutively produces the bacteriocin lactoccoccin A (lcnA) as

$$\begin{aligned} \frac{dN}{dt} &= -\frac{1}{\gamma_{Ks}} \frac{g_{Ks}N}{N_{Ks}+N} K_s \\ \frac{dK_s}{dt} &= g_{Ks} \frac{N}{N_{Ks}+N} K_s \\ \frac{dA}{dt} &= p_A K_s - d_A A \end{aligned} \tag{5}$$

where N, K_s and A refer to the nutrient level, Ks population density and the lcnA concentration in the culture. g_{Ks} is the maximum growth rate of Ks, N_{Ks} is the corresponding half-saturation constant and γ_{Ks} is Ks' growth yield coefficient, p_A is the lcnA production rate, and d_A is the lcnA degradation rate. The fitting parameters of lcnA production are summarized in Supplementary Table 6.

LcnA-mediated Ks-to-Cα/Cβ inhibition. To characterize the inhibition, the model of Ks and Cα (or Cβ) co-culture can be written as

$$\begin{aligned} \frac{dN}{dt} &= -\frac{1}{\gamma_{C_\alpha}} \frac{g_{C_\alpha}N}{N_{C_\alpha}+N} C_\alpha - \frac{1}{\gamma_{Ks}} \frac{g_{Ks}N}{N_{Ks}+N} K_s \\ \frac{dC_\alpha}{dt} &= g_{C_\alpha} \frac{N}{N_{C_\alpha}+N} C_\alpha - I_{C_\alpha,A} \frac{A}{K_{C_\alpha,A}+A} C_\alpha \\ \frac{dK_s}{dt} &= g_{Ks} \frac{N}{N_{Ks}+N} K_s \\ \frac{dA}{dt} &= p_A K_s - d_A A \end{aligned} \tag{6}$$

where N, C_α K_s and A refer to the nutrient level, Cα population density, Ks population density and the lcnA concentration in the culture. g_{C_α} and g_{Ks} are Cα and Ks maximum growth rates respectively, N_{Ks} and N_{C_α} are the corresponding half-saturation constants, and γ_{C_α} and γ_{Ks} are the strains' growth yield coefficients, p_A is the lcnA production rate, and d_A is the lcnA degradation rate. In addition, we modeled the lcnA inhibition on Cα as a Hill function where $I_{C_\alpha,A}$ is the maximal inhibition strength, and $K_{C_\alpha,A}$ is the half killing parameter. Similarly, lcnA-mediated inhibition from Ks to Cβ can be modeled in the same fashion. The model was used to fit with the experimental data in Supplementary Figure 9c-d with the parameters summarized in Supplementary Table 6.

lcnG-mediated Cα-Cβ pair-to-Ks inhibition. To determine the inhibition of lcnG on Ks, we cultured Ks in fresh medium mixed at 1:1 ratio with the supernatant of Cα-Cβ co-culture (Supplementary Figure 9e). The corresponding Ks growth model can be expressed

as

$$\begin{aligned}
\frac{dN}{dt} &= -\frac{1}{\gamma_{Ks}} \frac{g_{Ks} N}{N_{Ks} + N} K_s \\
\frac{dK_s}{dt} &= g_{Ks} \frac{N}{N_{Ks} + N} K_s - I_{Ks,G} \frac{G}{K_{Ks,G} + G} K_s \\
\frac{dG}{dt} &= -d_G G
\end{aligned} \tag{7}$$

where N, K_s and G are the nutrient level, Ks population density and the lcnG level in the culture. g_{Ks}, γ_{Ks} and N_{Ks} are the growth parameters of Ks, d_G is the natural degradation constant. The lcnG-mediated inhibition was modeled as a Hill function where $I_{Ks,G}$ is the maximal inhibition strength and $K_{Ks,G}$ is the half killing parameter of Ks. Of note, as Ks grows in fresh medium mixed with the supernatant of Cα-Cβ co-culture, its growth parameters are different from those of pure fresh media.

For comparison, we also cultured Ks in fresh GM17 medium mixed 1:1 with the supernatant of Cα-Cβ' co-culture, where Cβ' is a variant of Cβ which is deficient in peptide β production. Thus, no active lcnG is present in the supernatant of the Cα-Cβ' co-culture. Accordingly, the dynamics of Ks shall be revised as

$$\begin{aligned}
\frac{dN}{dt} &= -\frac{1}{\gamma_{Ks}} \frac{g_{Ks} N}{N_{Ks} + N} K_s \\
\frac{dK_s}{dt} &= g_{Ks} \frac{N}{N_{Ks} + N} K_s
\end{aligned} \tag{8}$$

where the inhibition term is removed.

Our experiments showed that there is a population density decline of Ks in the first 1.5 hours when growing fresh GM17 media mixed with the supernatants of Cα-Cβ or Cα-Cβ' co-cultures (Supplementary Figure 9e, f), which is likely due to the combination of lag phase and toxicity of the supernatants. Accordingly, in our models, we incorporated this decline phase and, used the results of t$\geq$1.5 h to obtain the growth parameters (Supplementary Tables 9 and 10). Subsequently, parameters of the inhibition strength ($I_{Ks,G}$, $K_{Ks,G}$) were determined and listed in Supplementary Table 6. The Ks' relative growth rates arising from both Ks growth and the inhibition from lcnG, in the GM17 medium mixed with the supernatants of Cα-Cβ co-culture and with the Cα-Cβ' co-culture, characterized the inhibition strength of lcnG on Ks (the rightmost bar in Figure 5d in the main text).

1.1.4 Predictions for the Cα-Cβ-Ks ecosystem

So far we have developed quantitative models for the growth of Cα, Cβ and Ks monocultures. We also characterized the cooperation strength between Cα and Cβ, and bacteriocin-introduced inhibition between Ks and Cα (Cβ). Analogous to our experimental community assembly, we used these basic mathematical modules to create an ecosystem model of Cα, Cβ and Ks, and further used it to predict the community dynamics from the bottom up.

Specifically, a three-strain model of Cα-Cβ-Ks can be written as

$$\begin{aligned}
\frac{dN}{dt} &= -\frac{1}{\gamma_{C_\alpha}}\frac{g_{C_\alpha}N}{N_{C_\alpha}+N}C_\alpha - \frac{1}{\gamma_{C_\beta}}\frac{g_{C_\beta}N}{N_{C_\beta}+N}C_\beta - \frac{1}{\gamma_{Ks}}\frac{g_{Ks}N}{N_{Ks}+N}K_s \\
\frac{dC_\alpha}{dt} &= g_{C_\alpha}\frac{N}{N_{C_\alpha}+N}C_\alpha - I_{C_\alpha,A}\frac{A}{K_{C_\alpha,A}+A}C_\alpha \\
\frac{dC_\beta}{dt} &= g_{C_\beta}\frac{N}{N_{C_\beta}+N}C_\beta - I_{C_\beta,A}\frac{A}{K_{C_\beta,A}+A}C_\beta \\
\frac{dK_s}{dt} &= g_{Ks}\frac{N}{N_{Ks}+N}K_s - I_{Ks,G}\frac{G}{K_{Ks,G}+G}K_s \\
\frac{d\alpha}{dt} &= p_\alpha C_\alpha - k_{\alpha\beta}\alpha\beta \\
\frac{d\beta}{dt} &= p_\beta C_\beta - k_{\alpha\beta}\alpha\beta \\
\frac{dG}{dt} &= k_{\alpha\beta}\alpha\beta - d_G G \\
\frac{dA}{dt} &= p_A K_s - d_A A
\end{aligned} \tag{9}$$

where the parameters are summarized in Supplementary Tables 5 and 6. Notably, all of the parameter values were inherited directly from the previous characterizations of individual modules and no new parameters were introduced.

Ecosystem succession when cooperation variations arise from labor partition. Using the model (Supplementary Equation 9), we simulated the dynamics of Cα-Cβ-Ks ecosystem starting from the initial total OD of 10^{-2} and a fixed (Cα+Cβ):Ks ratio (2:1) without changing any of the parameters. The initial Cα:Cβ partition was varied from 30:1 to 10:1, 3:1, 1:1, 1:3, 1:10 and 1:30. The resulting simulated ecosystem dynamics were shown in Figure 3c in the main text.

Ecosystem succession when cooperation variations arises from sampling. To investigate the ecosystem dynamics upon random sampling, we also simulated the three-strain model (Supplementary Equation 9) under different total initial ODs. Using the similar procedure as we did for the *in silico* of serial dilution for the Cα-Cβ ecosystem (Supplementary Section 1.1.2, **lcnG productivity in serial dilution**), we simulated the sequential dilution process for the Cα-Cβ-Ks co-culture from a total OD of 1.0 to 10^{-2}, 10^{-4}, 10^{-6} and 10^{-8}. As the ecosystem has three instead of two strains, in each round of dilution (say, the i-th dilution), determining the initial concentration needs to be modified into the following steps: First, sample the Cα-Cβ mixed population (Step 1 in the two-strain dilution) and its Cα fraction (Step 2 in the two-strain dilution) from the last dilution of Cα-Cβ co-culture, and then determine the Cα population and Cβ population using Step 3 in the two-strain dilution. Second, sample Ks population from last round of population using a Gaussian distribution; Third, mix sampled Cα-Cβ population with Ks population in 2:1 ratio to form a consortium of Cα-Cβ-Ks. The corresponding parameter values are shown in Supplementary Tables 7 and 8. With the resulting initial ODs of the three strains, we conducted computational ecosys-

tem successions by numerically solving Supplementary Equation 9. The model-predicted Ks dynamics are shown in Figure 4a-d in the main text.

1.2 Modeling the Cα-Cβ-Kr ecosystem

As a comparison to the Cα-Cβ-Ks ecosystem involving a variable cooperation, we also created a consortium of the cooperators, Cα and Cβ, and another third strain Kr, which is resistant to lcnG produced by Cα and Cβ and deficient in secreting lcnA that suppresses the cooperators (Figure 3e, f in the main text). Using a bottom-up assembly approach, we developed a dynamic community model of the Cα-Cβ-Kr ecosystem and used it to predict the ecosystem's dynamics.

1.2.1 Monoculture Kr growth

We modeled Kr growth in batch culture in terms of Monod equation

$$
\begin{aligned}
\frac{dN}{dt} &= -\frac{1}{\gamma_{Kr}}\frac{g_{Kr}N}{N_{Kr}+N}K_r \\
\frac{dK_r}{dt} &= \frac{g_{Kr}N}{N_{Kr}+N}K_r
\end{aligned} \tag{10}
$$

where N is the nutrient level, K_r is the Kr population density, g_{Kr} is the maximum growth rate, N_{Kr} is the corresponding half-saturation constants, and γ_{Kr} is Kr's growth yield coefficient. By fitting Supplementary Equation 10 to the experimental data in Supplementary Figure 10, we obtained the growth parameters of Kr (Supplementary Table 5).

1.2.2 Modeling and predictions of the Cα-Cβ-Kr ecosystem

We also constructed a mathematical model of the Cα-Cβ-Kr ecosystem as

$$
\begin{aligned}
\frac{dN}{dt} &= -\frac{1}{\gamma_{C_\alpha}}\frac{g_{C_\alpha}N}{N_{C_\alpha}+N}C_\alpha - \frac{1}{\gamma_{C_\beta}}\frac{g_{C_\beta}N}{N_{C_\beta}+N}C_\beta - \frac{1}{\gamma_{Kr}}\frac{g_{Kr}N}{N_{Kr}+N}K_r \\
\frac{dC_\alpha}{dt} &= g_{C_\alpha}\frac{N}{N_{C_\alpha}+N}C_\alpha \\
\frac{dC_\beta}{dt} &= g_{C_\beta}\frac{N}{N_{C_\beta}+N}C_\beta \\
\frac{dK_r}{dt} &= g_{Kr}\frac{N}{N_{Kr}+N}K_r
\end{aligned} \tag{11}
$$

where N, C_α, C_β, and K_r are the nutrient level, Cα concentration, Cβ concentration and Kr concentration accordingly. Parameters are the same as previously defined and listed in Supplementary Table 5.

Importantly, compared to the model of Cα-Cβ-Ks ecosystem, this model does not involve the kinetics of lcnG and lcnA and has no inhibition terms mediated by these two bacteriocins. This echoes with the fact that Kr is resistant to lcnG produced by Cα-Cβ co-culture and deficient in secreting lcnA to suppress Cα and Cβ. In addition, except for the Kr

growth parameters that are derived from monoculture characterization, all other parameters were inherited directly from previous characterizations of individual modules (e.g., Cα-Cβ consortium).

Using the model Equation 11, we simulated the dynamics of Cα-Cβ-Kr ecosystem under various Cα:Cβ partitions but a fixed initial total OD of 10^{-2} and a fixed (Cα+Cβ):Kr ratio (2:1). The specific initial Cα:Cβ ratio was changed from 30:1 to 10:1, 3:1, 1:1, 1:3, 1:10 and 1:30, resulting in the corresponding dynamics in Figure 3g in the main text.

1.3 Modeling the Cα-Cβ-Kp ecosystem

To further advance our understanding on the role of interaction variability, we experimentally created an ecosystem of Cα, Cβ and Kp, another third strain which has pH dependence on lcnG resistance and lcnA production. In conjugation with the experiment, we built a corresponding community model using again the bottom-up assembly method.

1.3.1 Kp monoculture growth

The general form of Kp growth dynamics can be described as

$$\begin{aligned} \frac{dN}{dt} &= -\frac{1}{\gamma_{Kp}} \frac{g_{Kp} N}{N_{Kp} + N} K_p \\ \frac{dK_p}{dt} &= \frac{g_{Kp} N}{N_{Kp} + N} K_p \end{aligned} \tag{12}$$

where N is the nutrient level, K_p is the Kp population density, g_{Ks} is Kp's maximum growth rate, N_{Kp} is the corresponding half-saturation constant, and γ_{Kp} is the growth yield coefficient. When grown in three monoculture settings (pH$\geq$7, pH$\leq$6, and no pH control), Kp exhibits three distinct growth profiles (Supplementary Figure 13a). By fitting the model Equation 12 to the experimental data, we obtained the growth parameters of Kp in the three settings (Supplementary Table 11). The corresponding simulated growth patterns are shown in Supplementary Figure 13b.

1.3.2 Growth and lcnG production of the Cα-Cβ co-culture in pH-controlled settings

The mathematical framework in Section 1.1.2 (Supplementary Equation 4) remains valid for describing the Cα-Cβ co-culture in pH-controlled media. However, their parameters need to be recalibrated as cellular metabolism is highly dependent on the environments. To achieve the goal, we first fitted the monoculture models of Cα and Cβ in Section 1.1.1 (Supplementary Equations 1 and 2) with experimental results in three different environments (Supplementary Figure 16), resulting in the new growth parameters of the Cα and Cβ monocultures (Supplementary Table 11). Using the new monoculture parameters, we then recalibrated the co-culture of Cα and Cβ in terms of growth and lcnG production in three different settings using the same procedure as outlined in Section 1.1.2. As expected, lcnG production of the Cα-Cβ co-culture varied for different media (Supplementary Figure 17a-c)

with the newly assigned values of parameters (p_α, p_β, $k_{\alpha\beta}$, and d_G) listed in Supplementary Table 12.

LcnG productivity under varied labor partitions. Using the recalibrated model with pH-specific parameters, we repeated the dynamics of lcnG production under varied initial Cα-Cβ partitions as we did in Section 1.1.2. The results are shown in Supplementary Figure 17d-f.

LcnG productivity in serial dilution. For serial dilution of the Cα-Cβ, the same experimental procedures were applied to those cases with and without pH controls. Thus, the sampling variations of Cα-Cβ co-culture shall be in principle identical to all pH settings. The corresponding parameters from experiments are listed in Supplementary Tables 7 and 8.

1.3.3 Interactions between Kp and the Cα-Cβ pair

LcnA production by Kp. Similar to lcnA production from strain Ks, we constructed a model of Kp monoculture that constitutively produces lcnA as

$$\begin{aligned} \frac{dN}{dt} &= -\frac{1}{\gamma_{Kp}} \frac{g_{Kp}N}{N_{Kp}+N} K_p \\ \frac{dK_p}{dt} &= g_{Kp} \frac{N}{N_{Kp}+N} K_p \\ \frac{dA}{dt} &= p_A K_p - d_A A \end{aligned} \quad (13)$$

where N, K_p and A refer to the nutrient level, Kp population density and the lcnA concentration in the culture. g_{Kp} is Kp's maximum growth rate, N_{Kp} is the corresponding half-saturation constant and γ_{Kp} is the growth yield coefficient of Kp, parameter p_A is the lcnA production rate, and d_A is the lcnA degradation rate.

Unlike Ks which constitutively produces lcnA, Kp is designed to be pH-dependent for lcnA production (Figure 5c in the main text). Therefore, in addition to the growth parameters g_{Kp}, N_{Kp} and γ_{Kp}, parameter p_A should be environment specific. Meanwhile, the stability of lcnA is subject to pH, thus, d_A should also be determined in environment-specific manners. Both the parameters were determined by fitting the model to the experimental data in three different settings (Supplementary Table 12). The simulated lcnA production profiles in pH≥7, pH≤6 and no pH control settings are shown in Supplementary Figure 14; for the case of pH≥7, $p_A = 0$ held since no lcnA was detected in the overnight incubation. Noteworthy, in the case of no pH control, lcnA was detected after 4 hours of fermentation and eventually accumulated to a medium level, which inspired us to approximate this case as a dynamic fermentation that switches from the pH≥7 mode (promoter 'Off' state) to the pH≤6 mode (promoter 'On' state).

LcnA-mediated Kp-to-Cα/Cβ inhibition. To determine the inhibition, the model of

Kp and Cα (or Cβ) co-culture can be written as

$$\begin{aligned}
\frac{dN}{dt} &= -\frac{1}{\gamma_{C_\alpha}}\frac{g_{C_\alpha}N}{N_{C_\alpha}+N}C_\alpha - \frac{1}{\gamma_{Kp}}\frac{g_{Kp}N}{N_{Kp}+N}K_p \\
\frac{dC_\alpha}{dt} &= g_{C_\alpha}\frac{N}{N_{C_\alpha}+N}C_\alpha - I_{C_\alpha,A}\frac{A}{K_{C_\alpha,A}+A}C_\alpha \\
\frac{dK_p}{dt} &= g_{Kp}\frac{N}{N_{Kp}+N}K_p \\
\frac{dA}{dt} &= p_A K_p - d_A A
\end{aligned} \tag{14}$$

which is the same as the model (Supplementary Equation 6) for describing the Ks-to-Cα/Cβ inhibition except that K_s is replaced by K_p. In addition to the parameters calibrated in the previous subsections, other parameters in the interaction term, $I_{C_\alpha,A}$ and $K_{C_\alpha,A}$, need to be determined in different pH settings. For pH$\geq$7, $I_{C_\alpha,A}$ and $K_{C_\alpha,A}$ both were set to null as no lcnA was detected. For pH$\leq$6, the model Supplementary Equation 14 were fitted to the experiment data (Supplementary Figure 18) to yield the parameters that are shown in Supplementary Table 12. For the case of no pH control, $I_{C_\alpha,A}$ and $K_{C_\alpha,A}$ were set to null at the first 4 hours but, afterwards, were assigned with those of pH$\leq$6 based on our experimental observations in Supplementary Figure 14. The lcnA-mediated inhibition from Kp to Cβ can be described in the same fashion. The corresponding parameters are summarized in Supplementary Table 12.

lcnG-mediated Cα-Cβ pair-to-Kp inhibition. To determine the inhibition of lcnG on Kp in different media, we cultured Kp with the supernatant of Cα-Cβ co-culture at 1:1 ratio (Supplementary Figure 15a,c,e). The corresponding Kp growth model can be expressed as

$$\begin{aligned}
\frac{dN}{dt} &= -\frac{1}{\gamma_{Kp}}\frac{g_{Kp}N}{N_{Kp}+N}K_p \\
\frac{dK_p}{dt} &= g_{Kp}\frac{N}{N_{Kp}+N}K_p - I_{Kp,G}\frac{G}{K_{Kp,G}+G}K_p \\
\frac{dG}{dt} &= -d_G G
\end{aligned} \tag{15}$$

where K_p is introduced to replace K_s in Supplementary Equation 7. Notice that $I_{Kp,G}$ and $K_{Kp,G}$, the parameters characterizing the inhibition strength of lcnG on Kp, are now susceptible to environment pH.

For comparison, we also cultured Kp in the media of pH$\geq$7, pH$\leq$6 and no pH control with the supernatant of Cα-Cβ' co-culture at 1:1 ratio. Since no active lcnG is present in the supernatant of the Cα-Cβ' co-culture, the dynamics of Kp in the three media can be revised as

$$\begin{aligned}
\frac{dN}{dt} &= -\frac{1}{\gamma_{Kp}}\frac{g_{Kp}N}{N_{Kp}+N}K_p \\
\frac{dK_p}{dt} &= g_{Kp}\frac{N}{N_{Kp}+N}K_p
\end{aligned} \tag{16}$$

where the inhibition term is removed.

Similar to the case of Ks cultured in GM17 media mixed with the Cα-Cβ(Cβ') co-culture supernatants (Supplementary Figure 9e,f), our experiments also showed a reduction of Kp density in the first 1.5 hours. Accordingly, in our models, we incorporated this decline phase and used the experimental data of t≥1.5 h (Supplementary Figure 15b,d,f) to extract the growth parameters for the three pH-controlled cases (Supplementary Table 9). Subsequently, for the pH≥7 and pH≤6 cases, parameters relating to the inhibition strength ($I_{Kp,G}$, $K_{Kp,G}$) were determined using Supplementary Equation 15 (Supplementary Table 12).

By fitting the model to the experimental data (Supplementary Figure 15), we acquired Kp's growth rate in three different pH controlled settings (Supplementary Table 9). We also calculated in the three settings the relative growth rate of Kp, defined as the ratio of average Kp growth rate in GM17 mixed with the Cα-Cβ supernatant and the rate in medium with Cα-Cβ' supernatant, showing that the (Cα-Cβ pair)-to-Kp inhibition strength is pH dependent (Figure 5d in the main text, Supplementary Table 10).

1.3.4 Predictions for the Cα-Cβ-Kp ecosystem

Building on the calibrated modules for the growth of Cα, Cβ and Kp monocultures, the cooperation between Cα and Cβ, and bacteriocin-introduced inhibition between Kp and Cα (Cβ), we assembled a model for the Cα-Cβ-Kp ecosystem in a bottom-up way. Specifically, the three-strain model of Cα-Cβ-Kp can be written as

$$\begin{aligned}
\frac{dN}{dt} &= -\frac{1}{\gamma_{C_\alpha}}\frac{g_{C_\alpha}N}{N_{C_\alpha}+N}C_\alpha - \frac{1}{\gamma_{C_\beta}}\frac{g_{C_\beta}N}{N_{C_\beta}+N}C_\beta - \frac{1}{\gamma_{Kp}}\frac{g_{Kp}N}{N_{Kp}+N}K_p \qquad (17)\\
\frac{dC_\alpha}{dt} &= g_{C_\alpha}\frac{N}{N_{C_\alpha}+N}C_\alpha - I_{C_\alpha,A}\frac{A}{K_{C_\alpha,A}+A}C_\alpha\\
\frac{dC_\beta}{dt} &= g_{C_\beta}\frac{N}{N_{C_\beta}+N}C_\beta - I_{C_\beta,A}\frac{A}{K_{C_\beta,A}+A}C_\beta\\
\frac{dK_p}{dt} &= g_{Kp}\frac{N}{N_{Kp}+N}K_p - I_{Kp,G}\frac{G}{K_{Kp,G}+G}K_p\\
\frac{d\alpha}{dt} &= p_\alpha C_\alpha - k_{\alpha\beta}\alpha\beta\\
\frac{d\beta}{dt} &= p_\beta C_\beta - k_{\alpha\beta}\alpha\beta\\
\frac{dG}{dt} &= k_{\alpha\beta}\alpha\beta - d_G G\\
\frac{dA}{dt} &= p_A K_p - d_A A
\end{aligned}$$

whose parameters are summarized in Supplementary Tables 11 and 12. Importantly, the parameter values used to make predictions were directly obtained from individual characterized modules and no new parameters were introduced. Remarkably, these parameters are associated with environmental pH.

Ecosystem succession driven by labor partition and pH variations. Using the model

(Supplementary Equation 17), we simulated the dynamics of Cα-Cβ-Kp ecosystem under varied initial Cα:Cβ partitions from 30:1 to 1:30 and varied pH settings from pH$\geq$7 to pH$\leq$6 and no pH control, as for the Cα-Cβ-Ks ecosystem. The initial total OD was chosen as 10^{-2} and the (Cα+Cβ):Kp ratio was fixed as 2:1. The resulting ecosystem simulations are shown in Figure 6d-f in the main text.

Ecosystem succession driven by sampling and pH variations. We also simulated the Cα-Cβ-Kp ecosystem under various total initial ODs (10^{-2}, 10^{-4}, 10^{-6} and 10^{-8}) at different pH settings. The starting Cα:Cβ:Kp ratio at initial OD of 1.0 was fixed at 1:1:1. Here, the simulated sampling procedure is identical to that for the *in silico* of serial dilution of Cα-Cβ-Ks ecosystem (Section 1.1.4). The predicted community behaviors are shown in Figure 7a-d,i-l,q-t in the main text.

2 Supplementary Figures

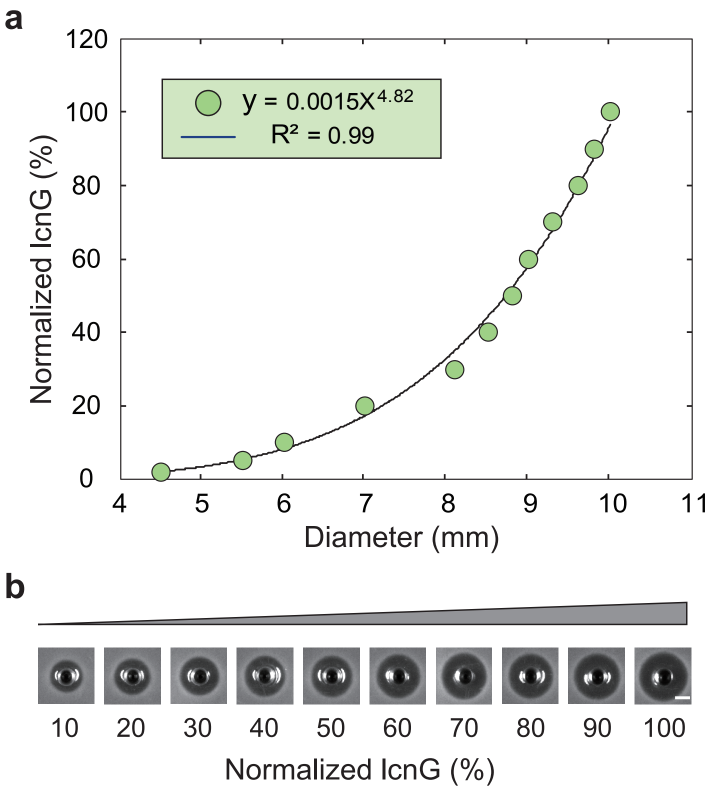

Supplementary Figure 1: **Quantification of relative lcnG concentration. a** Standard curve of relative lcnG concentration. The concentration of lcnG in C$\alpha\beta$ culture at the stationary phase is defined as the reference concentration (100%). It is used to quantify the relative lcnG level of culture samples. The treated samples were diluted with fresh GM17 medium supplemented with 5 μg mL^{-1} chloramphenicol to the relative lcnG concentrations of 90%, 80%, 70%, 60%, 50%, 40%, 30%, 20%, 10%, 5%, 2% and 1%. The agar diffusion assay provides a standard curve for calculating the relative lcnG concentrations of tested samples in the experiments. **b** Representative inhibition zones formed by varied relative levels of lcnG from 10% to 100%. Scale bar, 3 mm.

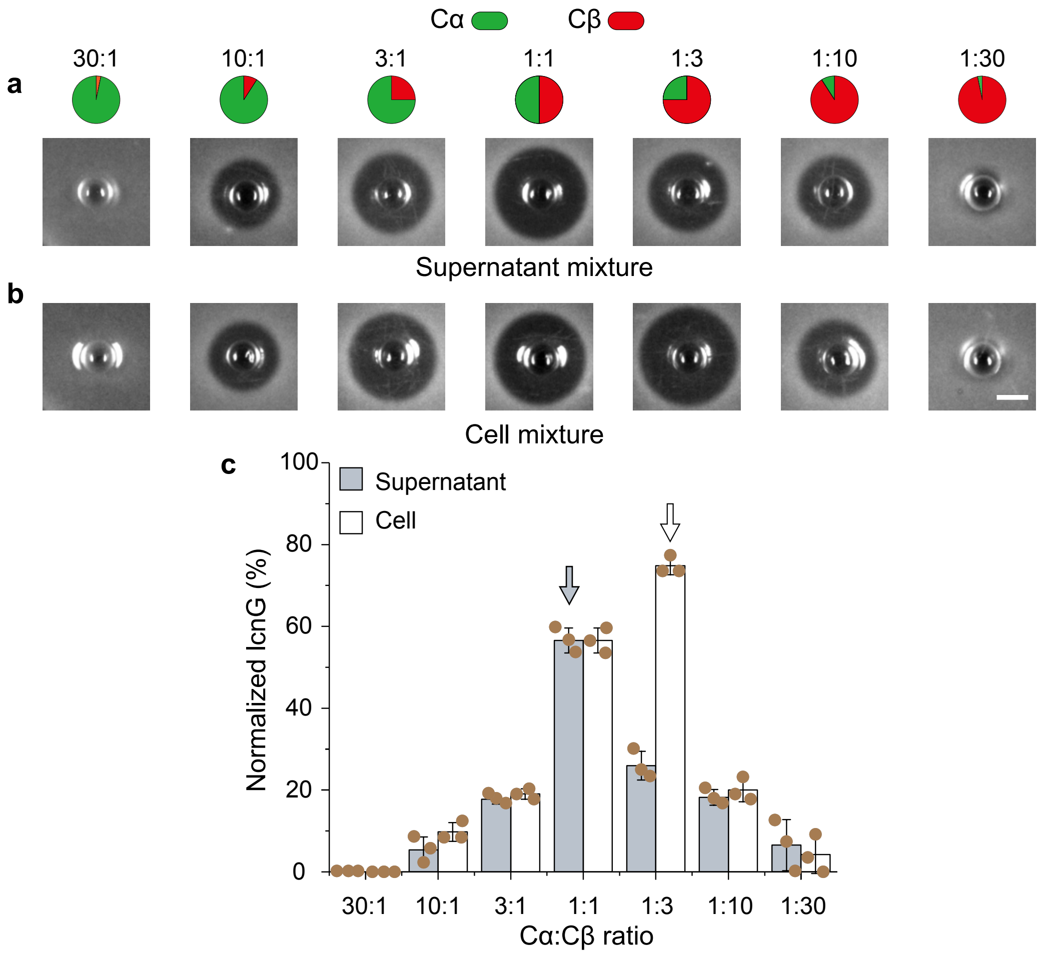

Supplementary Figure 2: **Variation of lcnG production by the Cα-Cβ consortium. a** Representative inhibition zones formed by different ratios of supernatant mixes from Cα and Cβ monocultures. **b** Representative inhibition zones formed by the supernatants of the Cα-Cβ co-cultures growing from different initial Cα:Cβ ratios. In panel **a**, the largest size of inhibition zone is at 1:1 ratio of supernatant mixes. However, in panel **b**, the maximal size was shifted to 1:3 due to the higher growth rate of Cα than that of Cβ. Due to the slower Cβ growth in the competing co-cultures, there was a lower abundance of peptide β in the media. **c** Relative lcnG concentrations in the mixtures of monoculture supernatants and the supernatants of co-cultures (n=3). Here, the lcnG concentration is normalized by the lcnG level in the culture of the lcnG-producing strain C$\alpha\beta$. Data were presented as means and $\pm$ s.d. Scale bar, 3 mm.

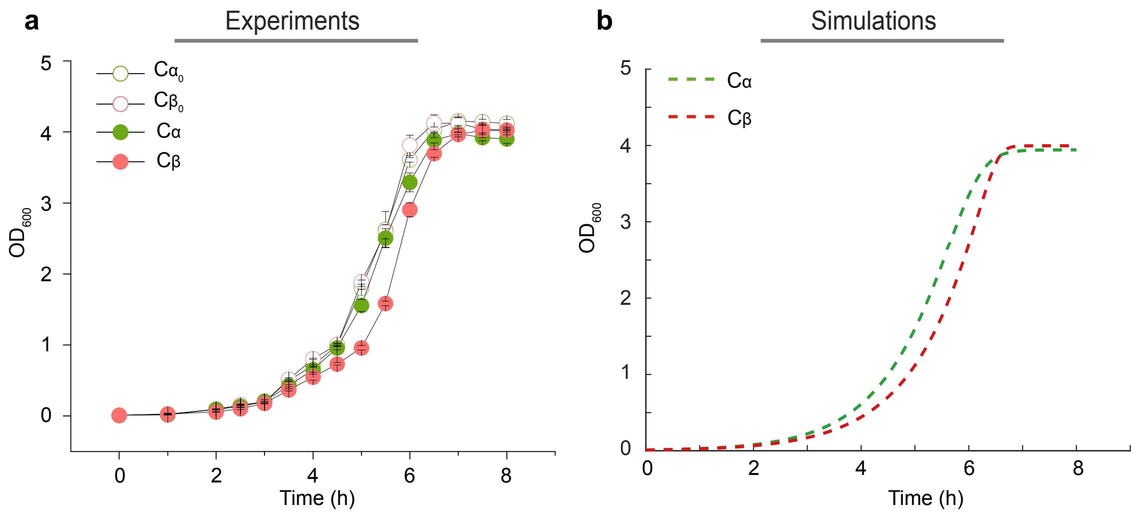

Supplementary Figure 3: **Growth profiles of engineered strains.** **a** Experimental growth profiles of the strains Cα_0 (hollow green circles), Cβ_0 (hollow red circles), Cα (solid green circles) and Cβ (solid red circles). Data were presented as means and $\pm$ s.d. (n = 3). **b** Simulated growth curves of Cα, Cβ using the mathematical model Equations 1-3 under initial ODs of 10^{-2}. Parameters of the model were fitted to the experimental data in panel **a**.

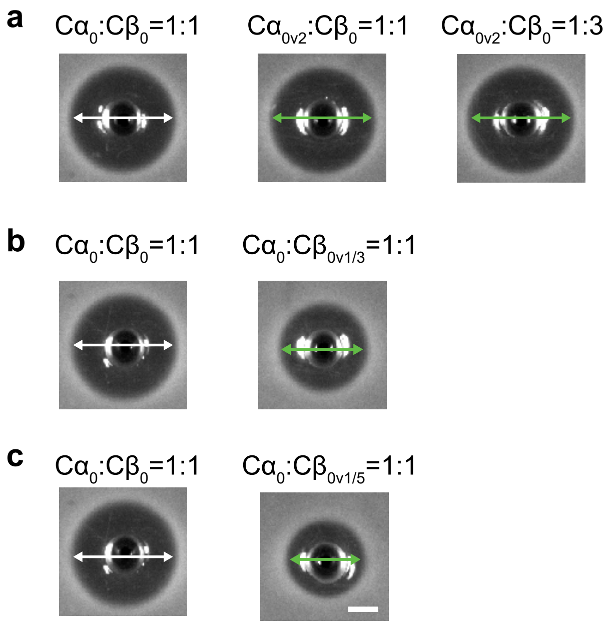

Supplementary Figure 4: **Determination of the lcnG production from the Cα_0 and Cβ_0 variants. a** Representative inhibition zones formed by the supernatant mixes from Cα_0 and Cβ_0 monocultures (1:1, left) and Cα_{0v2} and Cβ_0 monocultures (1:1 (middle) and 1:3 (right)). **b** Representative inhibition zones formed by equal amount of supernatant mixes from Cα_0 and Cβ_0 (left) and Cα_0 and C$\beta_{0v1/3}$ (right). **c** Representative inhibition zones formed by equal amount of supernatant mixes from Cα_0 and Cβ_0 (left) and Cα_0 and C$\beta_{0v1/5}$ (right). Notably, the 1:1 supernatant mixture of Cα_0 and Cβ_0 monocultures was used as control. Scale bar, 3 mm.

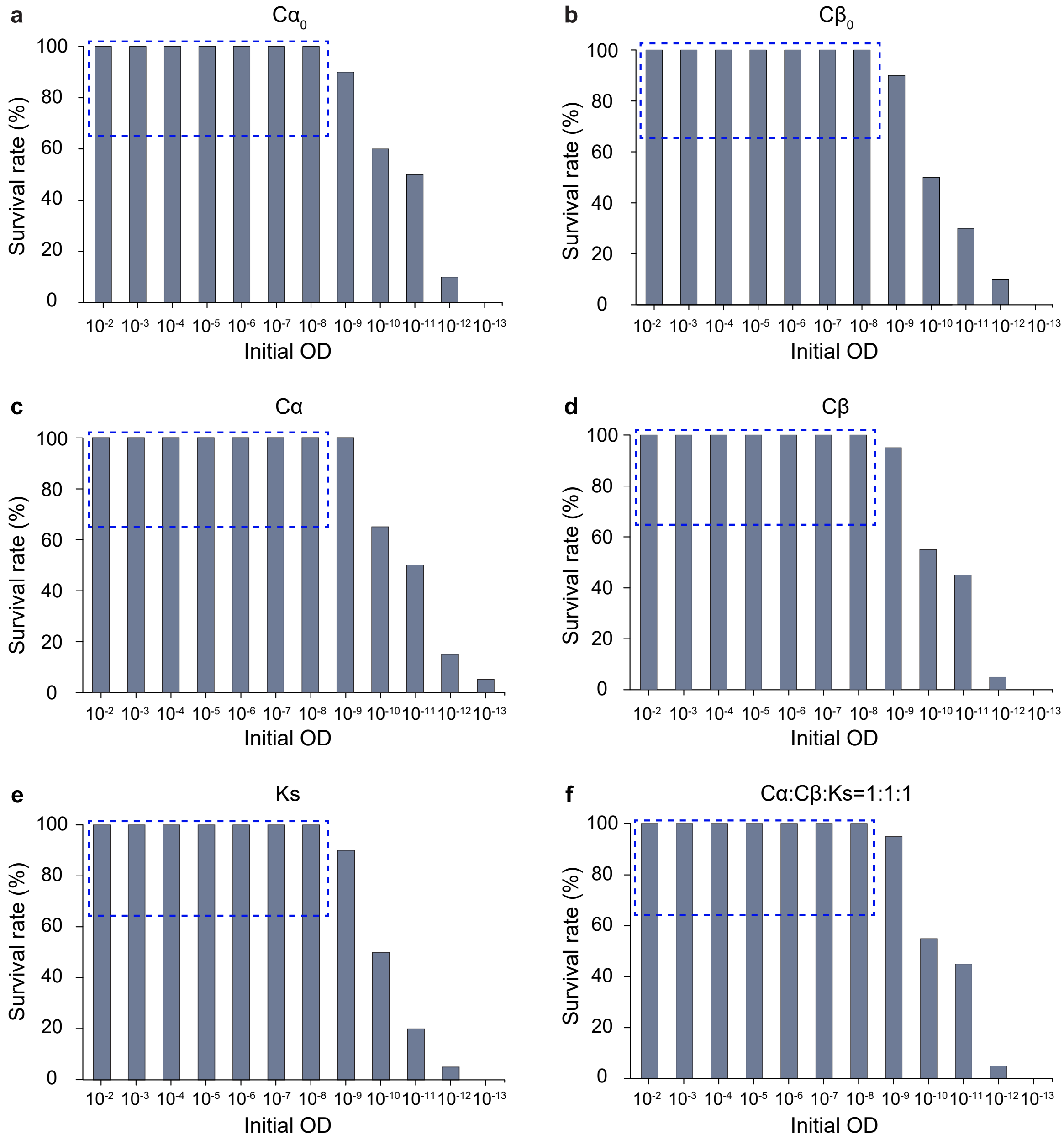

Supplementary Figure 5: **Survival rates of monocultures incubated at different initial ODs.** Survival rates of Cα_0 (**a**), Cβ_0 (**b**), Cα (**c**), Cβ (**d**), and Ks (**e**) monocultures and the Cα-Cβ-Ks co-culture (**f**) in serial 1:100 dilutions. The minimal initial OD_{600} for reliable growth is 10^{-8}. Each dilution was repeated for 20 times in order to create replicates for statistical analysis.

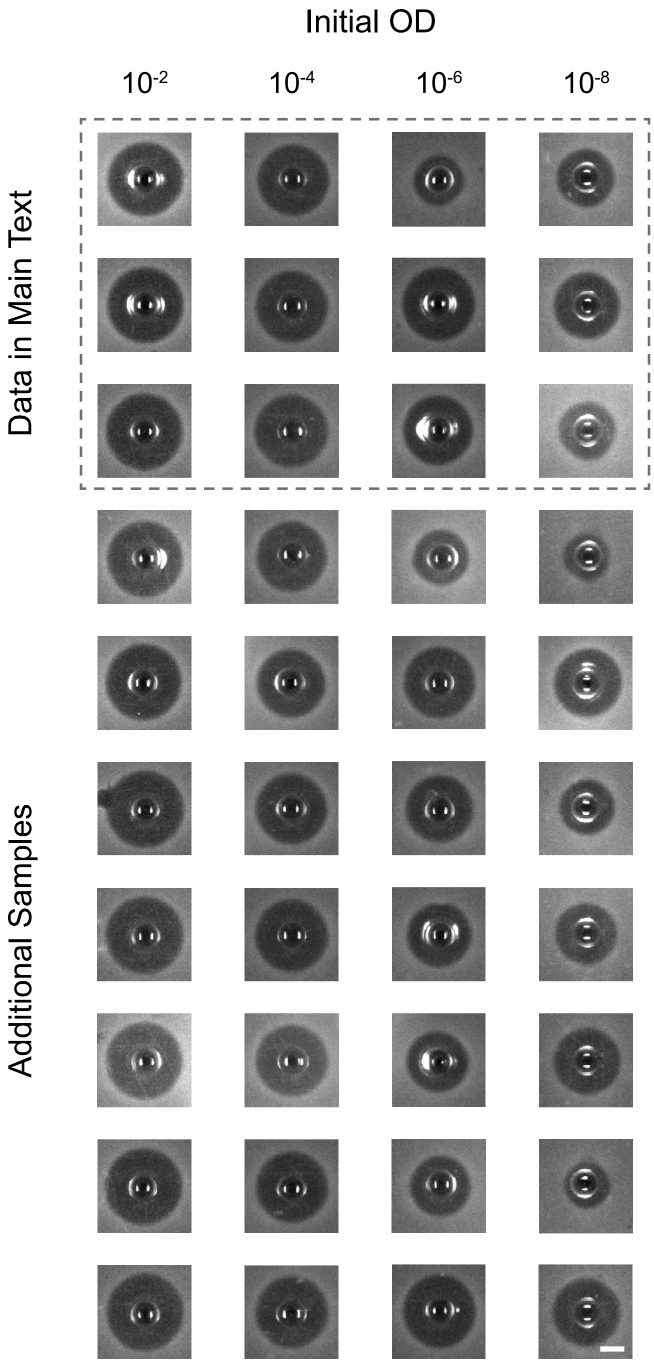

Supplementary Figure 6: **Inhibition zones formed by lcnG from the Cα-Cβ co-cultures growing from different initial ODs.** Representative inhibition zones formed by the supernatants of the Cα and Cβ co-cultures growing from different initial ODs (10^{-2}, 10^{-4}, 10^{-6}, 10^{-8}). The images of inhibition zones in the grey box are identical to those in Figure 2f in the main text, the rest are additional experimental replicates. Scale bar, 3 mm.

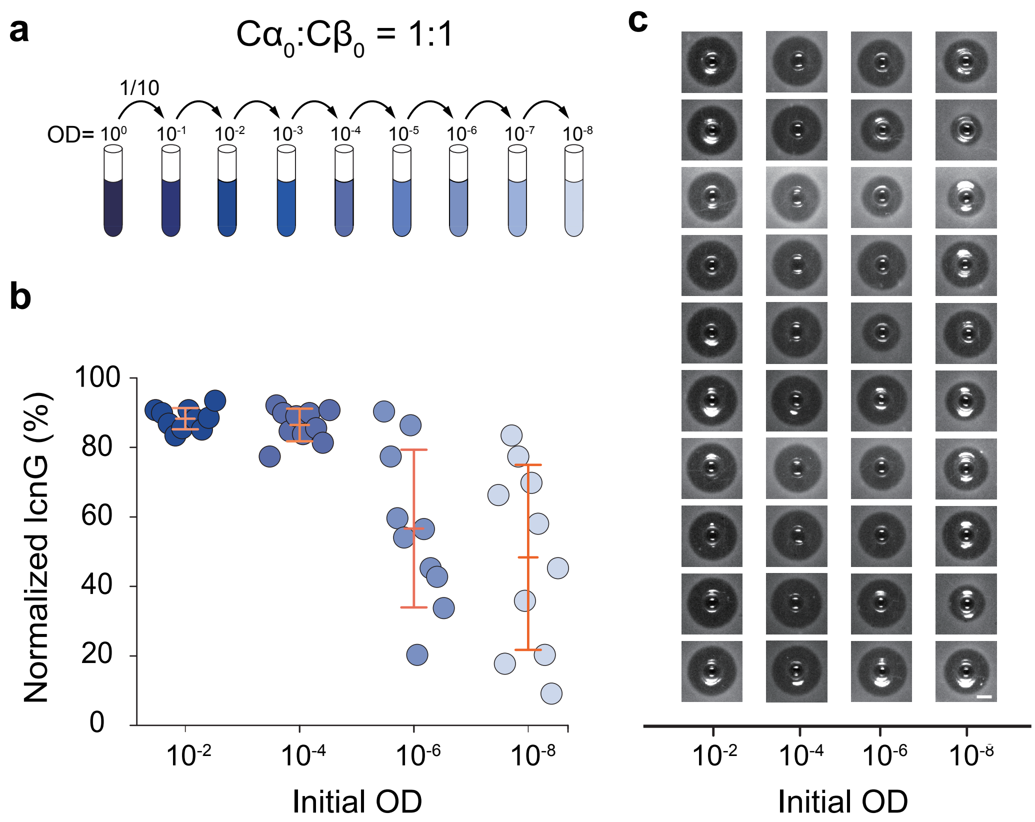

Supplementary Figure 7: Variability of cooperation strength in the Cα_0-Cβ_0 ecosystem. **a** Schematic illustration of serial dilution. **b** Normalized lcnG concentration of Cα_0-Cβ_0 co-culture growing from different initial ODs but with a fixed 1:1 ratio. Each circle corresponds to the lcnG level from an individual experiment. For each OD, there are a total of 10 experimental replicates. Orange lines and bars represent the corresponding means and s.d. **c** Representative inhibition zones formed by the co-culture growing from different initial ODs. Scale bar, 3 mm.

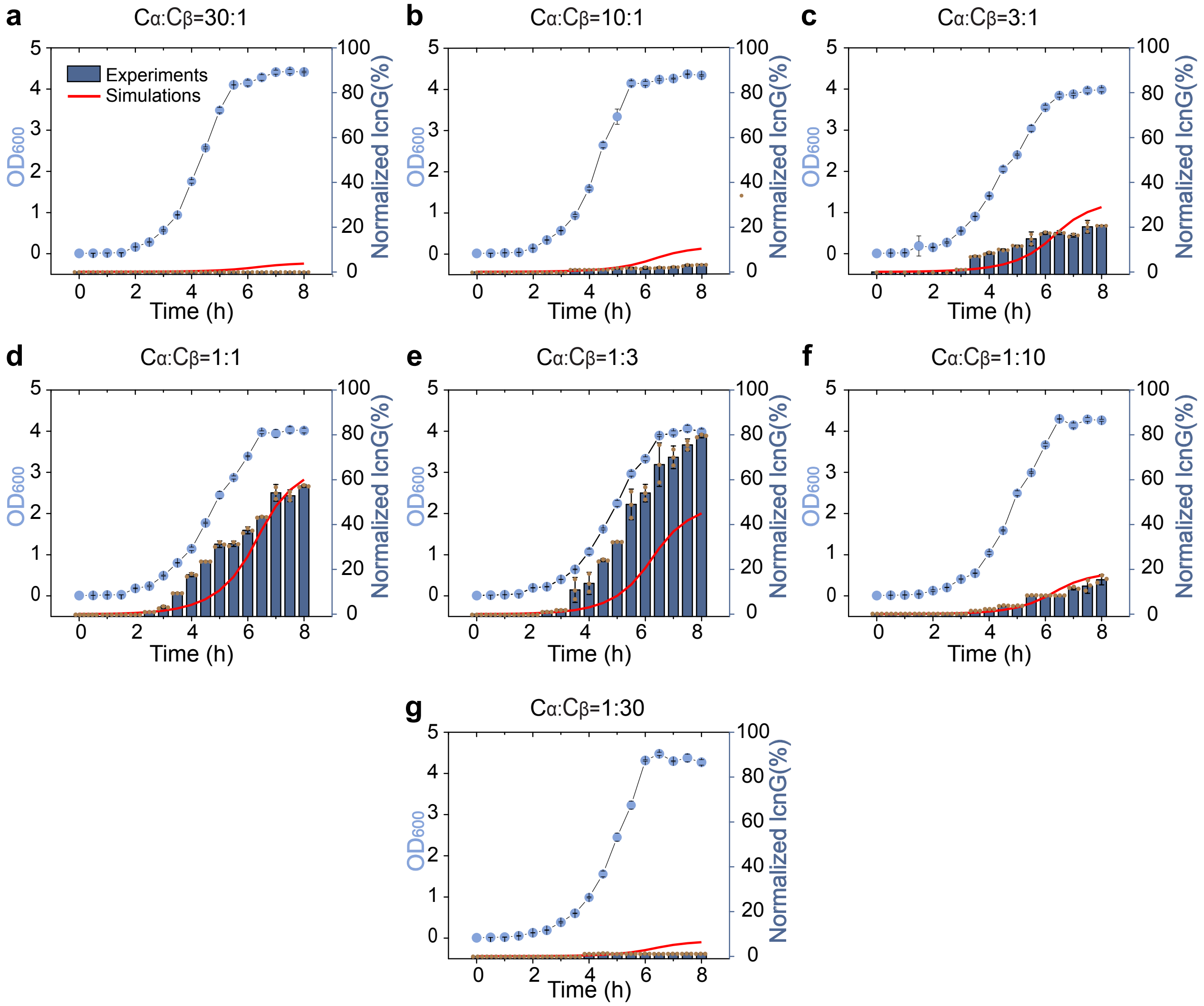

Supplementary Figure 8: Growth and lcnG production of the Cα-Cβ co-cultures growing from different initial Cα:Cβ ratios. The total initial OD was fixed at 0.01 while the relative Cα-to-Cβ ratios were varied from 30:1 (**a**) to 10:1 (**b**), 3:1 (**c**), 1:1 (**d**), 1:3 (**e**), 1:10 (**f**) and 1:30 (**g**). Circles, bars and red lines correspond to the experimental OD, experimental lcnG production and simulated lcnG production. The experimental data were presented as means ± s.d. (n=3). The simulated lcnG production (solid red lines) were obtained by simulating Supplementary Equation 4 with the parameters in Supplementary Table 6.

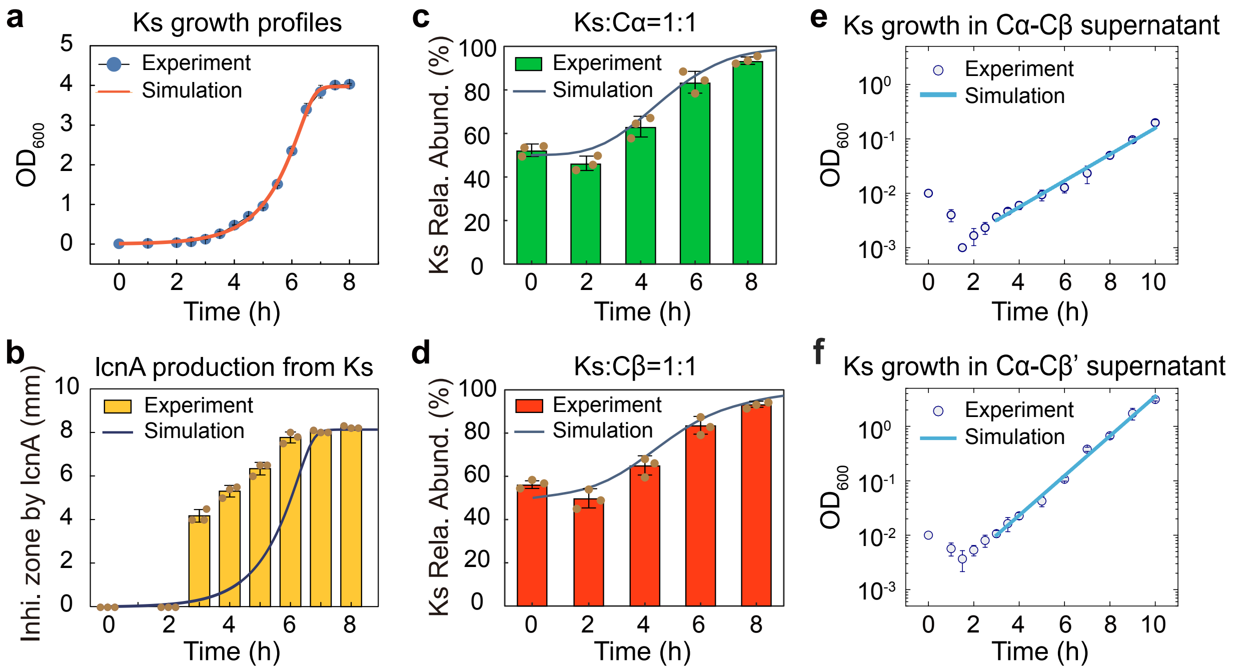

Supplementary Figure 9: **Characterization of the strain Ks.** **a** Experimentally measured and simulated growth profiles of Ks starting from an initial OD of 10^{-2}. The simulation was obtained by fitting the model Equation 3 to the experiment with the parameters specified in Supplementary Table 5. **b** Experimentally measured and simulated lcnA productivity in terms of inhibition zone size for the Ks monoculture in panel **a**. The simulation result was conducted by modeling Supplementary Equation 5 with the parameters specified in Supplementary Table 6. **c, d** Experimental and simulated Ks abundance in the Ks-Cα (**c**) and Ks-Cβ (**d**) co-cultures. Overnight cultures of Ks and Cα (**c**) or Cβ (**d**) were inoculated into fresh GM17/Cm medium at 1:1 initial OD_{600} ratio. The simulations were based on the model (Supplementary Equation 6) with its parameters specified in Supplementary Table 6 to fit the experimental data. **e** Profiles of Ks growing in fresh GM17 medium mixed with the Cα-Cβ co-culture supernatant at 1:1 ratio. **f** Profiles of Ks growing in fresh GM17 medium mixed with the Cα-Cβ' co-culture supernatant at 1:1 ratio. The simulations (gray lines in panels **e** and **f**) were from linear curve fitting the experimental bacterial growth in log phase, and the obtained parameters of average growth rate are specified in Supplementary Table 10.

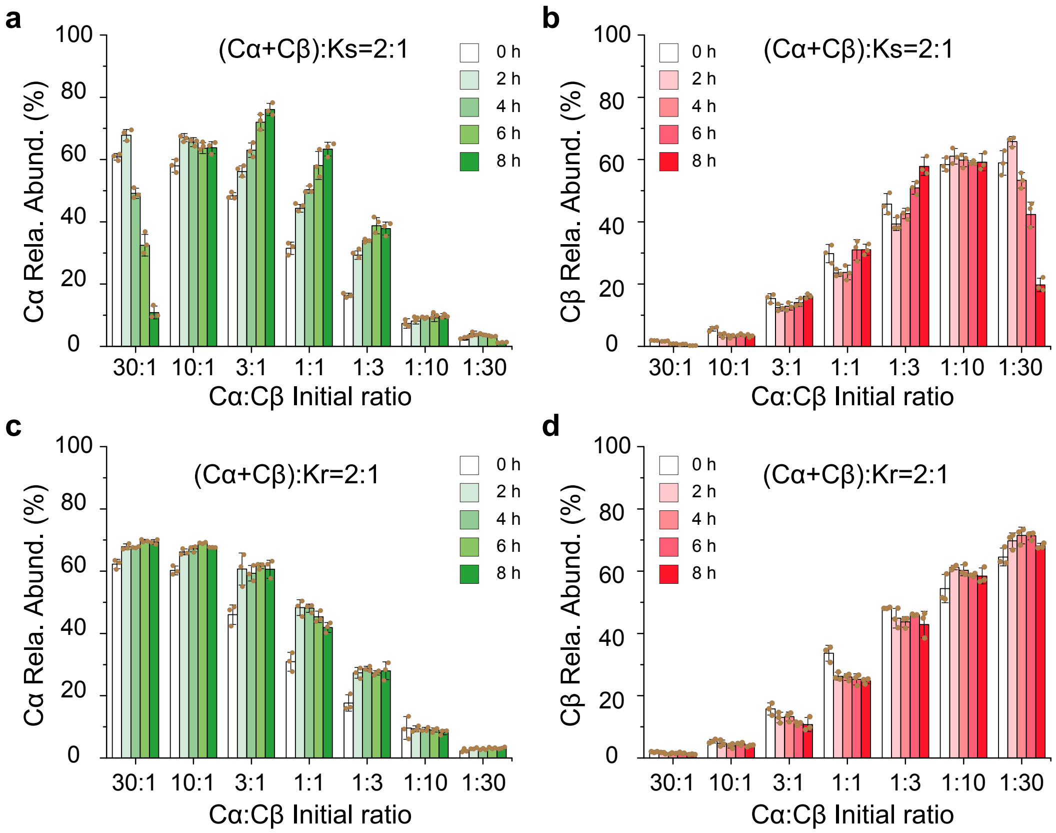

Supplementary Figure 10: Population dynamics of Cα and Cβ in the Cα-Cβ-Ks (**a, b**) and Cα-Cβ-Kr (**c, d**) ecosystems. In the co-culture experiments, initial relative abundance of Ks (Kp) was fixed at 33.3% while the Cα:Cβ ratio was varied from 30:1, 10:1, 3:1, 1:1, 1:3, 1:10 and 1:30. **a** Cα relative abundance in the Cα-Cβ-Ks ecosystem. **b** Cβ relative abundance in the Cα-Cβ-Ks ecosystem. **c** Cα relative abundance in the Cα-Cβ-Kr ecosystem. **d** Cβ relative abundance in the Cα-Cβ-Kr ecosystem. The experimental data were presented as means $\pm$ s.d. (n=3).

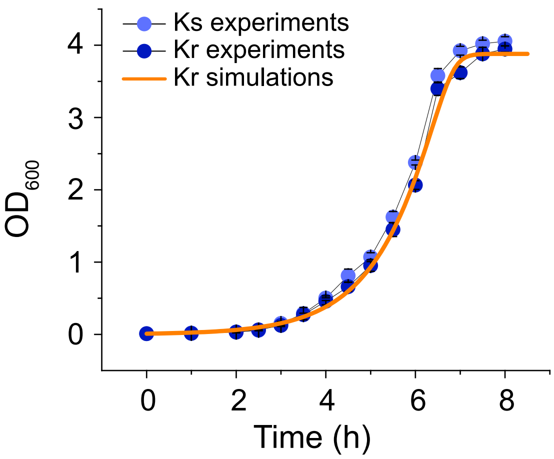

Supplementary Figure 11: **Growth profiles of the strain Kr and Ks.** Light blue and dark blue circles correspond to the experimentally measured growths of Ks and Kr respectively. The orange curve is the simulation result of Kr growth with the parameters in Supplementary Table 5 through data fitting. The experimental data were presented as means ± s.d. (n=3).

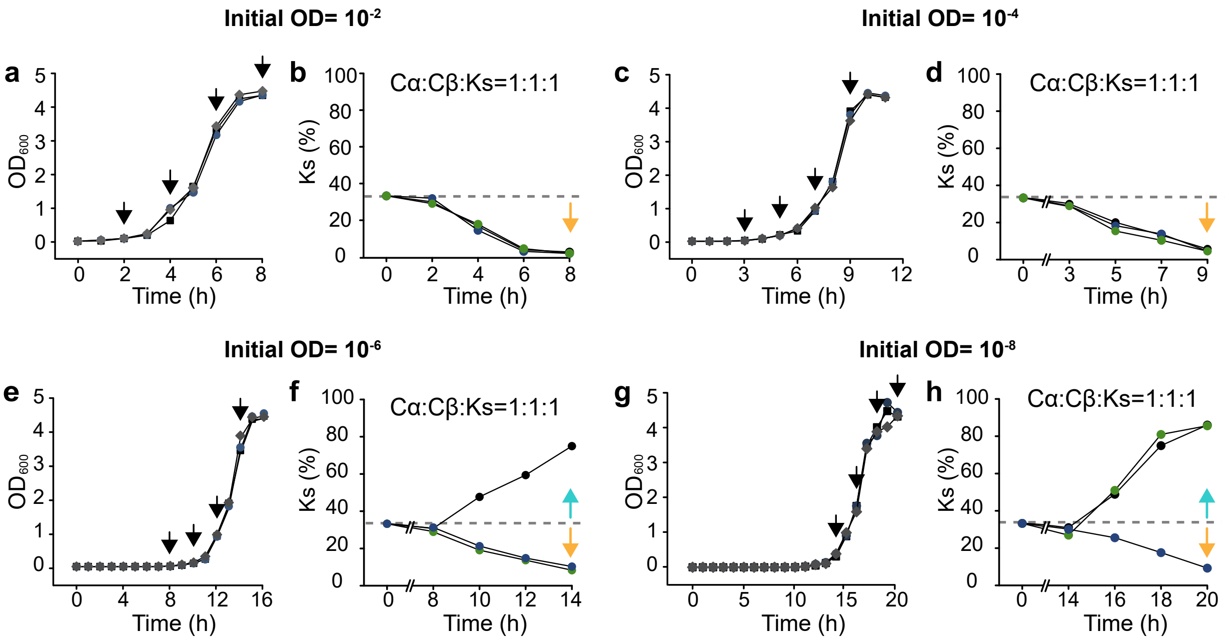

Supplementary Figure 12: **Determination of culturing time and sampling time of the Cα-Cβ-Ks ecosystem.** The initial OD of the ecosystem was varied from 10^{-2} (**a**, **b**) to 10^{-4} (**c**, **d**), 10^{-6} (**e**, **f**) and 10^{-8} (**g**, **h**) while the relative initial ratio was fixed at 1:1:1. As shown in panels **a**, **c**, **e** and **g**, the ecosystem starting at different initial ODs required different culturing times. Notably, for each OD, three replicates were conducted. By comparing these growth curves, we were able to determine the culturing time as 8, 9, 14 and 20 hours for the initial ODs of 10^{-2}, 10^{-4}, 10^{-6} and 10^{-8} respectively. The growth curves also allowed to determine the sampling times for each of the initial ODs, which are indicated with arrows in panels **a**, **c**, **e**, and **g**. As a pilot test, the Ks abundance was measured. Panels **b**, **d**, **f**, and **h** correspond to the initial ODs of 10^{-2}, 10^{-4}, 10^{-6} and 10^{-8} accordingly.

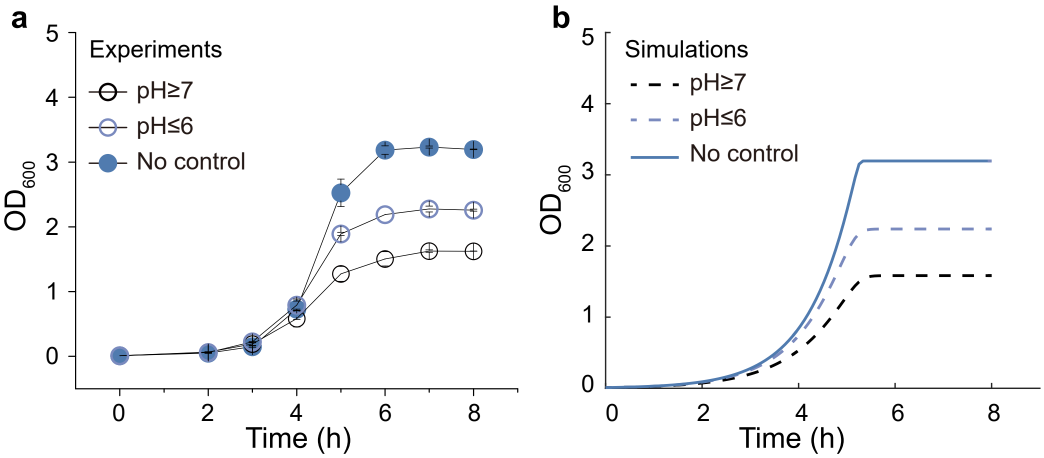

Supplementary Figure 13: **Kp growth in media with different pH controls.** **a** Experimental growth profiles of Kp in pH≥7 (hollow black circles), pH≤6 (hollow blue circles) and no pH control (solid blue circles) fermentation cases. Data was presented as means ± s.d. (n = 3). **b** Simulated growth curves of Kp in pH≥7, pH≤6 and no pH control media from curve fitting. The parameters in the three media were fitted to the experimental data in panel **a** (Supplementary Table 11).

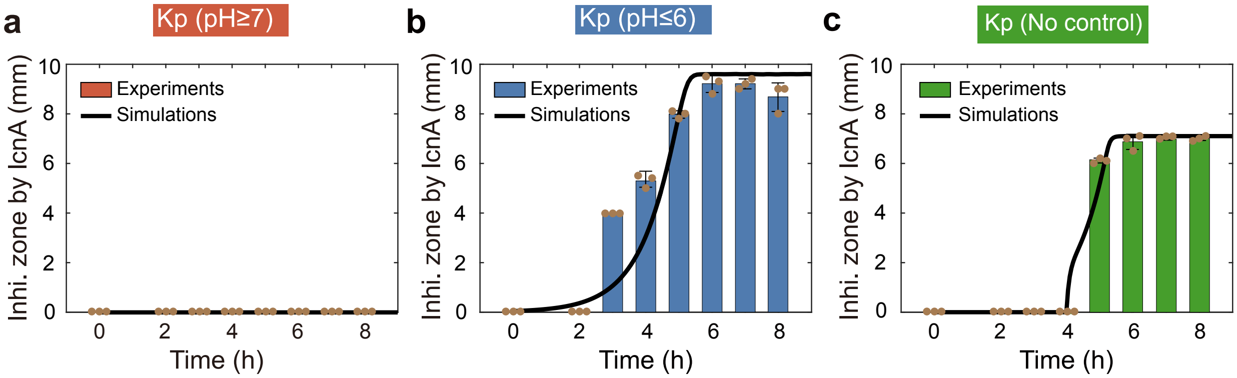

Supplementary Figure 14: **LcnA production of Kp in the media of pH≥7 (a), pH≤6 (b) and no pH control (c)**. Kp monoculture was inoculated as an initial OD of 0.01; at each hour, supernatants were measured by agar-diffusion assay. The bars correspond to the experimental measurements and the lines are simulation results using Supplementary Equation 13. Model parameters are listed in Supplementary Table 12.

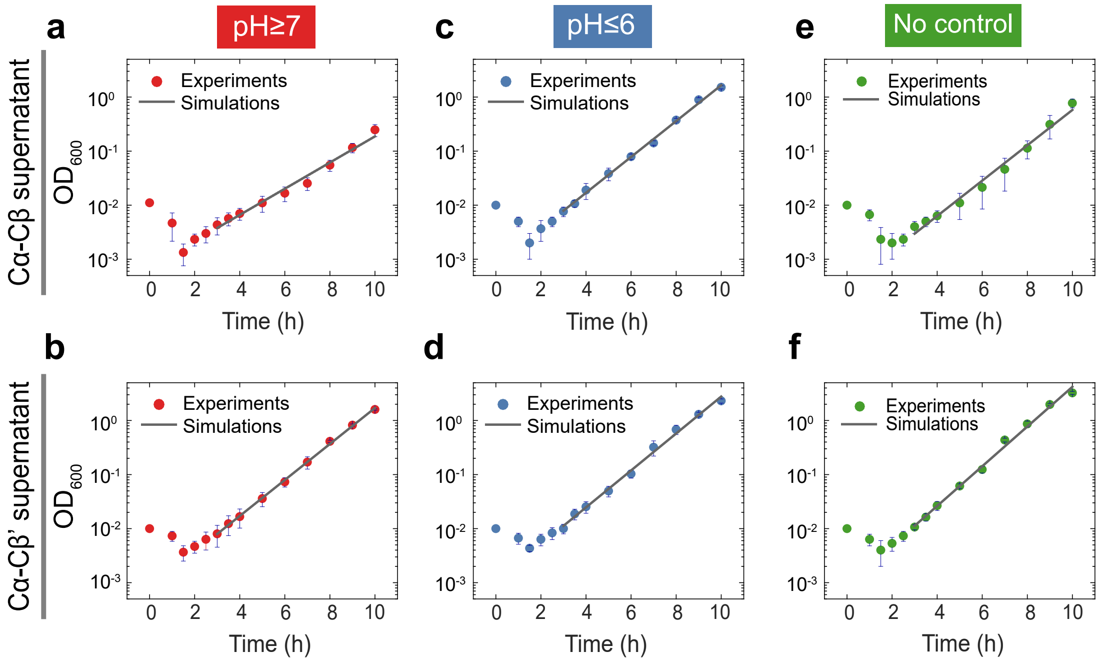

Supplementary Figure 15: **Kp growth in diverse pH-controlled media containing active and inactive lcnG. a, c, e** Kp growth in GM17/Cm medium mixed with the supernatant of Cα-Cβ co-culture in pH≥7 (**a**), pH≤6 (**c**) and no pH control (**e**) settings. The medium contained lcnG produced by Cα-Cβ mixture in this case. **b, d, f** Kp growth in GM17/Cm medium mixed with the supernatant of Cα-Cβ' co-culture in pH≥7 (**b**), pH≤6 (**d**) and no pH control (**f**) settings. The medium did not contain active lcnG because Cα-Cβ' cannot produce active lcnG. For all experiments, the initial ODs of Kp were set as 0.01, dots are experimental data and lines are from data fitting. The experiments showed the reduction of cell growth in the first 1.5 hours, owing to the combination of lag phase and the residual toxins from the supernatants; later, population grew exponentially. The dots are experimental measures, lines are linear curve fitting of the data in log phase. The average growth rates are specified in Supplementary Table 10.

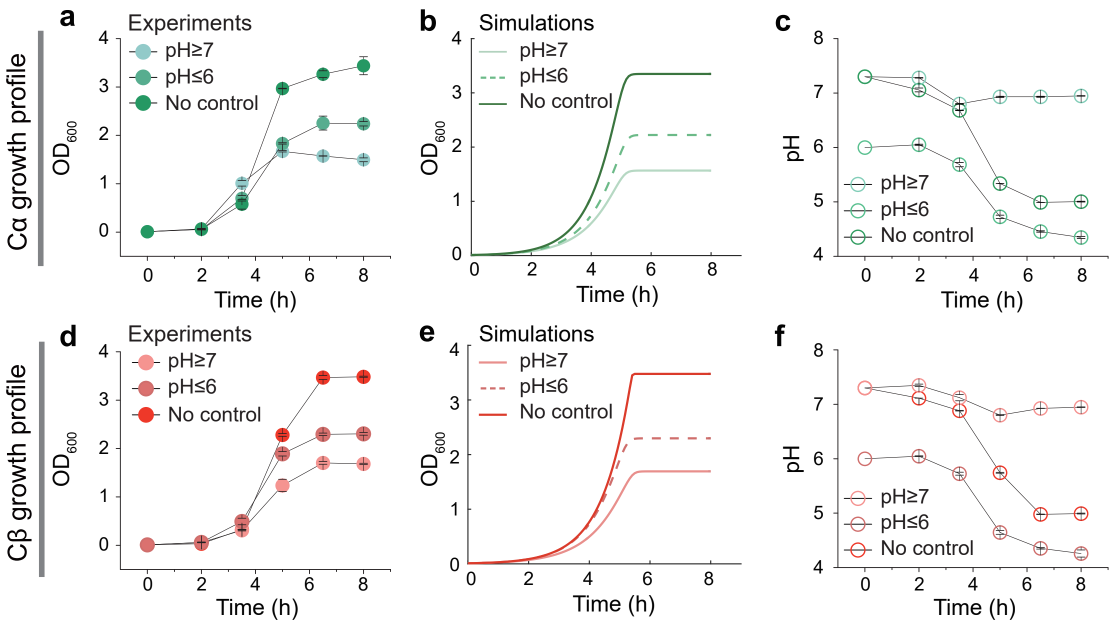

Supplementary Figure 16: **Growth and extracellular pH profiles of Cα and Cβ monocultures in pH-controlled media.** **a** Experimentally measured growth curves of Cα in different pH-controlled media. **b** Simulated growth curves of Cα in different pH-controlled media. **c** Measured extracellular pH patterns of the corresponding cultures of Cα. **d** Experimentally measured growth curves of Cβ in different pH-controlled media. **e** Simulated growth curves of Cβ in different pH-controlled media. **f** Measured extracellular pH patterns of the corresponding cultures of Cβ. Simulated growth curves were obtained by fitting the experimental data in panel **a** and **d**, respectively. The corresponding parameters are listed in Supplementary Table 11.

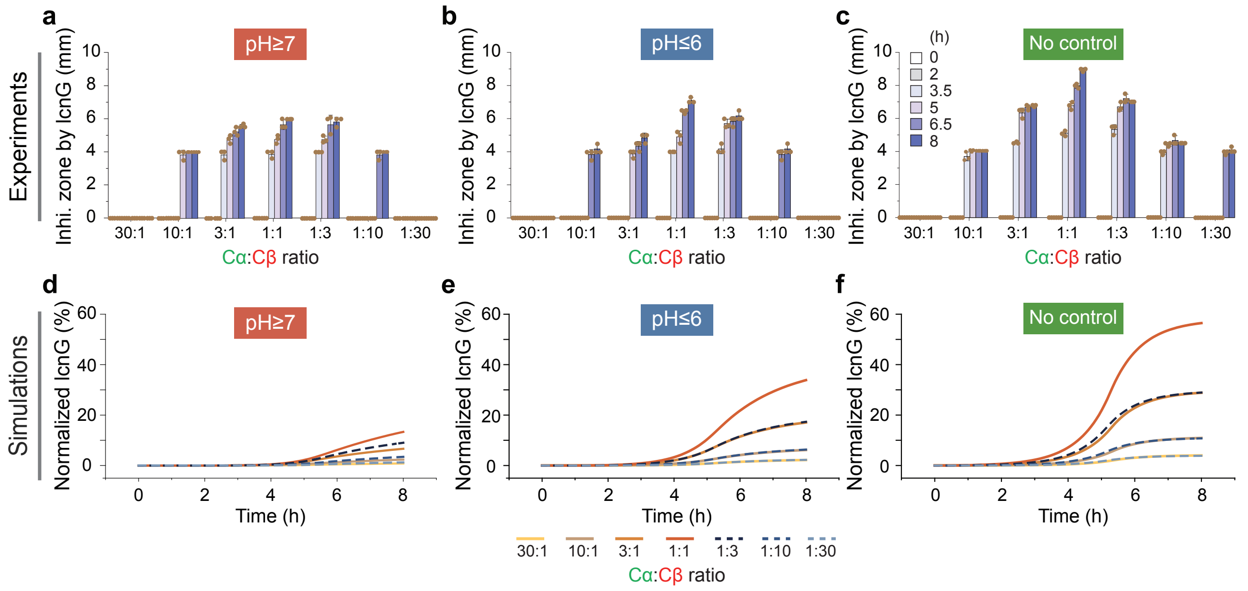

Supplementary Figure 17: **LcnG levels of the Cα-Cβ co-cultures growing in different pH-controlled settings. a-c** Experimentally measured sizes of the inhibition zones formed by the supernatants of Cα-Cβ co-cultures with different initial Cα:Cβ ratios in the pH$\geq$7, pH$\leq$6 and no pH control media. **d-f** Simulated time course of lcnG production for different initial Cα:Cβ ratios corresponding to panels **a-c**. The solid and dashed lines are simulations utilizing the mathematical model (Supplementary Equation 4) with parameters in Supplementary Table 12.

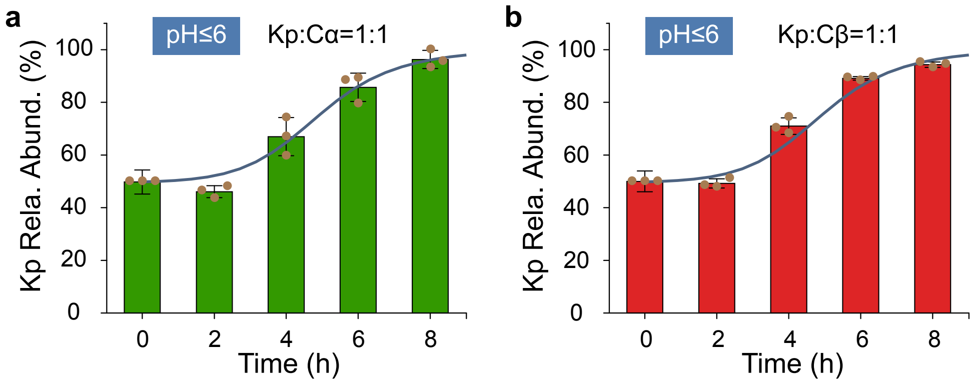

Supplementary Figure 18: **Relative Kp abundance in the Kp-Cα and Kp-Cβ ecosystems growing in the pH$\leq$6 medium. a** Experimental and simulated Kp abundance in the Kp-Cα co-culture. **b** Experimental and simulated Kp abundance in the Kp-Cβ co-culture. For the both experiments, overnight Kp monoculture was mixed with equal amount of Cα or Cβ overnight monoculture based on OD_{600}. The initial total OD was set at 0.01. The relative Kp abundance was measured at 2 h, 4 h, 6 h and 8 h. The simulations are based on Supplementary Equation 14 with the parameters specified in Supplementary Table 12.

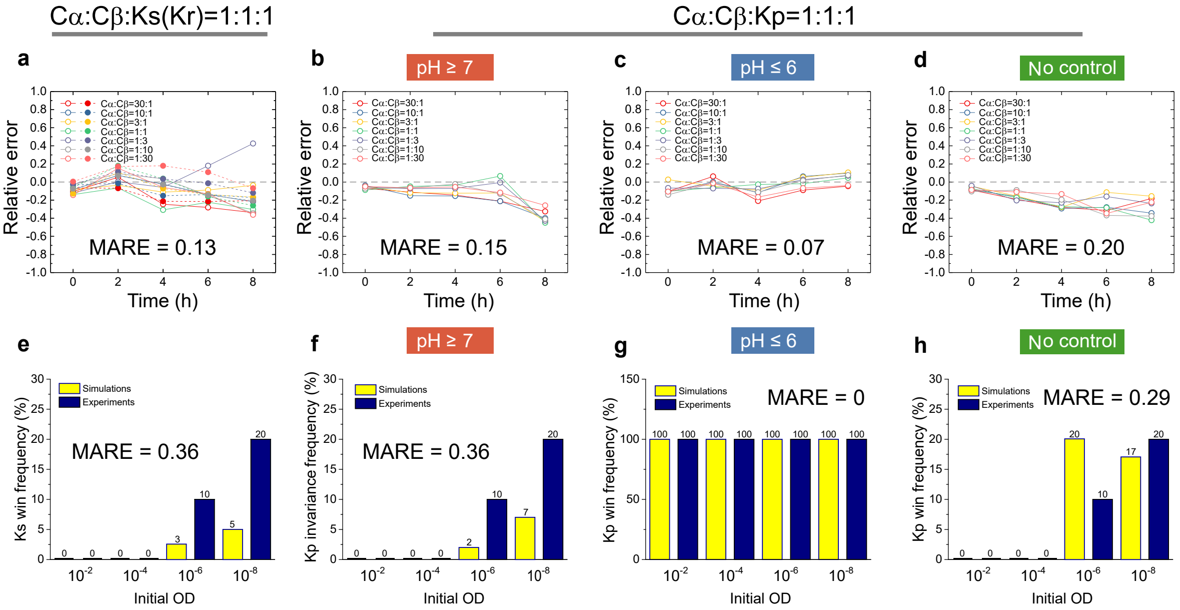

Supplementary Figure 19: **Statistical analysis of model-experiment comparisons. a** Relative error of Ks (Kr) abundance from modeling compared to experimental data for the Cα-Cβ-Ks (Kr) ecosystems. The mean absolute relative error (MARE) of all of the data is 0.13. **b-d** Relative modeling error of Kp abundance for the Cα-Cβ-Kp ecosystems under three different pH controls. The MAREs are 0.15 (**b**), 0.07 (**c**) and 0.20 (**d**) respectively. **e** Modeling-experiment comparison of the probability of Ks dominance in the Cα-Cβ-Ks ecosystem. The MARE is 0.36. **f-h** Modeling-experiment comparison of the Kp dominance in the Cα-Cβ-Kp ecosystem at pH$\geq$7 (**f**), pH$\leq$6 (**g**) and no pH control (**h**). The MAREs are 0.36 (**f**), 0 (**g**) and 0.29 (**h**) accordingly. The modeling and experimental data in this figure are obtained from Figs. 3-7 in the main text.

3 Supplementary Tables

Supplementary Table 1: Strains and plasmids used in this work[4–6] .

Name	Description	Source or Reference
**Strains**		
L. lactis MG1363	Host strain for bacteriocin expression in this work	(4)
L. lactis LMG 2081	Wild-type lactococcin G producing strain	(5)
$C\alpha_0$	Reporter free α peptide producing strain; Contains plasmid pleiss-$C\alpha_0$	This work
$C\beta_0$	Reporter free β peptide producing strain; Contains plasmid pleiss-$C\beta_0$	This work
Cα	α peptide producing strain with a GFP reporter; Contains plasmid pleiss-Cα	This work
Cβ	β peptide producing strain with a RFP reporter; Contains plasmid pleiss-Cβ	This work
Cβ’	β peptide-deficient strain with a RFP reporter; Contains plasmid pleiss-Cβ’	This work
$C\alpha_{0v2}$	Reporter free α peptide producing strain with increased productivity of α peptide; Contains plasmid pleiss-$C\alpha_0$-$P_4\alpha$	This work
$C\beta_{0v1/3}$	Reporter free β peptide producing strain with decreased productivity of β peptide; Contains plasmid pleiss-$C\beta_{0v1/3}$	This work
$C\beta_{0v1/5}$	Reporter free β peptide producing strain with decreased productivity of β peptide; Contains plasmid pleiss-$C\beta_{0v1/5}$	This work
Ks	Lactococcin A producing strain with a GusA3 reporter; Contains plasmid pleiss-lcnA-GusA3	This work
Kr	A control strain with immunity to lcnG and a GusA3 reporter; Contains plsmid pleiss-IG-GusA3	This work
L. lactis NZ9000 /pleiss-Nuc	Indicator strain for agar diffusion assay; *L. lactis* NZ9000 transformed with the chloramphenicol resistant plasmid pleiss-Nuc	This work

Kp	pH-inducible lactococcin A producing and lactococcin G immunity strain with a GusA3 reporter;<br><br>Contains plasmid pleiss-P_{774}-lcnA-P_{774}-lagC-GusA3	This work
**Plasmids**		
pleiss-Nuc	Plasmid used for cloning in this work; chloramphenicol resistant	(6)
pleiss-lcnG	Plasmid for lcnG expression; Contains the whole gene cluster of lcnG	This work
pleiss-Cα_0	Plasmid for α peptide expression; Contains *lagA*, *lagC*, *lagD* and *lagE*	This work
pleiss-Cβ_0	Plasmid for β peptide expression; Contains *lagB*, *lagC*, *lagD* and *lagE*	This work
pleiss-Cα	Plasmid for α peptide and GFP expression; Contains *lagA*, *lagC*, *lagD*, *lagE* and *yemGFP*	This work
pleiss-Cβ	Plasmid for β peptide and RFP expression; Contains *lagA*, *lagC*, *lagD*, *lagE* and *mCherry*	This work
pleiss-Cβ’	Plasmid for β peptide-free variant; Contains *lagC*, *lagD*, *lagE* and *mCherry*	This work
pleiss-Cα_0-$P_4\alpha$	Plasmid for increased α peptide expression; Contains an additional copy of α peptide under the control of P4 promoter	This work
pleiss-C$\beta_{0v1/3}$	Plasmid for reduced β peptide expression; Contains 6 AT in the RBS spacer region of β peptide	This work
pleiss-C$\beta_{0v1/5}$	Plasmid for reduced β peptide expression; Contains 8 AT in the RBS spacer region of β peptide	This work
pleiss-lcnA-GusA3	Plasmid for lcnA and GusA3 reporter expression; Contains the whole gene cluster of lcnA and a *gusA3* gene	This work
pleiss-lG-GusA3	Plasmid with a *lagC* gene and a *gusA3* gene	This work

pleiss-P_{774}-lcnA-gusA3	Plasmid for pH-inducible lcnA and constitutive GusA3 reporter expression; Contains the whole gene cluster of lcnA and a *gusA3* gene	This work
pleiss-P_{774}-lcnA-P_{774}-lagC-gusA3	Plasmid for pH-inducible lcnA, lcnG-resistance and constitutive GusA3 reporter expression; Contains the whole gene cluster of lcnA and lcnG immunity gene *lagC* and a *gusA3* gene	This work

Supplementary Table 2: Primers used in this study.

Name	Sequence (5' to 3')	Description
Construction of pleiss-lcnG		
P_g-F	catagtcaaattccccataattttgagtaataaaaaccttcttcaactaacggggcagg	Amplify pleiss backbone
P_g-R	gcagtttatcacatgtaacaatttacaaaatgctcattgtaaaagtgtcactgctgctag	
lcnG-F	tactcaaaaattatggggaatttgactatg	Amplify lactococcin G gene cluster
lcnG-R	ttttgtaaattgttacatgtgataaactgc	
Construction of pleiss-$C\alpha_0$		
$P_{C\alpha 0}$-F	ttagtgtttctttttctattaattctaga	Amplify pleiss backbone + *lagA*
$P_{C\alpha 0}$-R	gcagtttatcacatgtaacaatttacaaaa	
$C\alpha_0$-F	tctagaattaatagaaaaagaaacactaacgagtaacttttgttagattaaataaggca	Amplify *lagC* + *lagD* + *lagE*
$C\alpha_0$-R	ttttgtaaattgttacatgtgataaactgctaattaaaaactagatatttttagtttttt	
Construction of pleiss-$C\beta_0$		
$P_{C\beta 0}$-F	aaaaattctccttttatttattattagaat	Amplify pleiss backbone + P_{c3}
$P_{C\alpha 0}$-R	gcagtttatcacatgtaacaatttacaaaa	
$C\beta_0$-F	attctaataataaataaaaggagaattttt cgagtaacttttgttagattaaataaggca	Amplify *lagB* + *lagC* + *lagD* + *lagE*
$C\alpha_0$-R	ttttgtaaattgttacatgtgataaactgctaattaaaaactagatatttttagtttttt	
Construction of pleiss-Cα		
$P_{C\alpha}$-F	cattgagaagattgccgaaaatatgcactc	Amplify pleiss backbone + *lagA* + *lagC* + *lagD* + *lagE*
$P_{C\alpha}$-R	agatgctcttcagcatgttcaatgatgtcg	
Cα-F	gagtgcatattttcggcaatcttctcaatgtaactggaataatcaaccaaatagatagtg	Amplify Pcon+GFP
Cα-R	cgacatcattgaacatgctgaagagcatctctagtaatttgcttagccattcttttatta	
Construction of pleiss-Cβ		
$P_{C\beta}$-F	cattgagaagattgccgaaaatatgcactc	Amplify pleiss backbone + *lagB* + *lagC* + *lagD* + *lagE*
$P_{C\beta}$-R	agatgctcttcagcatgttcaatgatgtcg	

Cα-R	cgacatcattgaacatgctgaagagcatctctagtaatttgcttagccattcttttatta	
Construction of pleiss-Cβ’		
$P_{C\beta'}$-F	aaaaattctccttttatttattattagaat	Amplify pleiss backbone+*lagE* +*rfp*
$P_{C\beta'}$-R	atgaatcaaaataattggcaaaatacttca	
Cβ’-F	attctaataataaataaaaggagaatttttttataagacttataatatttagcaaaatat	Amplify *lagC* +*lagD*
Cβ’-R	tgaagtattttgccaattattttgattcat	
Construction of pleiss-$C\alpha_0$-$P_4\alpha$		
$P_{C\alpha0\text{-}P4\alpha}$-F	cattgagaagattgccgaaaatatgcactc	Amplify pleiss backbone + *lagA* + *lagC* + *lagD* + *lagE*
$P_{C\alpha0\text{-}P4\alpha}$-R	agatgctcttcagcatgttcaatgatgtcg	
$C\alpha_0$-$P_{4\alpha}$-F	gagtgcatattttcggcaatcttctcaatgagatctggtccgcgagttgttgacagagtt	Amplify P_4 + *lagA*
$C\alpha_0$-$P_{4\alpha}$-R	cgacatcattgaacatgctgaagagcatct ttagtgtttcttttttctattaattctaga	
Construction of pleiss-$C\beta_{0v1/3}$		
$P_{C\beta0v1/3}$-F	aaaaattctccttttatttattattagaat	Amplify pleiss backbone
$P_{C\beta0v1/3}$-R	gcagtttatcacatgtaacaatttacaaaa	
$C\beta_{0v1/3}$-F	attctaataataaataaaaggagaatttttatatatatatatgaaaaataataataatttttttcaagggt	Amplify 6AT+ *lagB* + *lagC* + *lagD* + *lagE*
$C\beta_{0v1/3}$-R	ttttgtaaattgttacatgtgataaactgc	
Construction of pleiss-$C\beta_{0v1/5}$		
$P_{1/5C\beta0}$-F	aaaaattctccttttatttattattagaat	Amplify pleiss backbone
$P_{1/5C\beta0}$-R	gcagtttatcacatgtaacaatttacaaaa	
$C\beta_{0v1/5}$-F	attctaataataaataaaaggagaatttttatatatatatatatatgaaaaataataataatttttttcaag ggt	Amplify 8AT+ *lagB* + *lagC* + *lagD* + *lagE*
$C\beta_{0v1/5}$-R	ttttgtaaattgttacatgtgataaactgc	
Construction of pleiss-lcnA-GusA3		
$P_{lcnA\text{-}GusA3}$-F	ataaacgctaaaacgtctcagaaacgattt	Amplify lactococcin A and pleiss backbone

$P_{IcnA\text{-}GusA3}$-R	ttcgtttagttatcggcataatcgttaaaa	
IcnA-GusA3-F	aaatcgtttctgagacgttttagcgtttattaactggaataatcaaccaaatagatagtg	Amplify *GusA3* gene
IcnA-GusA3-R	ttttaacgattatgccgataactaaacgaa ttaatttaattgttgccatctctttttcaa	
Construction of pleiss-IG-GusA3		
$P_{IG\text{-}GusA3}$-F	tttgagacgttttaataaaaaatcgacatc	Amplify *lagC* and pleiss backbone
$P_{IG\text{-}GusA3}$-R	atcgtttctgagacgttttagcgtttattt	
GusA3-F	gatgtcgattttttattaaaacgtctcaaataactggaataatcaaccaaatagatagtg	Amplify *GusA3* gene
GusA3-R	aaataaacgctaaaacgtctcagaaacgatttaatttaattgttgccatctctttttcaa	
Construction of pleiss-P_{774}-IcnA-GusA3		
$P_{p774\text{-}lagC}$-F	ataaacgctaaaacgtctcagaaacgattt	Amplify the whole IcnA cluster and *GusA3* and vector backbone
$P_{p774\text{-}lagC}$-R	tttttcgtttagttatcggcataatcgtt	
P_{774}-lagC-F	aaatcgtttctgagacgttttagcgtttatatttttggttgccatttgttaacgctgtgt	Amplify P_{774} + *lagC*
P_{774}-lagC-R	aacgattatgccgataactaaacgaaaaaatcacctaataaataattgatttatgattat	
Construction of pleiss-P_{774}-IcnA-P_{774}-lagC-GusA3		
$P_{p774\text{-}IcnA}$-F	ctattttattataccagccccctcactacacagcgttaacaaatggcaaccaaaaatcagtaagtaa tattattttcatt	Amplify part IcnA cluster and *GusA3* and vector backbone
$P_{p774\text{-}IcnA}$-R	gtcgacctcgagtgcatattttcggcaatc	
P_{774}-IcnA-F	tgtagtgagggggctggtataataaaataggtaaaaaaatattcggaggaattttgaaat	Amplify P_{774} + *IcnA* gene
P_{774}-IcnA-R	gattgccgaaaatatgcactcgaggtcgac	

Supplementary Table 3: Supplementary Sequences.

>lcnG gene cluster in pleiss-lcnG

cgttagttgaagaaggtttttatattacagctccagatctatactcaaaaattatggggaatttgactatgactacgttagtaaactaattgtagaagaaaaaataa
cagaaaataccgtcaagcgtcgagcaagtacggtctgttcatggataaattggatattatcggttgtggagtagatattttaatattgaaaagtatattgtgttgac
tattcgaacaataataagatttataaataaatttattaaataaaaaatcactggcaataatatctagtgatttttttatttagtgaattttgatatataaatatgatttaatac
tttcgtagaggttaaagaatagtaactatgttgcctatacaaaaattcaaggagatgtctccacaaatatggaaaggcgcaaaaaatatctctttatcaagcattt
attttaattatttccatcgcaatagtaagattggcacttaataaaaaataatactcgatttttagaataaaattaaatatgatattgatttatgttactaaatatgatataa
tattctaataataaataaaaggagaatttttatgaaagaattatcagaaaaagaattacgagaatgcgttggcggtggaacttgggatgatattggtcaaggaat
aggaagagtcgcttattgggttggaaaagccatgggaaatatgagcgatgttaatcaagcttctagaattaatagaaaaaagaaacactaatttataagactta
taatatttagcaaaatatggagagtgaagtatatgaaaaataataataattttttcaagggtatggaaataattgaagatcaagaattagtttcaataactggagg
gaaaaaatggggctggctagcttgggtagacccagcttatgaatttatcaagggctttggtaaaggtgcaattaaagaaggaaataaagataagtggaaaaa
tatctgacgagtaacttttgttagattaaataaggcaagtagtttagttaagctatttgcttttattatggttgaagaatttacaaaaagaagggataacaatttttgttt
aataatatagtagtttttataaatttttttaagctttgtatttattcttgtaggtgtagatattaaatataatgacaatcggataaagatagtacatgttactttttttattagtt
tcattttagtaatgctaacaagtttaatatcacataatagcattgcatatagcttatcacaaattttggaaattttatgtataatttgtattttgcttttgttttatattttgaa
aaaaactaactcactaagtaatagagctaatgtggtatttattatttttatcgttactcaagttattataatcataaatcaattatttattaggtgattttatgaaaaaaat
aatatatcaacaggatgaaaaagattgtggagtagcttgtatagccatgattttaaaacattatggtaccgaaattactattcaaaggttgcgtgaactttctggg
acagatttagatggcacgtctgcttttggaataaaaaaaaacatttgaaaaattaggatttgatgcaccagcattcaaagctggtgatgaaacatggcaagaaaa
agatataccccttgcctttgatagctcacataataagtgaacaaaagtatcaacactacgtagtggtttataaagttaaaggtgatgagatttggattgctgaccca
gcaaagggaaagattagaaaaactatttctgaattttctaaagagtggacaggtgtcttactttttcctaaaccaaaagcagaatacaaaccgtctattgaaaga
gtagatagtttatcaacgttctttcctatactaataaaacagaagtcactcttcatcacgatttttggaatcttaagttcttactattttcaaggcttattggataatatta
ttccaaatcaggctcggtcgactttaaatattctttccataggacttatttttgtttatctttttcgtgtgctttttgaatatagtcgtagctatcttctacttttaattgggc
aaagaatgagtatgagcataatgcttggttattttaaacacgttttgtcactacctctgagtttctttgcaaccagaaagtcaggagaaattatttctcgcttcttgg
atgctaataaaattattgatgctcttgctagtgccaccttatctttaattctggatattggaatggtaattctggttggcacgacacttgcgattcaaagtactcagct
ttttttgctcacccttgcctttctaccttttttatattttggtagtatatgtgtttattaggagttacgataaagcaaatacagaagaaatgagtgcaggagctgaagtta
attctagtattattgaaagtctaaaaggaattgaaactattaaatcttacaatggagaaaatcatgtctatgatcgtgtagattcagaatttgtaactttaatgaaaaa
gtcttttaaatcggtcacacttgataatgtacaacagagtttaaaaatggttattgaacttataagtagtgtattgatactatggctagggtcaagttatgttatagat
ggaaaaataagtctaggacaattaattacctataatgctttacttgtattttttactgaacctttacaaaatattattaatttgcaagtgaaaatgcaaaaagcacgtg
tagcaaataaacgtttgaacgaaatcatgtcaatatctccagaacaaagaaatacgaatattaatatatcgaaaaatatatttaataaggatataaaattagataa
agtaagtttttcttataatatgaagcttcccgttttaagagatgtttctttagaaatatattccaaaagtaaggttgctcttgttggtgtgagcggttcaggcaagtcta
cactagctaaactattagtaaaattctatgatccctctgagggaaatatcacttatggtgatataaattgtcaagatattgaaaatcataaattaagaaatcatgtta
cttatgttcctcaggaatctttctttttttaatggtacaattatagataatttaacttttggtcttagtcatcaaccagagtttgaaaaaatttttagagcatgtaaagctgc
ttgtcttgttgattttattaaccaacaacctttaagatttgattcagttcttgaagaaggaggaaataatctatcaggaggacaaaagcaacgcttagcaatagcta
gagctattttaaatgattctgaaataattatttttgatgaagcaactagtggtcttgataccttattagaaaaagagattttagaatatttaattaagttacaggataaa
actatcatttttattgcccaccatctatcaatagctaaagcctgtgatgaaatcattgttctagatcaaggaatattggttgggagaggaacacacgaagaattat
ctgaaaaagagggtgtatataggagattattaaacgcataacaacttacggagtaatacaaacatgaatcaaaataattggcaaaatacttcacagttctatagt
caaagtcacaaacatttctatcgctggattatttatcctacagtagttttatttctttttattcttcttttttttattcttcgggaagaaagaaattattattaaatcccaagca
caaattacatcagatttagtttctaaaatacagattccaattgaatctaaaataatagaaaataaactttctgaaaatttgtctgtgaaaaaaggggaagcattagtt
aagcttgatatagagtctcttaaacaacaaaaaagcagtttagaaaaggaaattcctttagttcaaaaccaaattttagctaatcaaactttcattgatagtctgaa
gcaaggaaaaagtttgttcacttcagatgatgattttggttatagtaatcaattgcaaagcttactatcggagaatactgcttctgattatgcatttaaacaaagcat
aattgatcatgagagtgaaaaaagtacttatcaaaattctaaaaattcaattcagaaaagcatctctaaaaaacaagtagaaaaaaatgattgggaaaaaataa
aaagtgcttggagttctcagtcaaatctttcgggggcttcagaagatactacttcacaatatttgaattttcaagaacaactaaaggctagttcaaaagaagaaa
aagaacaagtaaaggtaactatttcttctagtattaatgataaaatttcacaaattgaccaggaattggaacagttagaaatggagcagtcaaaactaacacctc
cagcttcttacgataatgagaaaagtagtcaggaatataaaaagaaacagcttgttgaacaaactattgcaacggcaaagcaaaaaagaatagaattcaaag
aagcgcaagaaaaatataatttagaacttcaagaagtcaataagcaaatacaagatgaaataattacctcacctattgatggttttgttcatattaattataatgtc
aaagatcagaagattatccctaagggagaagttattgccgaaatttatccagaaattaaaccagggaaaattgaatttacttcccaaatcgaagcatccgactt
aacacaagttaaatcagggatgcgggttcattttaaacttgattcaaagggaaatagccctatcataatggagggaaaaatcaaagaaatctctgcgaatgct
gaaacttcggagcgaggaagcttttatgttgtaaaagggttcttaaagcagacaaataaaactccttttaatagtcgatatggcttaaacggtcgattatcactta
ttgttggtaaaaaatcgtattttaatgttttaaaagaaatgattataaaaaactaaaaatatctagtttttaattagcagtttatcacatgtaacaatttacaaaa

lagA: 577-741; *lagB*: 787-969; *lagC*: 1068-1400; *lagD*: 1405-3516; *lagE*: 3539-4909

>lcnG gene cluster in pleiss-$C\alpha_0$

cgttagttgaagaaggtttttatattacagctccagatctaTactcaaaaattatggggaatttgactatgactacgttagtaaactaattgtagaagaaaaaata
acagaaaataccgtcaagcgtcgagcaagtacggtctgttcatggataaattggatattatcggttgtggagtagatattttaatattgaaaagtatattgtgttga
ctattcgaacaataataagatttataaataaatttattaaataaaaaatcactggcaataatatctagtgatttttttatttagtgaattttgatatataaatatgatttaata
ctttcgtagaggttaaagaatagtaactatgttgcctatacaaaaattcaaggagatgtctccacaaatatggaaaggcgcaaaaaaatatctctttatcaagcatt
tattttaattatttccatcgcaatagtaagattggcacttaataaaaaataatactcgatttttagaataaaattaaatatgatattgatttatgttactaaatatgatata
atattctaataataaataaaaggagaatttttatgaaagaattatcagaaaaagaattacgagaatgcgttggcggtggaacttgggatgatattggtcaaggaa
taggaagagtcgcttattgggttggaaaagccatgggaaatatgagcgatgttaatcaagcttctagaattaatagaaaaaagaaacactaacgagtaactttt
gttagattaaataaggcaagtagtttagttaagctatttgcttttattatggttgaagaatttacaaaaagaagggataacaatttttgtttaataatatagtagtttttat
aaattttttaagctttgtatttattcttgtaggtgtagatattaaatataatgacaatcggataaagatagtacatgttactttttttattagtttcattttagtaatgctaac
aagtttaatatcacataatagcattgcatatagcttatcacaaattttggaaattttatgtataatttgtattttgcttttgttttatattttgaaaaaaactaactcactaag
taatagagctaatgtggtatttattatttttatcgttactcaagttattataatcataaatcaattatttattaggtgattttatgaaaaaaataatatatcaacaggatga
aaaagattgtggagtagcttgtatagccatgattttaaaacattatggtaccgaaattactattcaaaggttgcgtgaactttctgggacagatttagatggcacg
tctgcttttggaataaaaaaaacatttgaaaaattaggatttgatgcaccagcattcaaagctggtgatgaaacatggcaagaaaaagatatacccttgcctttg
atagctcacataataagtgaacaaaagtatcaacactacgtagtggtttataaagttaaaggtgatgagatttggattgctgacccagcaaagggaaagattag
aaaaactatttctgaattttctaaagagtggacaggtgtcttactttttcctaaaccaaaagcagaatacaaaccgtctattgaaagagtagatagtttatcaacgt
tctttcctatactaataaaacagaagtcactcttcatcacgatttttggaatcttaagttcttactattttcaaggcttattggataatattattccaaatcaggctcggt
cgactttaaatattctttccataggacttatttttgtttatctttttcgtgtgctttttgaatatagtcgtagctatcttctacttttaattgggcaaagaatgagtatgagca
taatgcttggttattttaaacacgttttgtcactacctctgagtttctttgcaaccagaaagtcaggagaaattatttctcgcttcttggatgctaataaaattattgatg
ctcttgctagtgccaccttatctttaattctggatattggaatggtaattctggttggcacgacacttgcgattcaaagtactcagcttttttttgctcacccttgccttt
ctacctttttatattttggtagtatatgtgtttattaggagttacgataaagcaaatacagaagaaatgagtgcaggagctgaagttaattctagtattattgaaagtc
taaaaggaattgaaactattaaatcttacaatggagaaaatcatgtctatgatcgtgtagattcagaatttgtaactttaatgaaaaagtcttttaaatcggtcacac
ttgataatgtacaacagagtttaaaaatggttattgaacttataagtagtgtattgatactatggctagggtcaagttatgttatagatggaaaaataagtctagga
caattaattacctataatgctttacttgtatttttttactgaacctttacaaaatattattaatttgcaagtgaaaatgcaaaaagcacgtgtagcaaataaacgtttgaa
cgaaatcatgtcaatatctccagaacaaagaaatacgaatattaatatatcgaaaaatatatttaataaggatataaaattagataaagtaagtttttcttataatat
gaagcttcccgttttaagagatgtttctttagaaatatattccaaaagtaaggttgctcttgttggtgtgagcggttcaggcaagtctacactagctaaactattagt
aaaattctatgatccctctgagggaaatatcacttatggtgatataaattgtcaagatattgaaaatcataaattaagaaatcatgttacttatgttcctcaggaatct
ttcttttttaatggtacaattatagataatttaacttttggtcttagtcatcaaccagagtttgaaaaaatttttagagcatgtaaagctgcttgtcttgttgattttattaa
ccaacaacctttaagatttgattcagttcttgaagaaggaggaaataatctatcaggaggacaaaagcaacgcttagcaatagctagagctattttaaatgattc
tgaaataattatttttgatgaagcaactagtggtcttgataccttattagaaaaagagattttagaatatttaattaagttacaggataaaactatcatttttattgccca
ccatctatcaatagctaaagcctgtgatgaaatcattgttctagatcaaggaatattggttgggagaggaacacacgaagaattatctgaaaaagagggtgtat
ataggagattattaaacgcataacaacttacggagtaatacaaacatgaatcaaaataattggcaaaatacttcacagttctatagtcaaagtcacaaacatttct
atcgctggattatttatcctacagtagttttatttctttttattcttcttttttttattcttcgggaagaaagaaattattattaaatcccaagcacaaattacatcagatttag
tttctaaaatacagattccaattgaatctaaaataatagaaaataaactttctgaaaatttgtctgtgaaaaaaggggaagcattagttaagcttgatatagagtct
cttaaacaacaaaaaaagcagtttagaaaaggaaattcctttagttcaaaaccaaattttagctaatcaaactttcattgatagtctgaagcaaggaaaaagtttgtt
cacttcagatgatgattttggttatagtaatcaattgcaaagcttactatcggagaatactgcttctgattatgcatttaaacaaagcataattgatcatgagagtga
aaaaagtacttatcaaaattctaaaaattcaattcagaaaagcatctctaaaaaacaagtagaaaaaaatgattgggaaaaaataaaaagtgcttggagttctc
agtcaaatctttcgggggcttcagaagatactacttcacaatatttgaattttcaagaacaactaaaggctagttcaaaagaagaaaaagaacaagtaaaggta
actatttcttctagtattaatgataaaatttcacaaattgaccaggaattggaacagttagaaatggagcagtcaaaactaacacctccagcttcttacgataatg
agaaaagtagtcaggaatataaaaagaaacagcttgttgaacaaactattgcaacggcaaagcaaaaaagaatagaattcaaagaagcgcaagaaaaata
taatttagaacttcaagaagtcaataagcaaatacaagatgaaataattacctcacctattgatggttttgttcatattaattataatgtcaaagatcagaagattatc
cctaagggagaagttattgccgaaatttatccagaaattaaaccagggaaaattgaatttacttcccaaatcgaagcatccgacttaacacaagttaaatcagg
gatgcgggttcattttaaacttgattcaaagggaaatagccctatcataatggagggaaaaatcaaagaaatctctgcgaatgctgaaacttcggagcgagga
agcttttatgttgtaaaagggttcttaaagcagacaaataaaactccttttaatagtcgatatggcttaaacggtcgattatcacttattgttggtaaaaaatcgtatt
ttaatgttttaaaagaaatgattataaaaaactaaaaatatctagtttttaattagcagtttatcacatgtaacaatttacaaaa

lagA: 577-741; *lagC*: 840-1172; *lagD*: 1177-3288; *lagE*: 3311-4681

>lcnG gene cluster in pleiss-$C\beta_0$

cgttagttgaagaaggtttttatattacagctccagatctaTactcaaaaattatggggaatttgactatgactacgttagtaaactaattgtagaagaaaaaata
acagaaaataccgtcaagcgtcgagcaagtacggtctgttcatggataaattggatattatcggttgtggagtagatattttaatattgaaaagtatattgtgttga
ctattcgaacaataataagatttataaataaatttattaaataaaaaatcactggcaataatatctagtgatttttttatttagtgaattttgatatataaatatgatttaata
ctttcgtagaggttaaagaatagtaactatgttgcctatacaaaaattcaaggagatgtctccacaaatatggaaaggcgcaaaaaatatctctttatcaagcatt
tattttaattatttccatcgcaatagtaagattggcacttaataaaaaataatactcgatttttagaataaaattaaatatgatattgatttatgttactaaatatgatata
atattctaataataaataaaaggagaatttttatgaaaaataataataatttttcaagggtatggaaataattgaagatcaagaattagtttcaataactggaggga
aaaaatggggctggctagcttgggtagacccagcttatgaatttatcaagggctttggtaaaggtgcaattaaagaaggaaataaagataagtggaaaaatat
ctgacgagtaacttttgttagattaaataaggcaagtagtttagttaagctatttgcttttattatggttgaagaatttacaaaaagaagggataacaatttttgtttaa
taatatagtagtttttataaatttttaagctttgtatttattcttgtaggtgtagatattaaatataatgacaatcggataaagatagtacatgttactttttttattagtttc
attttagtaatgctaacaagtttaatatcacataatagcattgcatatagcttatcacaaattttggaaattttatgtataatttgtattttgcttttgttttatattttgaaaa
aaactaactcactaagtaatagagctaatgtggtatttattatttttatcgttactcaagttattataatcataaatcaattatttattaggtgattttatgaaaaaaataat
atatcaacaggatgaaaaagattgtggagtagcttgtatagccatgattttaaaacattatggtaccgaaattactattcaaaggttgcgtgaactttctgggaca
gatttagatggcacgtctgcttttggaataaaaaaaacatttgaaaaattaggatttgatgcaccagcattcaaagctggtgatgaaacatggcaagaaaaaga
tatacccttgcctttgatagctcacataataagtgaacaaaagtatcaacactacgtagtggtttataaagttaaaggtgatgagatttggattgctgacccagca
aagggaaagattagaaaaactatttctgaattttctaaagagtggacaggtgtcttactttttcctaaaccaaaagcagaatacaaaccgtctattgaaagagta
gatagtttatcaacgttctttcctatactaataaaacagaagtcactcttcatcacgatttttggaatcttaagttcttactattttcaaggcttattggataatattattc
caaatcaggctcggtcgactttaaatattctttccataggacttatttttgtttatctttttcgtgtgctttttgaatatagtcgtagctatcttctacttttaattgggcaaa
gaatgagtatgagcataatgcttggttattttaaacacgttttgtcactacctctgagtttctttgcaaccagaaagtcaggagaaattatttctcgcttcttggatgc
taataaaattattgatgctcttgctagtgccaccttatctttaattctggatattggaatggtaattctggttggcacgacacttgcgattcaaagtactcagcttttttt
gctcacccttgcctttctaccttttttatattttggtagtatatgtgtttattaggagttacgataaagcaaatacagaagaaatgagtgcaggagctgaagttaattct
agtattattgaaagtctaaaaggaattgaaactattaaatcttacaatggagaaaatcatgtctatgatcgtgtagattcagaatttgtaactttaatgaaaaagtct
tttaaatcggtcacacttgataatgtacaacagagtttaaaaatggttattgaacttataagtagtgtattgatactatggctagggtcaagttatgttatagatgga
aaaataagtctaggacaattaattacctataatgctttacttgtattttttactgaacctttacaaaatattattaatttgcaagtgaaaatgcaaaaagcacgtgtag
caaataaacgtttgaacgaaatcatgtcaatatctccagaacaaagaaatacgaatattaatatatcgaaaaatatatttaataaggatataaaattagataaagt
aagtttttcttataatatgaagcttcccgttttaagagatgtttctttagaaatatattccaaaagtaaggttgctcttgttggtgtgagcggttcaggcaagtctaca
ctagctaaactattagtaaaattctatgatccctctgagggaaatatcacttatggtgatataaattgtcaagatattgaaaatcataaattaagaaatcatgttactt
atgttcctcaggaatctttctttttaatggtacaattatagataatttaacttttggtcttagtcatcaaccagagtttgaaaaaatttttagagcatgtaaagctgctt
gtcttgttgattttattaaccaacaacctttaagatttgattcagttcttgaagaaggaggaaataatctatcaggaggacaaaagcaacgcttagcaatagctag
agctattttaaatgattctgaaataattatttttgatgaagcaactagtggtcttgataccttattagaaaaagagattttagaatatttaattaagttacaggataaaa
ctatcattttttattgcccaccatctatcaatagctaaagcctgtgatgaaatcattgttctagatcaaggaatattggttgggagaggaacacacgaagaattatct
gaaaaagagggtgtatataggagattattaaacgcataacaacttacggagtaatacaaacatgaatcaaaataattggcaaaatacttcacagttctatagtc
aaagtcacaaacatttctatcgctggattatttatcctacagtagttttatttctttttattcttctttttttattcttcgggaagaaagaaattattattaaatcccaagcac
aaattacatcagatttagtttctaaaatacagattccaattgaatctaaaataatagaaaataaactttctgaaaatttgtctgtgaaaaaaggggaagcattagtta
agcttgatatagagtctcttaaacaacaaaaaagcagtttagaaaaggaaattcctttagttcaaaaccaaattttagctaatcaaactttcattgatagtctgaag
caaggaaaaagtttgttcacttcagatgatgattttggttatagtaatcaattgcaaagcttactatcggagaatactgcttctgattatgcatttaaacaaagcata
attgatcatgagagtgaaaaaagtacttatcaaaattctaaaaattcaattcagaaaagcatctctaaaaaacaagtagaaaaaaatgattgggaaaaaataaa
aagtgcttggagttctcagtcaaatctttcgggggcttcagaagatactacttcacaatatttgaattttcaagaacaactaaaggctagttcaaaagaagaaaa
agaacaagtaaaggtaactatttcttctagtattaatgataaaatttcacaaattgaccaggaattggaacagttagaaatggagcagtcaaaactaacacctcc
agcttcttacgataatgagaaaagtagtcaggaatataaaaagaaacagcttgttgaacaaactattgcaacggcaaagcaaaaaagaatagaattcaaaga
agcgcaagaaaaatataatttagaacttcaagaagtcaataagcaaatacaagatgaaataattacctcacctattgatggttttgttcatattaattataatgtca
aagatcagaagattatccctaagggagaagttattgccgaaatttatccagaaattaaaccagggaaaattgaatttacttcccaaatcgaagcatccgactta
acacaagttaaatcagggatgcgggttcattttaaacttgattcaaagggaaatagccctatcataatggagggaaaaatcaaagaaatctctgcgaatgctg
aaacttcggagcgaggaagcttttatgttgtaaaagggttcttaaagcagacaaataaaactcctttttaatagtcgatatggcttaaacggtcgattatcacttatt
gttggtaaaaaatcgtattttaatgttttaaaagaaatgattataaaaaactaaaaatatctagtttttaattagcagtttatcacatgtaacaatttacaaaa

lagB: 577-759; *lagC*: 858-1190; *lagD*: 1195-3306; *lagE*: 3329-4699

>lcnG gene cluster and gfp reporter in pleiss-Cα
cgttagttgaagaaggtttttatattacagctccagatctaTactcaaaaattatggggaatttgactatgactacgttagtaaactaattgtagaagaaaaaata
acagaaaataccgtcaagcgtcgagcaagtacggtctgttcatggataaattggatattatcggttgtggagtagatattttaatattgaaaagtatattgtgttga
ctattcgaacaataataagatttataaataaatttattaaataaaaaatcactggcaataatatctagtgatttttatttagtgaattttgatatataaatatgatttaata
ctttcgtagaggttaaagaatagtaactatgttgcctatacaaaaattcaaggagatgtctccacaaatatggaaaggcgcaaaaaatatctctttatcaagcatt
tattttaattatttccatcgcaatagtaagattggcacttaataaaaaataatactcgatttttagaataaaattaaatatgatattgatttatgttactaaatatgatata
atattctaataataaataaaaggagaatttttatgaaagaattatcagaaaaagaattacgagaatgcgttggcggtggaacttgggatgatattggtcaaggaa
taggaagagtcgcttattgggttggaaaagccatgggaaatatgagcgatgttaatcaagcttctagaattaatagaaaaaagaaacactaacgagtaactttt
gttagattaaataaggcaagtagtttagttaagctatttgcttttattatggttgaagaatttacaaaaagaagggataacaattttgtttaataatatagtagtttttat
aaattttttaagctttgtatttattcttgtaggtgtagatattaaatataatgacaatcggataaagatagtacatgttactttttttattagtttcattttagtaatgctaac
aagtttaatatcacataatagcattgcatatagcttatcacaaattttggaaattttatgtataatttgtattttgcttttgttttatattttgaaaaaaactaactcactaag
taatagagctaatgtggtatttattatttttatcgttactcaagttattataatcataaatcaattatttattaggtgattttatgaaaaaaataatatatcaacaggatga
aaaagattgtggagtagcttgtatagccatgattttaaaacattatggtaccgaaattactattcaaaggttgcgtgaactttctgggacagatttagatggcacg
tctgcttttggaataaaaaaaacatttgaaaaattaggatttgatgcaccagcattcaaagctggtgatgaaacatggcaagaaaaagatatacccttgcctttg
atagctcacataataagtgaacaaaagtatcaacactacgtagtggtttataaagttaaaggtgatgagatttggattgctgacccagcaaagggaaagattag
aaaaactatttctgaattttctaaagagtggacaggtgtcttactttttcctaaaccaaaagcagaatacaaaccgtctattgaaagagtagatagtttatcaacgt
tctttcctatactaataaaacagaagtcactcttcatcacgatttttggaatcttaagttcttactattttcaaggcttattggataatattattccaaatcaggctcggt
cgactttaaatattctttccataggacttatttttgtttatctttttcgtgtgctttttgaatatagtcgtagctatcttctacttttaattgggcaaagaatgagtatgagca
taatgcttggttattttaaacacgttttgtcactacctctgagtttctttgcaaccagaaagtcaggagaaattatttctcgcttcttggatgctaataaaattattgatg
ctcttgctagtgccaccttatctttaattctggatattggaatggtaattctggttggcacgacacttgcgattcaaagtactcagctttttttgctcacccttgccttt
ctaccttttatattttggtagtatatgtgtttattaggagttacgataaagcaaatacagaagaaatgagtgcaggagctgaagttaattctagtattattgaaagtc
taaaaggaattgaaactattaaatcttacaatggagaaaatcatgtctatgatcgtgtagattcagaatttgtaactttaatgaaaaagtcttttaaatcggtcacac
ttgataatgtacaacagagtttaaaaatggttattgaacttataagtagtgtattgatactatggctagggtcaagttatgttatagatggaaaaataagtctagga
caattaattacctataatgctttacttgtattttttactgaacctttacaaaatattattaatttgcaagtgaaaatgcaaaaagcacgtgtagcaaataaacgtttgaa
cgaaatcatgtcaatatctccagaacaaagaaatacgaatattaatatatcgaaaaatatatttaataaggatataaaattagataaagtaagtttttcttataatat
gaagcttcccgttttaagagatgtttctttagaaatatattccaaaagtaaggttgctcttgttggtgtgagcggttcaggcaagtctacactagctaaactattagt
aaaattctatgatccctctgagggaaatatcacttatggtgatataaattgtcaagatattgaaaatcataaattaagaaatcatgttacttatgttcctcaggaatct
ttctttttaatggtacaattatagataatttaacttttggtcttagtcatcaaccagagtttgaaaaaatttttagagcatgtaaagctgcttgtcttgttgattttattaa
ccaacaacctttaagatttgattcagttcttgaagaaggaggaaataatctatcaggaggacaaaagcaacgcttagcaatagctagagctattttaaatgattc
tgaaataattatttttgatgaagcaactagtggtcttgataccttattagaaaaagagattttagaatatttaattaagttacaggataaaactatcatttttattgccca
ccatctatcaatagctaaagcctgtgatgaaatcattgttctagatcaaggaatattggttgggagaggaacacacgaagaattatctgaaaaagagggtgtat
ataggagattattaaacgcataacaacttacggagtaatacaaacatgaatcaaaataattggcaaaatacttcacagttctatagtcaaagtcacaaacatttct
atcgctggattatttatcctacagtagttttatttctttttattcttctttttttattcttcgggaagaaagaaattattattaaatcccaagcacaaattacatcagatttag
tttctaaaatacagattccaattgaatctaaaataatagaaaataaactttctgaaaatttgtctgtgaaaaaaggggaagcattagttaagcttgatatagagtct
cttaaacaacaaaaaaagcagtttagaaaaggaaattcctttagttcaaaaccaaattttagctaatcaaactttcattgatagtctgaagcaaggaaaaagtttgtt
cacttcagatgatgattttggttatagtaatcaattgcaaagcttactatcggagaatactgcttctgattatgcatttaaacaaagcataattgatcatgagagtga
aaaaagtacttatcaaaattctaaaaattcaattcagaaaagcatctctaaaaaacaagtagaaaaaaatgattgggaaaaaataaaaagtgcttggagttctc
agtcaaatctttcgggggcttcagaagatactacttcacaatatttgaattttcaagaacaactaaaggctagttcaaaagaagaaaaagaacaagtaaaggta
actatttcttctagtattaatgataaaatttcacaaattgaccaggaattggaacagttagaaatggagcagtcaaaactaacacctccagcttcttacgataatg
agaaaagtagtcaggaatataaaaagaaacagcttgttgaacaaactattgcaacggcaaagcaaaaaagaatagaattcaaagaagcgcaagaaaaata
taatttagaacttcaagaagtcaataagcaaatacaagatgaaataattacctcacctattgatggttttgttcatattaattataatgtcaaagatcagaagattatc
cctaagggagaagttattgccgaaatttatccagaaattaaaccagggaaaattgaatttacttcccaaatcgaagcatccgacttaacacaagttaaatcagg
gatgcgggttcattttaaacttgattcaaagggaaatagccctatcataatggagggaaaaatcaaagaaatctctgcgaatgctgaaacttcggagcgagga
agcttttatgttgtaaaagggttcttaaagcagacaaataaaactccttttaatagtcgatatggcttaaacggtcgattatcacttattgttggtaaaaaatcgtatt
ttaatgttttaaaagaaatgattataaaaaactaaaaatatctagtttttaattagcagtttatcacatgtaacaatttacaaaaTgctcattgtaaaagtgtcactgct
gctagtggcacttttataatttttagatcgaattcgatatcaagcttatcgataccgtcgacctcgagtgcatattttaactggaataatcaaccaaatagatagtgc
tcccacagcttttaaagagggaaatgcttataaagtttctgcgacaaccactatcaatgcaaaagacctcccaaatatccgatatggtcttcaagggaaaacag
taaccattataggaaagaaaacttatttcaattactttttagataaaataatgggaagaggcaatcagtagagttattaacatttgttaacgagttttatttttatataat
ctataatagatttataaaaataaggagattattatgcgtaaaggcgaagagctgttcactggtgtcgtccctattctggtggaactggatggtgatgtcaacggt
cataagttttccgtgcgtggcgagggtgaaggtgacgcaactaatggtaaactgacgctgaagttcatctgtactactggtaaactgccggtaccttggccga
ctctggtaacgacgctgacttatggtgttcagtgctttgctcgttatccggaccatatgaagcagcatgacttcttcaagtccgccatgccggaaggctatgtgc
aggaacgcacgatttcctttaaggatgacggcacgtacaaaacgcgtgcggaagtgaaatttgaaggcgataccctggtaaaccgcattgagctgaaagg
cattgactttaaagaagacggcaatatcctgggccataagctggaatacaattttaacagccacaatgtttacatcaccgccgataaacaaaaaaatggcatta
aagcgaattttaaaattcgccacaacgtggaggatggcagcgtgcagctggctgatcactaccagcaaaacactccaatcggtgatggtcctgttctgctgc
cagacaatcactatctgagcacgcaaagcgttctgtctaaagatccgaacgagaaacgcgatcatatggttctgctggagttcgtaaccgcagcgggcatca
cgcatggtatggatgaactgtacaaatgatgaaactgctgctgggattacacatggcatggatgagtaa

lagA: 577-741; *lagC*: 840-1171; *lagD*: 1177-3288; *lagE*: 3311-4681; *gfp*: 5112-5831

>lcnG gene cluster and rfp reporter in pleiss-Cβ
cgttagttgaagaaggtttttatattacagctccagatctaTactcaaaaattatggggaatttgactatgactacgttagtaaactaattgtagaagaaaaaata
acagaaaataccgtcaagcgtcgagcaagtacggtctgttcatggataaattggatattatcggttgtggagtagatattttaatattgaaaagtatattgtgttga
ctattcgaacaataataagatttataaataaatttattaaataaaaaatcactggcaataatatctagtgatttttttatttagtgaattttgatatataaatatgatttaata
ctttcgtagaggttaaagaatagtaactatgttgcctatacaaaaattcaaggagatgtctccacaaatatggaaaggcgcaaaaaaatatctctttatcaagcatt
tattttaattatttccatcgcaatagtaagattggcacttaataaaaaaataatactcgatttttagaataaaattaaatatgatattgatttatgttactaaatatgatata
atattctaataataaataaaaggagaatttttAtgaaaaataataataatttttttcaagggtatggaaataattgaagatcaagaattagtttcaataactggaggg
aaaaaatggggctggctagcttgggtagacccagcttatgaatttatcaagggctttggtaaaggtgcaattaaagaaggaaataaagataagtggaaaaat
atctgacgagtaacttttgttagattaaataaggcaagtagtttagttaagctatttgcttttattatggttgaagaatttacaaaaagaagggataacaatttttgttt
aataatatagtagtttttataaatttttttaagctttgtatttattcttgtaggtgtagatattaaatataatgacaatcggataaagatagtacatgttactttttttattagtt
tcattttagtaatgctaacaagtttaatatcacataatagcattgcatatagcttatcacaaattttggaaattttatgtataatttgtattttgcttttgttttatattttgaa
aaaaactaactcactaagtaatagagctaatgtggtatttattatttttatcgttactcaagttattataatcataaatcaattatttattaggtgattttatgaaaaaaat
aatatatcaacaggatgaaaaagattgtggagtagcttgtatagccatgattttaaaacattatggtaccgaaattactattcaaaggttgcgtgaactttctggg
acagatttagatggcacgtctgcttttggaataaaaaaaacatttgaaaaattaggatttgatgcaccagcattcaaagctggtgatgaaacatggcaagaaaa
agatataccccttgcctttgatagctcacataataagtgaacaaaagtatcaacactacgtagtggtttataaagttaaaggtgatgagatttggattgctgaccca
gcaaagggaaagattagaaaaactatttctgaattttctaaagagtggacaggtgtcttactttttcctaaaccaaaagcagaatacaaaccgtctattgaaaga
gtagatagtttatcaacgttctttcctatactaataaaacagaagtcactcttcatcacgatttttggaatcttaagttcttactattttcaaggcttattggataatatta
ttccaaatcaggctcggtcgactttaaatattctttccataggacttatttttgtttatctttttcgtgtgctttttgaatatagtcgtagctatcttctacttttaattgggc
aaagaatgagtatgagcataatgcttggttattttaaacacgttttgtcactacctctgagtttctttgcaaccagaaagtcaggagaaattatttctcgcttcttgg
atgctaataaaattattgatgctcttgctagtgccaccttatctttaattctggatattggaatggtaattctggttggcacgacacttgcgattcaaagtactcagct
ttttttgctcacccttgcctttctaccttttttatattttggtagtatatgtgtttattaggagttacgataaagcaaatacagaagaaatgagtgcaggagctgaagtta
attctagtattattgaaagtctaaaaggaattgaaactattaaatcttacaatggagaaaatcatgtctatgatcgtgtagattcagaatttgtaactttaatgaaaaa
gtcttttaaatcggtcacacttgataatgtacaacagagtttaaaaatggttattgaacttataagtagtgtattgatactatggctagggtcaagttatgttatagat
ggaaaaataagtctaggacaattaattacctataatgctttacttgtattttttactgaacctttacaaaatattattaatttgcaagtgaaaatgcaaaaagcacgtg
tagcaaataaacgtttgaacgaaatcatgtcaatatctccagaacaaagaaatacgaatattaatatatcgaaaaatatatttaataaggatataaaattagataa
agtaagtttttcttataatatgaagcttcccgttttaagagatgtttctttagaaatatattccaaaagtaaggttgctcttgttggtgtgagcggttcaggcaagtcta
cactagctaaactattagtaaaattctatgatccctctgagggaaatatcacttatggtgatataaattgtcaagatattgaaaatcataaattaagaaatcatgtta
cttatgttcctcaggaatctttcttttttaatggtacaattatagataatttaacttttggtcttagtcatcaaccagagtttgaaaaaatttttagagcatgtaaagctgc
ttgtcttgttgattttattaaccaacaacctttaagatttgattcagttcttgaagaaggaggaaataatctatcaggaggacaaaagcaacgcttagcaatagcta
gagctattttaaatgattctgaaataattatttttgatgaagcaactagtggtcttgataccttattagaaaaagagattttagaatatttaattaagttacaggataaa
actatcatttttattgcccaccatctatcaatagctaaagcctgtgatgaaatcattgttctagatcaaggaatattggttgggagaggaacacacgaagaattat
ctgaaaaagagggtgtatataggagattattaaacgcataacaacttacggagtaatacaaacatgaatcaaaataattggcaaaatacttcacagttctatagt
caaagtcacaaacatttctatcgctggattatttatcctacagtagttttatttctttttattcttctttttttattcttcgggaagaaagaaattattattaaatcccaagca
caaattacatcagatttagtttctaaaatacagattccaattgaatctaaaataatagaaaataaactttctgaaaatttgtctgtgaaaaaaggggaagcattagtt
aagcttgatatagagtctcttaaacaacaaaaaaagcagtttagaaaaggaaattcctttagttcaaaaccaaattttagctaatcaaactttcattgatagtctgaa
gcaaggaaaaagtttgttcacttcagatgatgattttggttatagtaatcaattgcaaagcttactatcggagaatactgcttctgattatgcatttaaacaaagcat
aattgatcatgagagtgaaaaaagtacttatcaaaattctaaaaattcaattcagaaaagcatctctaaaaaacaagtagaaaaaaatgattgggaaaaaataa
aaagtgcttggagttctcagtcaaatctttcgggggcttcagaagatactacttcacaatatttgaattttcaagaacaactaaaggctagttcaaaagaagaaa
aagaacaagtaaaggtaactatttcttctagtattaatgataaaatttcacaaattgaccaggaattggaacagttagaaatggagcagtcaaaactaacacctc
cagcttcttacgataatgagaaaagtagtcaggaatataaaaagaaacagcttgttgaacaaactattgcaacggcaaagcaaaaaagaatagaattcaaag
aagcgcaagaaaaatataatttagaacttcaagaagtcaataagcaaatacaagatgaaataattacctcacctattgatggttttgttcatattaattataatgtc
aaagatcagaagattatccctaagggagaagttattgccgaaatttatccagaaattaaaccagggaaaattgaatttacttcccaaatcgaagcatccgactt
aacacaagttaaatcagggatgcgggttcattttaaacttgattcaaagggaaatagccctatcataatggagggaaaaatcaaagaaatctctgcgaatgct
gaaacttcggagcgaggaagcttttatgttgtaaaagggttcttaaagcagacaaataaaactccttttaatagtcgatatggcttaaacggtcgattatcactta
ttgttggtaaaaaatcgtattttaatgttttaaaagaaatgattataaaaaactaaaaatatctagtttttaattagcagtttatcacatgtaacaatttacaaaaTgct
cattgtaaaagtgtcactgctgctagtggcacttttataatttttagatcgaattcgatatcaagcttatcgataccgtcgacctcgagtgcatattttaactggaata
atcaaccaaatagatagtgctcccacagcttttaaagagggaaatgcttataaagtttctgcgacaaccactatcaatgcaaaagacctcccaaatatccgata
tggtcttcaagggaaaacagtaaccattataggaaagaaaacttatttcaattactttttagataaaataatgggaagaggcaatcagtagagttattaacatttgt
taacgagttttatttttatataatctataatagatttataaaaataaggagattattatgtcagaattaattaaagaaaatatgcacatgaaattatatatggaaggtac
tgtcaacaatcatcatttcaaatgcacatccgaaggtgaaggtaaaccatatgaaggcacacaaacaatgcgcatcaaagcagttgaaggtggacccctgc
cctttgcgtttgacattctcgcaacgagctttatgtacgggtctaaaacttttatcaatcacacccaaggcattcctgactttttttaaacagtcctttcctgaaggctt
tacctgggaacgtgtaacaacttatgaagatggcggtgtacttacagcaactcaagatacgagtttacaagatggctgtctgatttacaatgttaaaatccgtgg
cgtaaatttcccgagtaacggacccgtaatgcaaaaaaaaaactcttggttgggaagcatcaacagaaaccttatatcctgcggacggtggcttagaaggacg
cgcagacatggcactgaaattagttggaggcggtcatttaatctgcaacctgaaaacaacctatcgttccaaaaaacccgctaaaaaccttaaaatgcctgga
gtatactatgttgatcgtcgcttagaacgtattaaagaagctgataaagaaacctacgttgaacaacatgaagtagccgtagcccgttattgtgaccttccgtcg
aaattaggacatcgttgatgaaactgctgctgggattacacatggcatggatgagtaa

lagB: 577-759; *lagC*: 858-1190; *lagD*: 1195-3306; *lagE*: 3329-4699; *rfp*: 5112-5831

>lcnG gene cluster in pleiss-$C_{\alpha0}$-$P_{4\alpha}$
cgttagttgaagaaggtttttatattacagctccagatctaTactcaaaaattatggggaatttgactatgactacgttagtaaactaattgtagaagaaaaaata
acagaaaataccgtcaagcgtcgagcaagtacggtctgttcatggataaattggatattatcggttgtggagtagatattttaatattgaaaagtatattgtgttga
ctattcgaacaataataagatttataaataaatttattaaataaaaaatcactggcaataatatctagtgatttttttatttagtgaattttgatatataaatatgatttaata
ctttcgtagaggttaaagaatagtaactatgttgcctatacaaaaattcaaggagatgtctccacaaatatggaaaggcgcaaaaaatatctctttatcaagcatt
tattttaattatttccatcgcaatagtaagattggcacttaataaaaaataatactcgatttttagaataaaattaaatatgatattgatttatgttactaaatatgatata
atattctaataataaataaaaggagaatttttatgaaagaattatcagaaaaagaattacgagaatgcgttggcggtggaacttgggatgatattggtcaaggaa
taggaagagtcgcttattgggttggaaaagccatgggaaatatgagcgatgttaatcaagcttctagaattaatagaaaaaagaaacactaacgagtaactttt
gttagattaaataaggcaagtagtttagttaagctatttgcttttattatggttgaagaatttacaaaaagaagggataacaatttttgtttaataatatagtagtttttat
aaattttttaagctttgtatttattcttgtaggtgtagatattaaatataatgacaatcggataaagatagtacatgttactttttttattagtttcattttagtaatgctaac
aagtttaatatcacataatagcattgcatatagcttatcacaaattttggaaattttatgtataatttgtattttgcttttgttttatattttgaaaaaaactaactcactaag
taatagagctaatgtggtatttattatttttatcgttactcaagttattataatcataaatcaattatttattaggtgattttatgaaaaaaataatatatcaacaggatga
aaaagattgtggagtagcttgtatagccatgattttaaaacattatggtaccgaaattactattcaaaggttgcgtgaactttctgggacagatttagatggcacg
tctgcttttggaataaaaaaaacatttgaaaaattaggatttgatgcaccagcattcaaagctggtgatgaaacatggcaagaaaaagatatacccttgcctttg
atagctcacataataagtgaacaaaagtatcaacactacgtagtggtttataaagttaaaggtgatgagatttggattgctgacccagcaaagggaaagattag
aaaaactatttctgaattttctaaagagtggacaggtgtcttactttttcctaaaccaaaagcagaatacaaaccgtctattgaaagagtagatagtttatcaacgt
tctttcctatactaataaaacagaagtcactcttcatcacgatttttggaatcttaagttcttactattttcaaggcttattggataatattattccaaatcaggctcggt
cgactttaaatattctttccataggacttatttttgtttatctttttcgtgtgctttttgaatatagtcgtagctatcttctacttttaattgggcaaagaatgagtatgagca
taatgcttggttattttaaacacgttttgtcactacctctgagtttctttgcaaccagaaagtcaggagaaattatttctcgcttcttggatgctaataaaattattgatg
ctcttgctagtgccaccttatctttaattctggatattggaatggtaattctggttggcacgacacttgcgattcaaagtactcagcttttttttgctcacccttgccttt
ctaccttttttatattttggtagtatatgtgtttattaggagttacgataaagcaaatacagaagaaatgagtgcaggagctgaagttaattctagtattattgaaagtc
taaaaggaattgaaactattaaatcttacaatggagaaaatcatgtctatgatcgtgtagattcagaatttgtaactttaatgaaaaagtcttttaaatcggtcacac
ttgataatgtacaacagagtttaaaaatggttattgaacttataagtagtgtattgatactatggctagggtcaagttatgttatagatggaaaaataagtctagga
caattaattacctataatgctttacttgtatttttttactgaacctttacaaaatattattaatttgcaagtgaaaatgcaaaaagcacgtgtagcaaataaacgtttgaa
cgaaatcatgtcaatatctccagaacaaagaaatacgaatattaatatatcgaaaaatatatttaataaggatataaaattagataaagtaagtttttcttataatat
gaagcttcccgttttaagagatgtttctttagaaatatattccaaaagtaaggttgctcttgttggtgtgagcggttcaggcaagtctacactagctaaactattagt
aaaattctatgatccctctgagggaaatatcacttatggtgatataaattgtcaagatattgaaaatcataaattaagaaatcatgttacttatgttcctcaggaatct
ttctttttttaatggtacaattatagataatttaacttttggtcttagtcatcaaccagagtttgaaaaaatttttagagcatgtaaagctgcttgtcttgttgattttattaa
ccaacaacctttaagatttgattcagttcttgaagaaggaggaaataatctatcaggaggacaaaagcaacgcttagcaatagctagagctattttaaatgattc
tgaaataattatttttgatgaagcaactagtggtcttgataccttattagaaaaagagattttagaatatttaattaagttacaggataaaactatcatttttattgccca
ccatctatcaatagctaaagcctgtgatgaaatcattgttctagatcaaggaatattggttgggagaggaacacacgaagaattatctgaaaaagagggtgtat
ataggagattattaaacgcataacaacttacggagtaatacaaacatgaatcaaaataattggcaaaatacttcacagttctatagtcaaagtcacaaacatttct
atcgctggattatttatcctacagtagttttatttctttttattcttctttttttattcttcgggaagaaagaaattattattaaatcccaagcacaaattacatcagatttag
tttctaaaatacagattccaattgaatctaaaataatagaaaataaactttctgaaaatttgtctgtgaaaaaaggggaagcattagttaagcttgatatagagtct
cttaaacaacaaaaaaagcagtttagaaaaggaaattcctttagttcaaaaccaaattttagctaatcaaactttcattgatagtctgaagcaaggaaaaagtttgtt
cacttcagatgatgattttggttatagtaatcaattgcaaagcttactatcggagaatactgcttctgattatgcatttaaacaaagcataattgatcatgagagtga
aaaaagtacttatcaaaattctaaaaattcaattcagaaaagcatctctaaaaaacaagtagaaaaaaatgattgggaaaaaataaaaagtgcttggagttctc
agtcaaatctttcgggggcttcagaagatactacttcacaatatttgaattttcaagaacaactaaaggctagttcaaaagaagaaaaagaacaagtaaaggta
actatttcttctagtattaatgataaaatttcacaaattgaccaggaattggaacagttagaaatggagcagtcaaaactaacacctccagcttcttacgataatg
agaaaagtagtcaggaatataaaaagaaacagcttgttgaacaaactattgcaacggcaaagcaaaaaagaatagaattcaaagaagcgcaagaaaaata
taatttagaacttcaagaagtcaataagcaaatacaagatgaaataattacctcacctattgatggttttgttcatattaattataatgtcaaagatcagaagattatc
cctaagggagaagttattgccgaaatttatccagaaattaaaccagggaaaattgaatttacttcccaaatcgaagcatccgacttaacacaagttaaatcagg
gatgcgggttcattttaaacttgattcaaagggaaatagccctatcataatggagggaaaaatcaaagaaatctctgcgaatgctgaaacttcggagcgagga
agcttttatgttgtaaaagggttcttaaagcagacaaataaaactccttttaatagtcgatatggcttaaacggtcgattatcacttattgttggtaaaaaatcgtatt
ttaatgttttaaaagaaatgattataaaaaactaaaaatatctagtttttaattagcagtttatcacatgtaacaatttacaaaatgctcattgtaaaagtgtcactgct
gctagtggcacttttataatttttagatcgaattcgatatcaagcttatcgataccgtcgacctcgagtgcatatttaacttcctaaagatttcaaaatcaaagttact
ttcttgttgttttaaattttacactttttgtaatgaactgtcaaactttaaaaagtagaaaatcaatttgcttgaattcacaattgcaaaaagatatactcaaattacaag
agagaattacaaatacaacttaagtattcaattcataaagcaattgcacagttctattaaggaggaaaaaatgaaagaattatcagaaaaagaattacgagaat
gcgttggcggtggaacttgggatgatattggtcaaggaataggaagagtcgcttattgggttggaaaagccatgggaaatatgagcgatgttaatcaagcttc
tagaattaatagaaaaaagaaacactaa

lagA: 577-741; *lagC*: 840-1171; *lagD*: 1177-3288; *lagE*: 3311-4681; P4+*lagA*: 4835-5212

>lcnG gene cluster in pleiss-C$\beta_{0v1/3}$

Six AT repeats are inserted between 576 nt and 577 nt of pleiss-Cβ_0

>lcnG gene cluster in pleiss-C$\beta_{0v1/5}$

Eight AT repeats are inserted between 576 nt and 577 nt of pleiss-Cβ_0

>lcnA gene cluster and gusA3 reporter in pleiss-lcnA-GusA3

tatgagataatgccgactgtactttttacagtcggttttctaatgtcactaacctgccccgttagttgaagaaggtttttatattacagctccaagatctttgctta
atcaatggcacgtctcttaaattttagcatggcactttaactaataaaagaatggctagtaatttgcttagccattcttttattagttctgttgatttattagaatagt
cgtccccacgacataattaaaccaccacctaaatttttgtttgatgctttggaacttgtaatgaatttatacagttcagataaatcatggttaagtgttcgatttat
agctaatggagttagggcttttctgaggtttaaaataatttttggtaaatattcagaattgtcaagcgccttttctgcaatattcagagcatttctttcctcttgact
aatgtctttttcaagggcggtagccaacatacttcttagctcgttttcaaattctatttgtttCtttttcataataatctccttatttttataaatctattatagattatat
aaaaataaaactcgttaacaaatgttaataactctactgattgcctcttcccattattttatctaaaaagtaattgaaataagttttctttcctataatggttactgtt
ttcccttgaagaccatatcggatatttgggaggtcttttgcattgatagtggttgtcgcagaaactttataagcatttccctctttaaaagctgtgggagcacta
tctatttggttgattattccagttaaaatttcaggttgaggtaaattttgctgtactgtaaatctcactttttgaccgactttcattccagaaatttgagtacttggga
tataacttgtcagattaacttgtgtttctgactttaataaaggatagatttctgcgataggtgttccaatcggaatctttttcattcctaaaatatctgggagcaca
tgcaggactccagcctgttccgcaaaaacttgactgtattggtcatcttgtttttgcaagctaatttttgtttctaaatcagtaagggtactatttaggtctgtcat
ttctttatttgaagctgaaagtgcttgactttttaaagttaagatttgactagattggctattatcataagcattacttcctgttaagctactttcttgcacattaaag
gattgaatttggctttttaagctgtctttactcgaagctaaacctgataaaaattgactttttaaagactcctcttgacttttagccgcttgcttagcagtttcatct
ggattttttgatttttaaatcggcctctaaagtcgcttgttgggcttggtaactgttatattgagagaggtagggattatcttgtgaaactccaccaccactcg
ataccgcattttcgatttcagagtaattctgaatagcttgctggagtgttgccacctgattttgaatggcttgcttttgactctcggtacttttattttgatcttctac
ggcttgatttgacttttgtatggttgcttcaagactctttacttgtgcctcatagttttcaaaacttttctcatagccaaaactatcggcagtgggaaattcatttttt
tcgttggtcaaacttctttggagaaggtcgagttgtactttttttgtctaacgcttgtttcttttgagtcagtagctcactgagttgggtctgttctggtgtcccgtta
tatttgagaagtaagctattttctttaaccgcttcgccttctttgaggttattctcaatgattggattggcattcgttgattgaattttagcaactatttttgtgggttc
aatacttcctgtacttattacggtcaactccttatgggcaaagaaagtaaaaattactcctccaacaagaaggataaaaagtggaagaataattaaggtcg
agaaatttcgatagcgtttatcataaagctctgaactttccagtaatttttttatcaaacatctttttctcctgattcagttatggtaaagttgttcataaaagccattt
tgtgcaagcaggtcaacatgtgagccactctctatcacttttccttgatcgacaacaataattcgatgactcatttccgccacagagaggcggtgagcaat
gaaaataatggttttatccaagggcaacaagttctttaatattttcttctctgtaatcatatcaaggttactggttgcttcatctaaaatgaggattttggcaggg
gaaaggagcgcacgagctaaagcaatgcgttgtttttgtcccccgatagactacttgcatcacttgaaagttcggtctgatagcccaactgcatttgttca
atatctgcacggatttctgccaattccaccgctttaagaatctcttcttgtgatgcattctcattagctcctagaagtagattatctaaaattgaaccagtaaaa
atataaggttgttggggaagatagttgattaatcttcatagttgattaatcttcggagttggtgtttatcaaactgttgcaggtcaattccacctaaagtgatggt
gccagaagtgggttgaaagaagttgaccaataatttaacaagggtactctttcctgaaccactcatgcccacaatagtcaatttttcattttctttaatagaga
gttctatctcagataagacttttctgccaaaaccatattgatatgaaatatcggacatgtttaagttaaaatgtgagagggacagttctgttttcttttcctcaaat
tcactgggtacaagatagacctcatttaatcgttcattggctacccttgccttttgtagttttgtttgaaggttaatgatattggtaattggatttgtaaagtaaga
aagcagggcattaaaagtaatcaattgtccgagcgtaatttttttgacttattactaatgtggcaccaaaccataaaatggtgacactcaatgttagttgtataa
ttgctttaattaagccttgaatagcttctgatttttgtaaagtgaaagcctttttttagataacttgcaaattcgtagtcaatttttttgatatctttcttgttcactggca
agtgcctttatagtttcaatcccattgatatcttcaataattgaggagtttaagacggcattggtctgcatgacttcatggttttgtttttcaaaaaggggcgtaa
aaataataataacaacaatatagagtgggattgccaaaagaacgagaagaaagagttgcatattttgaaggcctaaaattagtcctgtcatgacgacaat
cgtcaaatctaaaaagagcgaaagaatcgttgaggcaatagcatctaaaatagaactcgcatcggaaaaccgactggtaatttctcctgttcttcgggtc
gaaaagaaagacatggaagttggaaaatgtgtctaatataagaaagaatgacatcaatagctaatctttgagaaagaacgttcaataagaaggccttag
caaattctaagacctgttggataatataggttaacaatagccctactgagataatccctaaagttcccattaaggcatttggaatatagctgtcaatcatgctc
tggaggtagtaagagcctagaatattaatcagtgttacaatgaatgaggcaataacaatattgagaatgactttcttttgacgggtgataattgggataaaa
gataataatgaggaagctttttctttagtgggatgataagatggtgtagtcgaaagaaataaactaatcccagtccattccgataaaaaagcttcttttgataa
ttttgtcattttaatggttgggtcaggatcagcaatgaataccgaatttttatttgcgccagtgatcacataataatgtgggtatttttggtctttaatgacatgag
caataaatgggtaaggagcgtttttcatttcaaaaaggcttgcatctgttcttagggcctgaactgaaaactctaatatttcagctgcctttttttatccctaaag
cggaggttccttcgattgttgtacctgcaagtaagcgcaatgaagcgagagatttttctgtgccataagactttaaaatcattgataaggcagcacagcca
cagtccatttcatctacttgtgaggtataatttttctttttaaatttcatcttttcttaactttctgagttaaaattttagtaaatcactggaaccaataatattgtttgcg
gttcagttacagagaatttcaataaatctattttagtttaaaatcgaagaaaaagatagtgaaaataataaggacttagtagtttaaacctagataaatgaaaa
taatattacttactgatgaaaaatcaattaaattttaatattgtttcagatgaagaactttcagaagctaacggaggaaaattaacatttattcaatcgacagcg
gctggagatttatattacaatactaatacacacaaatatgtttaccaacaaactcaaaacgcttttggggctgctgctaataccattgttaatggatggatgg
gtggcgctgctggaggtttcgggttgcaccattgagtcgacctcgagtgcatattttcggcaatcttctcaatgagatgctcttcagcatgttcaatgatgtc
gatttttttattaaaacgtctcaaaatcgtttctgagacgttttagcgtttatttcgtttagttatcggcataatcgttaaaacaggcgttatcgtagcgtaaaagcc
cttgagcgtagcgtgctttgcagcgaagatgttgtctgttagattatgaaagccgatgactgaatgaaataataagcgcagcgtccttctatttcggttgga
ggaggctcaagggagtttgagggaatgaaattccctcatgggtttgattttaaaaattgcttgcaattttgccgagcggtagcgcttactcaaaaattatgg

ggaatttgactatgactacgttagtaaactaattgtagaagaaaaaataacagaaaataccgtcaagcgtcgagcaagtacggtctgttcatggataaatt
ggatattatcggttgtggagtagatattttaatattgaaaagtatattgtgttgactattcgaacaataataagatttataaataaatttattaaataaaaaatcact
ggcaataatatctagtgatttttatttagtgaattttgatatataaatatgatttaatactttcgtagaggttaaagaatagtaactatgttgcctatacaaaaattc
aaggagatgtctccacaaatatggaaaggcgcaaaaaatatctctttatcaagcatttattttaattatttccatcgcaatagtaagattggcacttaataaaa
aataatactcgatttttagaataaaattaaatatgatattgatttatgttactaaatatgatataatattctaataataaataaaaggagaatttttatggaatctgc
actatatccaattcaaaataaatatcggtttaacactttaatgaatggcacttggcaatttgaaactgatcctaactctgttggtcttgacgagggatggaata
aagagttgcctgatcctgaagaaatgcctgtaccaggtacgtttgcagaattaactactaagcgagaccgtaaatactatactggagacttttggtatcaaa
aagacttctttattccttcatttctaaagaagaaagaactttatatccgttttggttcggttactcatcgcgcaaaagtatttattaatggacatgaagtcggtca
acatgaaggtggtttttaccatttcaagtaaaaatttcaaattatattaattacgaccaaactaatcgtgtaactgttttagtcaataacgaattatctgaaaaa
gctattccttgcggcaccgaagaaatcttagataacggtcaaaaacttgctcaaccttattttgatttcttcaattattctggcattatgcggaatgtctggctct
tagcacttcctcaaagccaaatcactaattttaaactaaattatcaattagcaaataataaggcaacaattacctacaatatcgaggcaaataataatgctga
atttaaagtaacacttttcgataatcaaaaagaagtagcgtgtgctacttctaaaaatactagtagtttaacaattaagaatccgcacctttggagtccaaac
gatccgtattcatacaaaataaagattgaaatgctcgaagacggaaaaacagttgacgaatacacagataaaattggtatccgcacagttaaaattgtga
atgataaaatcttgctcaataatcacccaatttatttaaaaggctttggcaagcacgaagattttaatgttttaggcaaagcagttaacgaaagcattatcaaa
cgcgactacgaatgcatgaaatggattggcgctaactgttttagaagcagtcactatccttacgccgaagaatggtatcaatatgccgataaatatggcttt
ttaattattgatgaagtacccgctgttggtcttaatcgttcaataactaactttcttaatgtaactaattctaatcagtcgcactttttgcttcgaaaactgtgcct
gaattaaaaaaggtccatgaacaagaaataaaagaaatgatcgatcgcgaccagcgtcacccttcagtgattgcctggagtttattcaatgaaccagaat
caactactcaagaatcctatgactatttcaaagatatttttgcctttgcgagaaaattggatccacaaaatcgtccttatactggaactttagttatgggtagcg
gtccaaaagtggataagcttcacccactttgtgactttgtctgcttaaaccgttattatggttggtacgttgctggtggtcctgaaatcgttaatgctaaaaag
atgctggaagatgaactagacggctggcaaaacttaaagcttaataaaccatttgtctttactgagtttggcgctgatacattatcttcttctcatcgccttcca
gatgaaatgtggagccaagaatatcaaaatgaatattatcaaatgtattttgatatatttaagaaatatccatttatttgtggcgaattagtttggaactttgctg
actttaagacgagtgaaggaatcatgcgtgttggtggtaacgataaaggaatttttactcgcgatcgtgaacctaaagatattgcctttaccttgaaaaaga
gatggcaacaattaaattaa

lciA: 503-207; *lcmA*: 2001-577; *lceA*: 4180-2492; *lcnA*: 4491-4718; *gusA3*: 5617-7413

> lcnG immunity gene and gusA3 reporter in pleiss-IG-GusA3

tactcaaaaattatggggaatttgactatgactacgttagtaaactaattgtagaagaaaaaataacagaaaataccgtcaagcgtcgagcaagtacggtc
tgttcatggataaattggatattatcggttgtggagtagatattttaatattgaaaagtatattgtgttgactattcgaacaataataagatttataaataaatttatt
aaataaaaaatcactggcaataatatctagtgatttttatttagtgaattttgatatataaatatgatttaatactttcgtagaggttaaagaatagtaactatgtt
gcctatacaaaaattcaaggagatgtctccacaaatatggaaaggcgcaaaaaatatctctttatcaagcatttattttaattatttccatcgcaatagtaaga
ttggcacttaataaaaaataatactcgatttttagaataaaattaaatatgatattgatttatgttactaaatatgatataatattctaataataaataaaaggaga
atttttTtgtttaataatatagtagtttttataaattttttaagctttgtatttattcttgtaggtgtagatattaaatataatgacaatcggataaagatagtacatgtt
actttttttattagtttcattttagtaatgctaacaagtttaatatcacataatagcattgcatatagcttatcacaaattttggaaattttatgtataatttgtattttgct
tttgttttatattttgaaaaaaactaactcactaagtaatagagctaatgtggtatttattatttttatcgttactcaagttattataatcataaatcaattatttattag
gtgattcgtttagttatcggcataatcgttaaaacaggcgttatcgtagcgtaaaagcccttgagcgtagcgtgctttgcagcgaagatgttgtctgttagat
tatgaaagccgatgactgaatgaaataataagcgcagcgtccttctatttcggttggaggaggctcaagggagtttgagggaatgaaattccctcatggg
tttgattttaaaaattgcttgcaattttgccgagcggtagcgcttaactggaataatcaaccaaatagatagtgctcccacagcttttaaagagggaaatgctt
ataaagtttctgcgacaaccactatcaatgcaaaagacctcccaaatatccgatatggtcttcaagggaaaacagtaaccattataggaaagaaaacttat
ttcaattactttttagataaaataatgggaagaggcaatcagtagagttattaacatttgttaacgagttttatttttatataatctataatagatttataaaaataa
ggagattattatggaatctgcactatatccaattcaaaataaatatcggtttaacactttaatgaatggcacttggcaatttgaaactgatcctaactctgttgg
tcttgacgagggatggaataaagagttgcctgatcctgaagaaatgcctgtaccaggtacgtttgcagaattaactactaagcgagaccgtaaatactata
ctggagacttttggtatcaaaaagacttctttattccttcatttctaaagaagaaagaactttatatccgttttggttcggttactcatcgcgcaaaagtatttatt
aatggacatgaagtcggtcaacatgaaggtggtttttaccatttcaagtaaaaatttcaaattatattaattacgaccaaactaatcgtgtaactgttttagtca
ataacgaattatctgaaaaagctattccttgcggcaccgaagaaatcttagataacggtcaaaaacttgctcaaccttattttgatttcttcaattattctggca
ttatgcggaatgtctggctcttagcacttcctcaaagccaaatcactaattttaaactaaattatcaattagcaaataataaggcaacaattacctacaatatc
gaggcaaataataatgctgaatttaaagtaacacttttcgataatcaaaaagaagtagcgtgtgctacttctaaaaatactagtagtttaacaattaagaatc
cgcacctttggagtccaaacgatccgtattcatacaaaataaagattgaaatgctcgaagacggaaaaacagttgacgaatacacagataaaattggtat
ccgcacagttaaaattgtgaatgataaaatcttgctcaataatcacccaatttatttaaaaggctttggcaagcacgaagattttaatgttttaggcaaagcag
ttaacgaaagcattatcaaacgcgactacgaatgcatgaaatggattggcgctaactgttttagaagcagtcactatccttacgccgaagaatggtatcaa
tatgccgataaatatggctttttaattattgatgaagtacccgctgttggtcttaatcgttcaataactaactttcttaatgtaactaattctaatcagtcgcactttt
ttgcttcgaaaactgtgcctgaattaaaaaaggtccatgaacaagaaataaaagaaatgatcgatcgcgaccagcgtcacccttcagtgattgcctggag
tttattcaatgaaccagaatcaactactcaagaatcctatgactatttcaaagatatttttgcctttgcgagaaaattggatccacaaaatcgtccttatactgg
aactttagttatgggtagcggtccaaaagtggataagcttcacccactttgtgactttgtctgcttaaaccgttattatggttggtacgttgctggtggtcctga
aatcgttaatgctaaaaagatgctggaagatgaactagacggctggcaaaacttaaagcttaataaaccatttgtctttactgagtttggcgctgatacatta
tcttcttctcatcgccttccagatgaaatgtggagccaagaatatcaaaatgaatattatcaaatgtattttgatatatttaagaaatatccatttatttgtggcga
attagtttggaactttgctgactttaagacgagtgaaggaatcatgcgtgttggtggtaacgataaaggaatttttactcgcgatcgtgaacctaaagatatt
gcctttaccttgaaaaagagatggcaacaattaaattaa

lagC: 536-868; *gusA3*: 1391-3187

>lcnA gene cluster, lagC gene and gusA3 reporter in pleiss-p774-lcnA-p774-lagC-GusA3
ggtcgacctcgagtgcatattttcggcaatcttctcaatgagatgctcttcagcatgttcaatgatgtcgatttttttattaaaacgtctcaaaatcgtttctgagacgt
tttagcgtttatatttttggttgccatttgttaacgctgtgtagtgagggggctggtataataaaataggtaaaaaaaatattcggaggaattttgaattgtttaataata
tagtagtttttataaatttttaagctttgtatttattcttgtaggtgtagatattaaatataatgacaatcggataaagatagtacatgttactttttttattagtttcatttta
gtaatgctaacaagtttaatatcacataatagcattgcatatagcttatcacaaattttggaaattttatgtataatttgtattttgcttttgttttatattttgaaaaaaact
aactcactaagtaatagagctaatgtggtatttattatttttatcgttactcaagttattataatcataaatcaattatttattaggtgatttttttcgtttagttatcggcata
atcgttaaaacaggcgttatcgtagcgtaaaagcccttgagcgtagcgtgctttgcagcgaagatgttgtctgttagattatgaaagccgatgactgaatgaaa
taataagcgcagcgtccttctatttcggttggaggaggctcaagggagtttgagggaatgaaattccctcatgggtttgattttaaaaattgcttgcaattttgcc
gagcggtagcgcttactcaaaaattatggggaatttgactatgactacgttagtaaactaattgtagaagaaaaaataacagaaaataccgtcaagcgtcgag
caagtacggtctgttcatggataaattggatattatcggttgtggagtagatattttaatattgaaaagtatattgtgttgactattcgaacaataataagatttataa
ataaatttattaaataaaaaaatcactggcaataatatctagtgatttttttatttagtgaattttgatatataaatatgatttaatactttcgtagaggttaaagaatagtaa
ctatgttgcctatacaaaaattcaaggagatgtctccacaaatatggaaaggcgcaaaaaaatatctctttatcaagcatttattttaattatttccatcgcaatagta
agattggcacttaataaaaaataatactcgatttttagaataaaattaaatatgatattgatttatgttactaaatatgatataatattctaataataaataaaaggaga
atttttatggaatctgcactatatccaattcaaaataaatatcggtttaacactttaatgaatggcacttggcaatttgaaactgatcctaactctgttggtcttgacg
agggatggaataaagagttgcctgatcctgaagaaatgcctgtaccaggtacgtttgcagaattaactactaagcgagaccgtaaatactatactggagactt
ttggtatcaaaaagacttctttattccttcatttctaaagaagaaagaactttatatccgttttggttcggttactcatcgcgcaaaagtatttattaatggacatgaag
tcggtcaacatgaaggtggtttttttaccatttcaagtaaaaatttcaaattatattaattacgaccaaactaatcgtgtaactgttttagtcaataacgaattatctgaa
aaagctattccttgcggcaccgaagaaatcttagataacggtcaaaaacttgctcaaccttattttgatttcttcaattattctggcattatgcggaatgtctggctc
ttagcacttcctcaaagccaaatcactaattttaaactaaattatcaattagcaaataataaggcaacaattacctacaatatcgaggcaaataataatgctgaatt
taaagtaacacttttcgataatcaaaaagaagtagcgtgtgctacttctaaaaatactagtagtttaacaattaagaatccgcacctttggagtccaaacgatccg
tattcatacaaaataaagattgaaatgctcgaagacggaaaaacagttgacgaatacacagataaaattggtatccgcacagttaaaattgtgaatgataaaat
cttgctcaataatcacccaatttatttaaaaggctttggcaagcacgaagattttaatgttttaggcaaagcagttaacgaaagcattatcaaacgcgactacga
atgcatgaaatggattggcgctaactgttttagaagcagtcactatccttacgccgaagaatggtatcaatatgccgataaatatggctttttaattattgatgaag
tacccgctgttggtcttaatcgttcaataactaactttcttaatgtaactaattctaatcagtcgcactttttttgcttcgaaaactgtgcctgaattaaaaaaggtccat
gaacaagaaataaaagaaatgatcgatcgcgaccagcgtcacccttcagtgattgcctggagtttattcaatgaaccagaatcaactactcaagaatcctatg
actatttcaaagatatttttgcctttgcgagaaaattggatccacaaaatcgtccttatactggaactttagttatgggtagcggtccaaaagtggataagcttcac
ccactttgtgactttgtctgcttaaaccgttattatggttggtacgttgctggtggtcctgaaatcgttaatgctaaaaagatgctggaagatgaactagacggct
ggcaaaacttaaagcttaataaaccatttgtctttactgagtttggcgctgatacattatcttcttctcatcgccttccagatgaaatgtggagccaagaatatcaa
aatgaatattatcaaatgtattttgatatatttaagaaatatccatttatttgtggcgaattagtttggaactttgctgactttaagacgagtgaaggaatcatgcgtgt
tggtggtaacgataaaggaatttttactcgcgatcgtgaacctaaagatattgcctttaccttgaaaaagagatggcaacaattaaattaaagcgctggaaaatt
tttgaaaaaaatttggaatttggaaaaaaatgggggggaaaggaagcgaattttgcttccgtactacgacccccccattaagtgccgagtgccaatttttgtgcca
aaaacgctctatcccaactggctcaagggtttgaggggtttttcaatcgccaacgaatcgccaacgttttcgccaacgtttttttataaatctatatttaagtagcttt
attttttgtttttatgattacaaagtgatacactaattttataaaattatttgattggagtttttttaaatggtgatttcagaatcgaaaaaaagagttatgatttctctgaca
aaagagcaagataaaaaattaacagatatggcgaaacaaaaagatttttcaaaatctgcggttgcggcgttagctatagaagaatatgcaagaaaggaatca
gaacaaaaaaaataagcgaaagctcgcgtttttagaaggatacgagttttcgctacttgtttttgataaggtaatatatcatggctattaaaaatactaaagctag
aaattttggatttttattatatcctgactcaattcctaatgattggaaagaaaaattagagagtttgggcgtatctatggctgtcagtcctttacacgatatggacga
aaaaaaagataaagatacatggaatagtagtgatgttatacgaaatggaaagcactataaaaaaccacactatcacgttatatatattgcacgaaatcctgtaa
caatagaaagcgttaggaacaagattaagcgaaaattggggaatagttcagttgctcatgttgagatacttgattatatcaaaggttcatatgaatatttgactca
tgaatcaaaggacgctattgctaagaataaacatatatacgacaaaaaagatattttgaacattaatgattttgatattgaccgctatataacacttgatgaaagc
caaaaaagagaattgaagaatttacttttagatatagtggatgactataatttggtaaatacaaaagatttaatggcttttattcgccttaggggagcggagtttgg
aattttaaatacgaatgatgtaaaagatattgtttcaacaaactctagcgcctttagattatggtttgagggcaattatcagtgtggatatagagcaagttatgcaa
aggttcttgatgctgaaacgggggaaataaaatgacaaacaaagaaaaagagttattgctgaaaatgtgaactttaataaaattgatttagacaattggaagag
aaaagagatatttaatcattatttgaaccaacaaacgacttttagtataaccacagaaattgatattagtgttttataccgaaacataaaacaagaaggatataaat
tttaccctgcatttattttcttagtgacaagggtgataaactcaaatacagcttttagaactggttacaatagcgacggagagttaggttattgggataagttagag
ccactttatacaatttttgatggtgtatctaaaacattctctggtatttggactcctgtaaagaatgacttcaaagagttttatgatttatacctttctgatgtagagaaa
tataatggttcggggaaattgtttcccaaaacacctatacctgaaaatgctttttctctttctattattccatggacttcatttactgggtttaacttaaatatcaataata
atagtaattaccttctacccattattacagcaggaaaattcattaataaaggtaattcaatatatttaccgctatctttacaggtacatcattctgtttgtgatggttatc
atgcaggattgtttatgaactctattcaggaattgtcagataggcctaatgactggcttttataatatgagataatgccgactgtactttttacagtcggttttctaat
gtcactaacctgccccgttagttgaagaaggtttttatattacagctccaagatctttgcttaatcaatggcacgtctcttaaattttagcatggcactttaactaata
aaagaatggctagtaatttgcttagccattcttttattagttctgttgatttattagaatagtcgtccccacgacataattaaaccaccacctaaatttttgtttgatgct
ttggaacttgtaatgaatttatacagttcagataaatcatggttaagtgttcgatttatagctaatggagttagggcttttctgaggtttaaaataatttttggtaaatat
tcagaattgtcaagcgccttttctgcaatattcagagcatttctttcctcttgactaatgtctttttcaagggcggtagccaacatacttcttagctcgttttcaaattct
atttgtttCtttttcataataatctccttatttttataaatctattatagattatataaaaataaaactcgttaacaaatgttaataactctactgattgcctcttcccattatt
ttatctaaaaagtaattgaaataagttttctttcctataatggttactgttttcccttgaagaccatatcggatatttgggaggtcttttgcattgatagtggttgtcgca
gaaactttataagcatttccctctttaaaagctgtgggagcactatctatttggttgattattccagttaaaatttcaggttgaggtaaattttgctgtactgtaaatct
cactttttgaccgactttcattccagaaatttgagtacttgggatataacttgtcagattaacttgtgtttctgactttaataaaggatagatttctgcgataggtgttc

caatcggaatctttttcattcctaaaatatctgggagcacatgcaggactccagcctgttccgcaaaaacttgactgtattggtcatcttgtttttgcaagctaatttt
tgtttctaaatcagtaagggtactatttaggtctgtcatttctttatttgaagctgaaagtgcttgactttttaaagttaagatttgactagattggctattatcataagc
attacttcctgttaagctactttcttgcacattaaaggattgaatttggctttttaagctgtctttactcgaagctaaacctgataaaaattgactttttaaagactcctc
ttgacttttagccgcttgcttagcagtttcatctggatttttttgatttttttaaatcggcctctaaagtcgcttgttgggcttggtaactgttatattgagagaggtagg
gattatcttgtgaaactccaccaccactcgataccgcattttcgatttcagagtaattctgaatagcttgctggagtgttgccacctgattttgaatggcttgcttttg
actctcggtacttttattttgatcttctacggcttgatttgacttttgtatggttgcttcaagactctttacttgtgcctcatagttttcaaaacttttctcatagccaaaact
atcggcagtgggaaattcatttttttcgttggtcaaacttctttggagaaggtcgagttgtactttttttgtctaacgcttgtttcttttgagtcagtagctcactgagttg
ggtctgttctggtgtcccgttatatttgagaagtaagctattttctttaaccgcttcgccttctttgaggttattctcaatgattggattggcattcgttgattgaatttta
gcaactatttttgtgggttcaatacttcctgtacttattacggtcaactccttatgggcaaagaaagtaaaaattactcctccaacaagaaggataaaaagtggaa
gaataattaaggtcgagaaatttcgatagcgtttatcataaagctctgaactttccagtaatttttttatcaaacatctttttctcctgattcagttatggtaaagttgttc
ataaaagccattttgtgcaagcaggtcaacatgtgagccactctctatcacttttccttgatcgacaacaataattcgatgactcatttccgccacagagaggcg
gtgagcaatgaaaataatggttttatccaagggcaacaagttctttaatattttcttctctgtaatcatatcaaggttactggttgcttcatctaaaatgaggattttgg
caggggaaaggagcgcacgagctaaagcaatgcgttgtttttgtccccccgatagactacttgcatcacttgaaagttcggtctgatagcccaactgcatttgt
tcaatatctgcacggatttctgccaattccaccgctttaagaatctcttcttgtgatgcattctcattagctcctagaagtagattatctaaaattgaaccagtaaaa
atataaggttgttggggaagatagttgattaatcttcatagttgattaatcttcggagttggtgtttatcaaactgttgcaggtcaattccacctaaagtgatggtgc
cagaagtgggttgaaagaagttgaccaataatttaacaagggtactctttcctgaaccactcatgcccacaatagtcaatttttcattttctttaatagagagttcta
tctcagataagacttttctgccaaaaccatattgatatgaaatatcggacatgtttaagttaaaatgtgagagggacagttctgttttcttttcctcaaattcactggg
tacaagatagacctcatttaatcgttcattggctacccttgccttttgtagttttgtttgaaggttaatgatattggtaattggatttgtaaagtaagaaagcagggca
ttaaaagtaatcaattgtccgagcgtaatttttttgacttattactaatgtggcaccaaaccataaaatggtgacactcaatgttagttgtataattgctttaattaagc
cttgaatagcttctgatttttgtaaagtgaaagccttttttagataacttgcaaattcgtagtcaatttttttgatatctttcttgttcactggcaagtgcctttatagtttca
atcccattgatatcttcaataattgaggagtttaagacggcattggtctgcatgacttcatggttttgtttttcaaaaaggggcgtaaaaataataataacaacaat
atagagtgggattgccaaaagaacgagaagaaagagttgcatattttgaaggcctaaaattagtcctgtcatgacgacaatcgtcaaatctaaaaagagcga
aagaatcgttgaggcaatagcatctaaaatagaactcgcatcggaaaaccgactggtaatttctcctgttcttcgggtcgaaaagaaagacatgggaagttgg
aaaatgtgtctaatataagaaagaatgacatcaatagctaatctttgagaaagaacgttcaataagaaggccttagcaaattctaagacctgttggataatatag
gttaacaatagccctactgagataatccctaaagttcccattaaggcatttggaatatagctgtcaatcatgctctggaggtagtaagagcctagaatattaatca
gtgttacaatgaatgaggcaataacaatattgagaatgactttcttttgacgggtgataattgggataaaagataataatgaggaagctttttctttagtgggatga
taagatggtgtagtcgaaagaaataaactaatcccagtccattccgataaaaaaagcttcttttgataattttgtcattttaatggttgggtcaggatcagcaatgaa
taccgaatttttatttgcgccagtgatcacataataatgtgggtatttttggtctttaatgacatgagcaataaatgggtaaggagcgtttttcatttcaaaaaggctt
gcatctgttcttagggcctgaactgaaaactctaatatttcagctgcctttttttatccctaaagcggaggttccttcgattgttgtacctgcaagtaagcgcaatga
agcgagagatttttctgtgccataagactttaaaatcattgataaggcagcacagccacagtccatttcatctacttgtgaggtataatttttctttttaaatttcatct
tttcttaactttctgagttaaaattttagtaaatcactggaaccaataatattgtttgcggttcagttacagagaatttcaataaatctattttagtttaaaatcgaagaa
aaagatagtgaaaataataaggacttagtagtttaaacctagataaatgaaaataatattacttactgatttttggttgccatttgttaacgctgtgtagtgagggg
gctggtataataaaataggtaaaaaaaatattcggaggaattttgaaatgaaaaatcaattaaattttaatattgtttcagatgaagaactttcagaagctaacgga
ggaaaattaacatttattcaatcgacagcggctggagatttatattacaatactaatacacacaaatatgtttaccaacaaactcaaaacgcttttggggctgctg
ctaataccattgttaatggatggatgggtggcgctgctggaggtttcgggttgcaccattga

p774-*lcnA*: 9381-9693; p774-*lagC*: 121-538; *gusA3*: 1321-3117

Supplementary Table 4: Summary of variables and parameters used in simulations.

Symble	Definition
C_α	Population density of strain Cα
C_β	Population density of strain Cβ
K_s	Population density of strain Ks
K_r	Population density of strain Kr
K_p	Population density of strain Kp
N	Nutrient concentration
α	Concentration of peptide α
β	Concentration of peptide β
G	Normalized productivity of bacteriocin lcnG
A	Productivity of bacteriocin lcnA
g_i	Maximum growth rate of strain i
N_i	Half-saturation constant of strain i
γ_i	Yield from nutrient for strain i
p_j	Production rate of molecule α, β, and lcnA
$k_{\alpha\beta}$	Synthetic rate of lcnG
d_j	Degradation rate of molecule lcnA and lcnG
$I_{i,A}$	Maximal strength of lcnA-induced inhibition on strain Cα (Cβ)
$K_{i,A}$	Half killing parameter of lcnA on strain Cα (Cβ)
$I_{Ks,G}$	Maximal strength of lcnG-induced inhibition on strain Ks
$K_{Ks,G}$	Half killing parameter of lcnG on strain Ks
$I_{Kp,G}$	Maximal strength of lcnG-induced inhibition on strain Kp
$K_{Kp,G}$	Half killing parameter of lcnG on strain Kp
μ	Mean of Gaussian distribution
σ^2	Variance of Gaussian distribution

[1] The subscript i refers to strains Cα, Cβ, Ks, Kr and Kp.

[2] The subscript j refers to peptide α, β, bacteriocins lcnG or lcnA.

Supplementary Table 5: Parameter values of mono-cultured strains.

**Strain**	**Maximum growth rate** g_i	**Half-saturation constant** N_i	**Yield coeffi.** γ_i
Cα	1.33	0.28	3.93
Cβ	1.04	0.09	3.99
Ks	1.07	0.15	3.98
Kr	1.05	0.14	3.87

[1] i denotes for Cα, Cβ, Ks, and Kr.

Supplementary Table 6: Summary of dynamics parameters in Cα-Cβ-Ks ecosystem.

**Parameter**	**Value**
$p_\alpha = p_\beta$	33.1
$k_{\alpha\beta}$	5.4
d_G	0.85
p_A	25.3
d_A	12.3
$I_{C\alpha,A}$	0.73
$K_{C\alpha,A}$	0.12
$I_{C\beta,A}$	0.63
$K_{C\beta,A}$	0.14
$I_{Ks,G}$	0.76
$K_{Ks,G}$	0.38

Supplementary Table 7: Means and coefficients of variation obtained from experiments.

**Dilution Concen.**	**Mean population of strains(cell mL^{-1})**	**Coefficient of variation** (CV=σ_i/μ_i)			
		Ks	Kp(pH≥7)	Kp(pH≤6)	Kp(No control)
OD_{600}=1	$n_0 = 2 \times 10^8$	0	0	0	0
OD_{600}=10^{-1}	n_1=$\mathcal{N}(\mu_1,{\sigma_1}^2)$	0.042	0.038	0.04	0.03
OD_{600}=10^{-2}	n_2=$\mathcal{N}(\mu_2,{\sigma_2}^2)$	0.042	0.038	0.04	0.03
OD_{600}=10^{-3}	n_3=$\mathcal{N}(\mu_3,{\sigma_3}^2)$	0.03	0.015	0.01	0.025
OD_{600}=10^{-4}	n_4=$\mathcal{N}(\mu_4,{\sigma_4}^2)$	0.03	0.015	0.01	0.025
OD_{600}=10^{-5}	n_5=$\mathcal{N}(\mu_5,{\sigma_5}^2)$	0.12	0.13	0.17	0.18
OD_{600}=10^{-6}	n_5=$\mathcal{N}(\mu_6,{\sigma_6}^2)$	0.12	0.13	0.17	0.18
OD_{600}=10^{-7}	n_7=$\mathcal{N}(\mu_7,{\sigma_7}^2)$	0.12	0.21	0.12	0.09
OD_{600}=10^{-8}	n_8=$\mathcal{N}(\mu_8,{\sigma_8}^2)$	0.12	0.21	0.12	0.09

[1] $\mathcal{N}(\mu_i,{\sigma_i}^2)$ denotes for a random number following Gaussian distribution, where μ_i ($\mu_i = n_{i-1}/10$) and ${\sigma_i}^2$ are the mean and variance of cell number upon the i-th dilution.

Supplementary Table 8: Means and standard deviations obtained from experiments and used in sampling the initial Cα fraction in the Cα-Cβ co-culture.

**Dilution Concen.**	**Stochastic Cα fraction** f_i=Cα/(Cα+Cβ)	**Standard Deviation σ_i**			
		Ks	Kp(pH≥7)	Kp(pH≤6)	Kp(No control)
OD_{600}=1	$f_0=\mathcal{N}(\mu,{\sigma_0}^2)$	0	0	0	0
$OD_{600}=10^{-1}$	$f_1=\mathcal{N}(\mu,{\sigma_1}^2)$	0.01	0.02	0.01	0.01
$OD_{600}=10^{-2}$	$f_2=\mathcal{N}(\mu,{\sigma_2}^2)$	0.01	0.02	0.01	0.01
$OD_{600}=10^{-3}$	$f_3=\mathcal{N}(\mu,{\sigma_3}^2)$	0.02	0.01	0.01	0.02
$OD_{600}=10^{-4}$	$f_4=\mathcal{N}(\mu,{\sigma_4}^2)$	0.02	0.01	0.01	0.02
$OD_{600}=10^{-5}$	$f_5=\mathcal{N}(\mu,{\sigma_5}^2)$	0.21	0.18	0.19	0.18
$OD_{600}=10^{-6}$	$f_6=\mathcal{N}(\mu,{\sigma_6}^2)$	0.21	0.18	0.19	0.18
$OD_{600}=10^{-7}$	$f_7=\mathcal{N}(\mu,{\sigma_7}^2)$	0.23	0.23	0.24	0.24
$OD_{600}=10^{-8}$	$f_8=\mathcal{N}(\mu,{\sigma_8}^2)$	0.23	0.23	0.24	0.24

[1] $\mathcal{N}(\mu,{\sigma_i}^2)$ denotes for a random fraction following Gaussian distribution, where μ ($\mu = 0.5$) and ${\sigma_i}^2$ are the mean and variance of the fraction upon the i-th dilution.

Supplementary Table 9: Growth parameters of Ks and Kp when growing in the supernatants of Cα-Cβ' co-culture.

**Strain**	**Medium**	**Maximum growth rate** (t≥1.5 h)	**Half-saturation const.**	**Yield coeffi.**
Ks		0.88	0.0069	3.24
Kp	pH $\geq$ 7	0.91	0.10	1.77
	pH $\leq$ 6	0.92	0.10	2.52
	No control	0.84	0.0084	3.40

Supplementary Table 10: Averaged growth rates of the strains Ks and Kp in three pH-controlled settings.

**Strain**	**Medium**	**Ave. growth rate Cα-Cβ supernatant**	**Ave. growth rate Cα-Cβ' supernatant**	**Relative growth rate**
Ks	pH $\geq$ 7	0.469	0.727	0.645
	pH $\leq$ 6	0.500	0.664	0.753
	No control	0.557	0.840	0.663
Kp	pH $\geq$ 7	0.558	0.767	0.728
	pH $\leq$ 6	0.764	0.787	0.971
	No control	0.753	0.846	0.890

Supplementary Table 11: Parameters for the Cα, Cβ and Kp monocultures in different pH settings.

**Medium**	**Strain**	**Max. growth rate**	**Half-saturation const.**	**Yield coeffi.**
pH≥7	Cα	1.13	0.10	1.56
	Cβ	1.06	0.082	1.68
	Kp	1.10	0.10	1.57
pH≤6	Cα	1.19	0.10	2.21
	Cβ	1.19	0.10	2.29
	Kp	1.21	0.10	2.23
No control	Cα	1.28	0.10	3.34
	Cβ	1.12	0.0068	3.47
	Kp	1.13	0.017	3.19

Supplementary Table 12: Parameters for the Cα-Cβ-Kp ecosystem in three different pH settings.

Parameter	pH≥7	pH≤6	No control
p_α=p_β	7.2	18.1	37.2
$k_{\alpha\beta}$	0.5	2.0	15.4
d_G	0.2	0.5	1.1
p_A	0	183.0	0 (t< t_c)<br>27.3 (t≥ t_c)
d_A	-	42.7	12.3 (t≥ t_c)
$I_{C\alpha,A} = I_{C\beta,A}$	-	0.81	- (t< t_c)<br>0.81 (t≥ t_c)
$K_{C\alpha,A} = K_{C\beta,A}$	-	1.19	1.19 (t≥ t_c)
$I_{Kp,G}$	0.79	0.32	0.79 (t< t_c)<br>0.49 (t≥ t_c)
$K_{Kp,G}$	3.28	0.83	3.28

[1] The delay times t_c for the initial ODs of 10^{-2}, 10^{-4}, 10^{-6}, and 10^{-8} are 4 h, 7 h, 8 h and 10 h respectively.

4 Supplementary References

[1] Jacques Monod. The growth of bacterial cultures. *Annual Reviews in Microbiology*, 3(1):371–394, 1949.

[2] Rima B Franklin, Jay L Garland, Carl H Bolster, and Aaron L Mills. Impact of dilution on microbial community structure and functional potential: comparison of numerical simulations and batch culture experiments. *Applied and Environmental Microbiology*, 67(2):702–712, 2001.

[3] Yan Yan, Eiko E Kuramae, Peter GL Klinkhamer, and Johannes A van Veen. Revisiting the dilution procedure used to manipulate microbial biodiversity in terrestrial systems. *Applied and Environmental Microbiology*, 81(13):4246–4252, 2015.

[4] Michael J Gasson. Plasmid complements of *Streptococcus lactis* NCDO 712 and other lactic streptococci after protoplast-induced curing. *Journal of Bacteriology*, 154(1):1–9, 1983.

[5] Nemanja Mirkovic, Natalija Polovic, Goran Vukotic, Branko Jovcic, Marija Miljkovic, Zorica Radulovic, Dzung B Diep, and Milan Kojic. *Lactococcus lactis* LMG2081 produces two bacteriocins, a nonlantibiotic and a novel lantibiotic. *Applied and Environmental Microbiology*, 82(8):2555–2562, 2016.

[6] Y Le Loir, A Gruss, SD Ehrlich, and P Langella. A nine-residue synthetic propeptide enhances secretion efficiency of heterologous proteins in *Lactococcus lactis*. *Journal of Bacteriology*, 180(7):1895–1903, 1998.
